# The Long Term Response of Birds to Climate Change: New Results from a Cold Stage Avifauna in Northern England

**DOI:** 10.1371/journal.pone.0122617

**Published:** 2015-05-20

**Authors:** John R. Stewart, Roger M. Jacobi

**Affiliations:** 1 Faculty of Science and Technology, Bournemouth University, Talbot Campus, Fern Barrow, Poole, Dorset, BH12 5BB, United Kingdom; 2 Department of Earth Sciences, Natural History Museum, Cromwell Road, London, SW7 5BD, United Kingdom; University of Kansas, UNITED STATES

## Abstract

The early MIS 3 (55–40 Kyr BP associated with Middle Palaeolithic archaeology) bird remains from Pin Hole, Creswell Crags, Derbyshire, England are analysed in the context of the new dating of the site’s stratigraphy. The analysis is restricted to the material from the early MIS 3 level of the cave because the upper fauna is now known to include Holocene material as well as that from the Late Glacial. The results of the analysis confirm the presence of the taxa, possibly unexpected for a Late Pleistocene glacial deposit including records such as Alpine swift, demoiselle crane and long-legged buzzard with southern and/or eastern distributions today. These taxa are accompanied by more expected ones such as willow ptarmigan /red grouse and rock ptarmigan living today in northern and montane areas. Finally, there are temperate taxa normally requiring trees for nesting such as wood pigeon and grey heron. Therefore, the result of the analysis is that the avifauna of early MIS 3 in England included taxa whose ranges today do not overlap making it a non-analogue community similar to the many steppe-tundra mammalian faunas of the time. The inclusion of more temperate and woodland taxa is discussed in the light that parts of northern Europe may have acted as cryptic northern refugia for some such taxa during the last glacial. These records showing former ranges of taxa are considered in the light of modern phylogeographic studies as these often assume former ranges without considering the fossil record of those taxa. In addition to the anomalous combination of taxa during MIS 3 living in Derbyshire, the individuals of a number of the taxa are different in size and shape to members of the species today probably due to the high carrying capacity of the steppe-tundra.

## Introduction

The response of organisms to climate change is a subject that has seen an increase in attention over recent years [1–4]. Arguably, birds are the best observed organisms of all given their popularity amongst amateur ornithologists and the public, particularly through citizen science projects, in addition to professional scientists (see [5]). They have correspondingly received a great deal of attention in relation to the impact of future climate change [6–10]. The longer term response of birds to climate change, at the multi-millennial scale, has however received less attention. It is often said that bird remains are rare in the fossil record when in fact they are relatively common [11], particularly in Pleistocene cave deposits. Furthermore, bird remains contain a wealth of palaeoenvironmental information because, as the most mobile terrestrial vertebrates, they can respond quickly to rapid climatic and environmental change. Modern birds have been observed responding quickly to humanly induced climate change [7] and it has been suggested that species associations of birds will undergo re-shuffling due to climate change over the next 50 or more years, hence producing non-analogue communities [12]. It is therefore important to establish baselines of past responses to natural change.

The Quaternary, popularly known as the Ice Ages, has seen multiple natural oscillations of climate with accompanying ecological changes. At modern temperate latitudes the changes have typically involved variations from glacial stage steppe-tundra biomes to the interglacial broad-leaf forest biomes found today [13]. The way that these changes affected birds has rarely been dealt with explicitly, although a large literature detailing the avian remains from various Pleistocene sites exists (see [14]). There have been some recent publications with analyses using the metadata provided by Tyrberg [15,16]. However, these metadata analyses have not been done with any significant acknowledgment that the literature is likely to include over-ambitious or incorrect identifications.

Here we analyse the bird remains from a cave deposit (Pin Hole, Creswell Crags in Derbyshire, in Northern England) dated to the middle of the last glacial stage (Marine Oxygen Isotope Stage 3) associated with Middle Palaeolithic archaeology (see Text A in S1 File). Pin Hole, has yielded one of the largest fossil avifaunas in Europe both in terms of the quantity of remains and the claimed number of taxa represented [17,14]. Pin Hole, quaintly named after an old local custom of visitors dropping a pin into a water-filled hollow and removing a pin left by a previous visitor, is in a cave-rich gorge called Creswell Crags. The stratigraphy of the cave, the dating of the deposits, their archaeology and mammalian fauna have been re-assessed [18] since the birds were published by Bramwell [17] which has prompted the need for a revision of their remains. This is particularly the case because Bramwell used the Pin Hole fauna to suggest that woodland birds were present in Britain before the end of the last cold stage. This was suggested for both the lower (Mid Devensian) and upper (Late Devensian) horizons [17]. The Bramwell conclusions based on the Late Devensian horizon are now no longer valid as that deposit also contains Holocene material and so the woodland birds are no longer surprising [18]. This re-analysis also provides an opportunity to use a well dated, species rich, avian assemblage from the last glacial to examine some of the conclusions of the recently published avian phylogeographic studies. The latter is why we have included detailed accounts of the reasoning for the taxonomic attributions herein so that the reliability of identifications can be judged; something we feel should be encouraged, if the data is to be subsequently analysed as part of a large metadata study.

## Material and Methods

**Fig 1:**
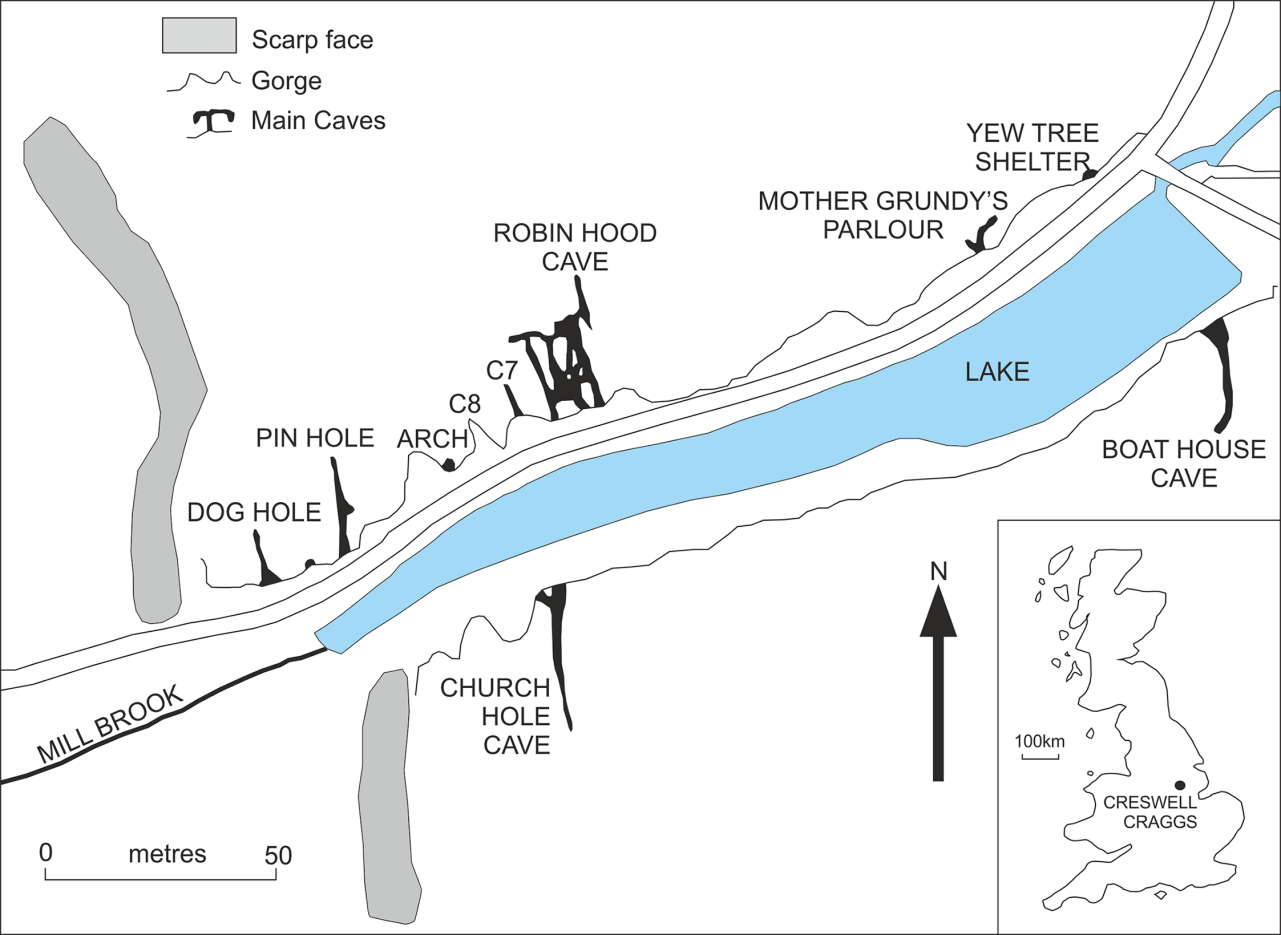
Location of Pin Hole.

**Fig 2:**
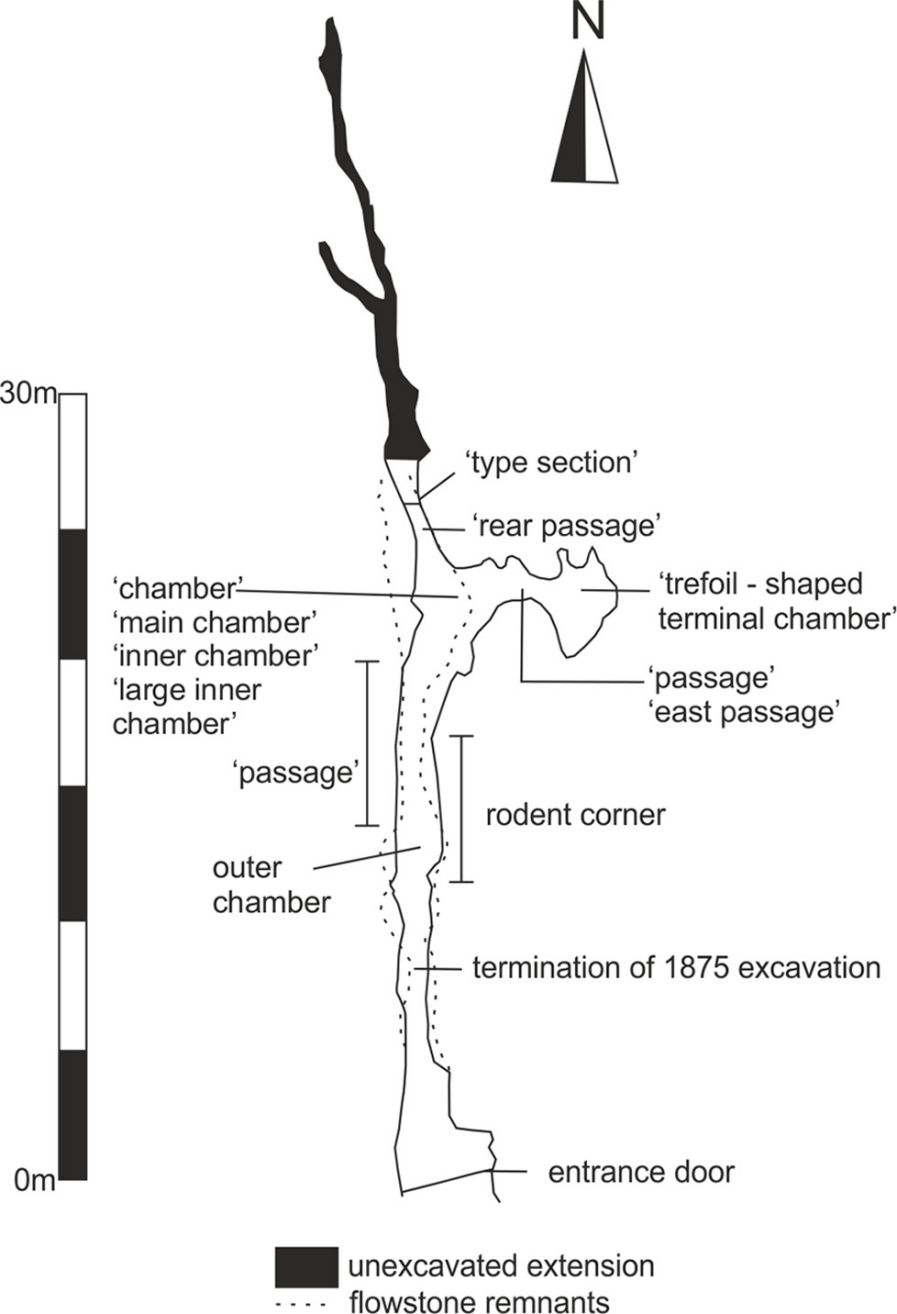
Plan of Pin Hole interior showing different passages. Also marked is the location of the remnants of the flowstone (dotted line) that was used to provide depths of artefacts and bones by Armstrong.

The following is a description of the bird remains from the Middle Palaeolithic (Marine Oxygen Isotope Stage 3) deposits of Pin Hole, Creswell Crags, Derbyshire in England. Thanks are due to the staff of the Manchester Museum for allowing access to the material described here and in particular to Kate Sherburn, John Nudds, and David Gelsthorpe. Jo Cooper and Robert Prýs-Jones of the Birds Group, the Natural History Museum in London, Tring and Per Ericson of the Department of Vertebrate Zoology, Swedish Museum of Natural History, Stockholm are thanked for providing access to their avian osteological collections. The material described here was collected by A.L. Armstrong between 1924–1936 [19–23]. The specimen numbers are all listed in the S1 File. Pin Hole is one of four caves at Creswell Crags to have yielded both Pleistocene fauna and Palaeolithic artefacts. Creswell Crags is a shallow gorge incised in the Lower Permian Magnesian Limestone on the borders of the counties of Derbyshire and Nottinghamshire in the English East Midlands. Pin Hole is on the Derbyshire (northern) side of the gorge and at its western end (Fig 1). The cave is a narrow, solutionally enlarged linear fissure some 46 metres (m) in length. This is the “main passage” and it widens locally to create the “outer” and “inner chambers”. A short passage leads off eastwards from the inner chamber (Fig 2).

**Fig 3:**
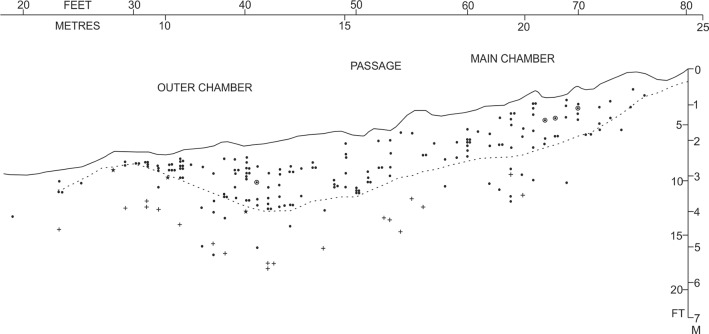
Pin Hole stratigraphy as excavated by Armstrong. Plot of all lithics recovered by Armstrong from the (main) passage. ☉ = neolithic artefacts, + = non-flint artefacts * = boundary of upper and lower cave-earths as determined from Armstrong’s notebook. The dashed line marks the lower limit of flint blade recoveries.

The material here described is that from the Middle Palaeolithic level (the lower horizon) although two categories of confidence over the stratigraphic provenance are defined—MP (Very Likely Middle Palaeolithic) and PMP (Probably Middle Palaeolithic) which is detailed in the S1 File. The division between the lower and upper horizons is defined by a line, depicted in Fig 3, below which material has been dated to MIS 3. (For more details of the stratigraphy see Text A in S1 File). The age of the assemblage has been dated by numerous radiocarbon determinations for this lower fauna and these complement electron spin resonance (ESR) and Uranium-series dates in suggesting an age for it of between 40–55 kyr BP [18,24]. These dates correspond to the early part of Marine Oxygen Isotope Stage 3 (MIS 3). A small number of avian specimens, whose provenance is unsure or come from the Upper Fauna and belonging to rare taxa, are also described below.

The bird remains were identified by means of modern bird comparative material belonging to one of the authors (JRS) and to the Natural History Museum at Tring in Hertfordshire, England and the Swedish Museum of Natural History, Stockholm. Avian anatomical description has made use of the terminology described in Baumel [25]. Stewart and Hernandez-Carrasquilla [26] published a review of literature which aids identification of bird skeletal remains and, where relevant, such literature has been consulted. Hence, Woolfenden [27] has been consulted for the identification of the Anseriformes as a whole; Woelfle [28] for ducks; Bacher [29] for swans and geese; Fitzgerald [30] for Gaviformes; Schmidt-Burger [31] for hawks; Solti [32,33] for larger and smaller falcons; Stewart [34] for the thrushes; Langer [35] for owls; Gruber [36] for storks; Kellner [37] for herons; Stewart [38] for the starlings and cranes; Kraft [39] for smaller gamebirds; Fick [40] and Cassoli [41] for pigeons, doves and sandgrouse, and Tomek and Bochenski [42] for crows. The authors’ guidelines for assigning taxonomic levels are detailed in Stewart [43].

Measurements taken on the Pin Hole and modern bird skeletal elements were done largely according to von den Driesch [44] although slightly different measurements were taken on different taxonomic groups. The variants of the measurements followed were those used by the identification literature listed above, in which measurements are illustrated with additional ones designed by the author illustrated in (Fig K in S1 File).

## Results

### Systematic Description

Here follows a systematic description of the bird taxa found in the Middle Palaeolithic (Early MIS 3) levels at Pin Hole. Table 1 lists the identified taxa, the number of individual remains and their provenance within the Pin Hole stratigraphy. For more details on the material representing the following taxa see the section in (Text C in S1 File).

**Table 1:** Bird taxa present in the MIS 3 Middle Palaeolithic horizon of Pin Hole (Number of specimens).

Taxa	MP	PMP	Unprovenanced / Not MP	Total
**Gaviformes**				
*Gavia* cf. *stellata* / *arctica*	-	1	-	1
**Ciconiformes**				
*Ciconia* cf. *ciconia* / *nigra*	1	-	-	1
*Ardea* cf. *cinerea*	1	-	2	3
**Anseriformes**				
*Branta* cf. *bernicla*	1	-	-	1
Undet. large Anseriformes	7	7	-	14
*Melanitta* cf. *fusca*	-	1	-	1
*Melanita* sp	-	1	-	1
*Anas cf*. *crecca*	-	1	-	1
*Clangula hyamalis / Bucephala clangula*	-	1	-	1
Undet. Anatinae	17	13	-	30
**Accipitriformes**				
*Buteo* cf. *rufinus*	-	1	-	1
**Falcomiformes**				
*Falco* cf. *tinnunculus*	1	-	-	1
Undet. small *Falco* sp.	2	2	-	4
Undet. large *Falco* sp.	-	1	-	1
**Charadriiformes**				
*Stercorarius* cf. *parasiticus* / *longicaudatus*	1	-	-	1
cf. *Charadrius morinella*	2	-	-	2
Undet. Scolopacidae	1	1	-	2
Undet. Charadriidae	1	-	-	1
*Alca torda* / *Fratercula arctica*	-	-	1	1
**Columbiformes**				
*Columba* cf. *palumbus*	1	-	-	1
**Strigiformes**				
*Bubo* cf. *bubo* / *scandiaca*	-	1	1	2
cf. *Asio flammeus*	-	2	-	2
*Surnia ulula*	-	-	1	1
Undet. Strigiformes (*Asio* / *Strix*)	1	-	-	1
Apodiformes				
*Tachymarptis melba*	2	1	1	4
**Galliformes**				
*Lagopus lagopus*	10	3	-	13
*Lagopus muta*	9	1	-	10
*Lagopus* sp.	11	6	-	17
Undet. Galliformes	13	22	-	35
**Gruiformes**				
cf. *Anthropoides virgo*	1	-	-	1
**Passeriformes**				
Undet. Alaudidae	1	1	-	2
*Turdus* sp.	-	1	-	1
*Sturnus* sp.	7	2	-	9
*Turdus* sp. / *Sturnus* sp.	8	5	-	13
*Corvus corax*	9	4	-	13
*Corvus* cf. *monedula*	1	-	-	1
Smaller corvidae	-	1	-	1
Passeriformes—Family, Genus and species unknown	12	4	-	16
Aves—Family, Genus and species unknown	6	7	-	13

### Gaviformes

**Fig 4:**
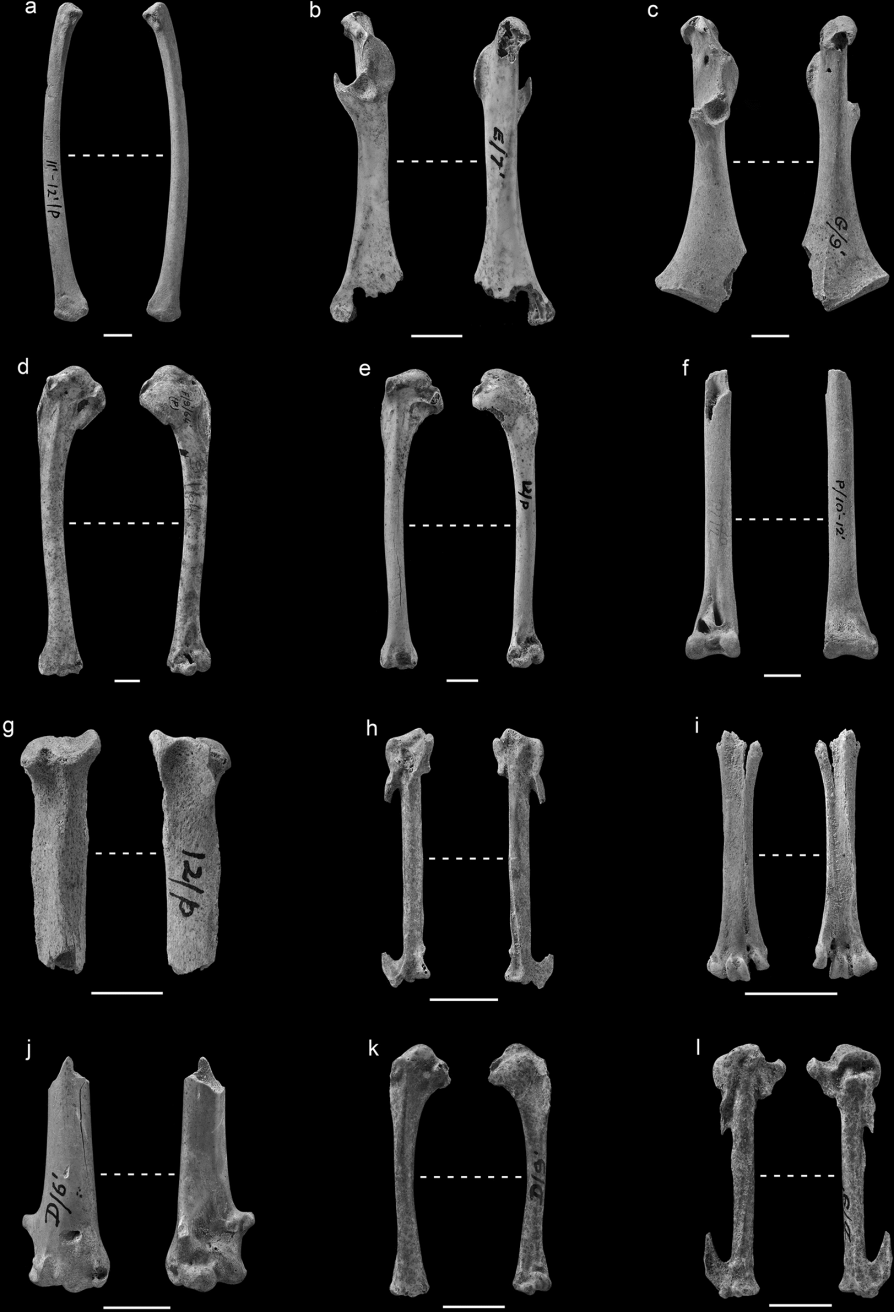
Representative skeletal elements of taxa in the MIS 3 deposits at Pin Hole. a); *Gavia* sp. left ulna PH(F) 18602; b) *Ardea* sp. right coracoid No PH(F) no.; c) *Ciconia* sp. right coracoid PH(F) 5596; d) *Branta bernicla* left humerus PH(F) 18543; e) *Melanita* sp. left humerus PH(F) 7805; f) *Buteo rufinus* distal left tibiotarsus No PH(F) no.; g) Large Falco sp. proximal left tibiotarsus PH(F) 8149; h) *Falco* cf. *tinnunculus* right carpometacarpus PH(F) 2614; i) small *Falco* sp. immature distal left carpometacarpus (PH(F) 9307); j) *Stercorarius* sp. distal left humerus (PH(F) 7629); k) *Charadrius morinella* left humerus PH(F) 7625; l) *Columba palumbus* right carpometacarpus PH(F) 2155;

An immature left ulna (PH(F) 18602) (Fig 4A), exhibiting a very distinctive dorso-ventral flattening is clearly that of a diver or loon *Gavia*. Identification beyond the level of genus is marred by the fact that the specimen belongs to an immature individual but according to the length the bone had attained at the time of death (GL 111.76) it would appear to belong to one of the smaller species, the red-throated diver *Gavia stellata* or less likely the black-throated diver *Gavia arctica* and considerably smaller than the great northern diver *G*. *immer* and the white-billed diver *G*. *adamsii*. Based on published measurements for the distal diagonal breadths of the ulnae of different *Gavia* species in North America [30] the present ulna (Did 12.4 mm) would appear to belong to that of the smallest diver *Gavia stellata* (Did 11.7–13.8, N = 15). *G*. *arctica* is larger (Did 13.0–16.4, N = 10) although the immaturity of the bone makes any comparison less reliable as it is not clear how much the breadth changes during development. Therefore, the specimen is identified as *Gavia* cf. *stellata* / *arctica*.

Divers, or loons, today breed close to fresh water in tundra and open moorland or in forested areas [45,46]. The fact that the specimen, attributed tentatively to a red-throated diver or black-throated diver, is immature may suggest that the species bred nearby and maybe even close to a water body in the Creswell Crags gorge itself.

### Ciconiformes

The grey heron *Ardea* cf. *cinerea* is represented by a right coracoid fragment (no PH(F) number) (Fig 4B). There is also a left humerus shaft (PH(F) 5895 A) which is from the upper level of Pin Hole. The coracoid is characteristic in having a wide open sulcus musculi supracoracoidei and a processus procoracoideus that projects medially to a great extent. The attribution of the humerus shaft to the heron family is based on the impression of the brachialis anticus which is distinctly long, thin and somewhat pointed unlike that in geese to which the specimen was formerly attributed. Table A in S1 File demonstrates that the humerus shaft is larger than that of all but the largest members of the Ardeidae in Europe *Ardea cinerea*. The grey heron today is found throughout much of temperate Europe, being absent from the areas north of southern Scandinavia. They are sedentary in Western Europe with migratory populations to the east. They usually nest in trees but they have rarely done so on cliffs and in reed beds. They feed in shallow fresh and coastal waters or in marshes [45,46].

A right coracoid (PH(F) 5896) (Fig 4C) can be attributed to the stork genus *Ciconia* sp. although its size (LM 73.94) falls in the distribution overlap of the two extant European species, the white stork *Ciconia ciconia* and the black stork *C*. *nigra* (Table B in S1 File). Some of the morphological characters used to distinguish the two species published [37] appear to suggest that the fossil belongs to *Ciconia ciconia* (such as the presence of a relatively distinct margo intermuscularis ventral) while others are of little use in this instance such as the relative length of the facies articularis sternalis because they are obscured by damage. The genus has been formerly recorded in Britain from Tornewton Cave where they were claimed to represent black storks *Ciconia nigra* [47]. Since that time the material in question has been relegated to *Ciconia* sp. because of the overlap in morphology between the two European species [48]. The habitat required by the two species is similar although the black stork nests in denser woodland. The white stork is almost commensal in Western Europe today nesting on buildings in areas of farmland although its natural nesting sites consist of trees. The white stork today is found in much of Western Europe in the breeding season and migrates south. The black stork’s distribution includes more eastern locations although it was present in Belgium until the 19^th^ century and recently became re-established in that country. It is also a migrant, moving to Africa and western Asia for the winter [49].

### Anseriformes

A complete left humerus (PH(F) 18543) (Fig 4D) is morphologically consistent with that of a goose Anserinae with features such as the prominent capital shaft ridge directed at the humeral head and the shape of the pectoral attachment on the external tuberosity which is somewhat circular and distinguish it from Anatinae, ducks [27]. The specimen’s size would appear to eliminate all but the smallest species, the brent goose and with some reservation is identified as *B*. cf. *bernicla* (Table C in S1 File). Table C in S1 File does reveal, however, that it is robust compared to the humeri of modern brent geese.

Brent geese today breed on the Arctic Sea shores and marshy tundra. They winter much further south on coastal mudflats and estuaries where they form flocks. This prompts the question whether the humerus is that of a breeding bird or a winter visitor. Sutherland [50] reviewed the Quaternary geese of Europe and concluded that instead of supporting a Flandrian (Holocene) Model of the origin of migration they suggested a new Pleistocene Inheritance Model. This meant that Sutherland considered that the goose distribution in the Late Pleistocene, with populations along the Mediterranean as well as further north (i.e. Pin Hole) suggested that the birds migrated between these areas. If the larger size of the brent goose humerus is caused by relatively sedentary lifestyle (see Discussion section ‘Size and shape differences in Late Pleistocene bird species’) this may signify less migration occurred at that time and so in this case would discredit the Pleistocene Inheritance Model for the evolution of migration, although this may seem unlikely.

A number of Anseriformes skeletal elements are identified merely as large Anseriformes without further refinement of identification due to their fragmentary state and the overlap in sizes of the smaller goose and large duck species. Therefore, the distal humerus and the three fragmentary coracoids in Table C in S1 File are simply identified as large Anseriformes. They include two right coracoids (PH(F) 5895 B) and (PH(F) 13083) attributable on size to small *Anser* or large *Branta* (white-fronted goose *A*. *albifrons*, pink-footed goose *A*. *brachyrhynchos* or barnacle goose *B*. *leucopsis*), a right coracoid (PH(F) 18540) attributable to *B*. *bernicla* or common shelduck *Tadorna tadorna* and a distal left humerus (PH(F) 13082) attributable to *B*. *bernicla*, *Tadorna tadorna* or common eider *Somateria mollisima*. They are not all likely to belong to a single species but cannot be identified further due to the overlap in size and shape of the Anseriformes in general. Finally, there is a large Anseriforme tarsometatarsus PH(F) 13065, previously and variously identified as shelduck and ruddy shelduck *Tadorna ferruginea*, which has trochlea that are widely spread and because the trochlea for digit II in internal view projects posteriorly the specimen is probably that of a goose (Anserini) rather than a shelduck (Tadordinidae) [27]. On size PH(F) 13065 is closest to that of a brent goose *B*. *bernicla* (PH(F) 13065: GL—57.97, SC—5.1; Modern *B*. *bernicla*: GL—58.5–67.2 (N = 10), SC—4.2–4.7 (N = 10) [28]). The discrepancy in measurements, and particularly its robust mid shaft measurement, are a cause for caution and so PH(F) 13065 is best considered a small goose based on the current evidence.

In addition there are a number of other elements, formerly identified as those of various geese, that are also identified here as large Anseriformes due to a lack of possible measurements, some of which could be large ducks or smaller geese. They include a left coracoid fragment (PH(F) 18542), a proximal right femur (PH(F) 7905), a furculum symphysis (PH(F) 2947), a distal left humerus (PH(F) 13082), a distal right humerus (PH(F) 8074), two anterior sternal articulations (PH(F) 13090 A) and (PH(F) 29197), a distal right tibiotarsus (PH(F) 13090 B) and a left scapula (PH(F) 18567).

The smaller Anseriformes, or Anatinae ducks, are also difficult to identify due to the number of species in the subfamily and their relatively conservative osteology. In order to help identify the duck remains the morphometric methodology that was developed and first used for the anatinae of West Runton [43] is applied to the material from Pin Hole. This methodology was developed to assess this category of remains even if it signifies that species identifications are not possible to assign. The method involves the use of size to ascertain the likely number of species present within the subfamily. A graphical technique has been employed. The technique transforms all the Pin Hole duck measurements to a percentage of an arbitrary mallard *Anas platyrhynchos*, a female (#MR40 in the author’s reference collection, the measurements of which are given in Text D in S1 File) (Fig A in S1 File). This demonstrates the size range of ducks present in the Middle Palaeolithic levels at Pin Hole. Also shown are the maxima and minima of the bone measurements of the mallard [28], expressed as percentages of the standard mallard. Finally, teal *A*. *crecca* maxima and minima from Woelfle [28] are plotted in order to give an idea of where the smallest duck in the Palaearctic today would fall. The Middle Palaeolithic Anatinae from Pin Hole plotted on this graph fall in the mallard range, the Eurasian teal range, and between the mallard and Eurasian teal ranges. No remains are smaller than that of modern Eurasian teal. This would suggest that the fossil duck bones from Pin Hole include at least three species of Anatinae.

In addition the most taxonomically diagnostic skeletal elements, the humerus and the tarsometarsus, were further investigated in relation to the anatomical characters identified as taxonomically diagnostic by Woolfenden [27]. However, for the fossils to be reliably identified it is essential that they be relatively complete as the relative bone proportions are the most clearly diagnostic characteristics. For example the three complete humeri (PH(F) 7805) and (PH(F) 7844) and (PH(F) 9074) clearly belong to at least two different species and are distinct in size (Fig A in S1 File). The smallest (PH(F) 9074) appears to be that of a dabbling duck because of the open pneumatic fossa with bone struts seen on the inside [27]. This small humerus was previously identified as that of a Eurasian teal *Anas* cf. *crecca*, which is consistent with its dimensions plotted in Fig A in S1 File, and is too short for that of the next smallest member of *Anas* in Europe the garganey *A*. *querquedula* [27], falling mostly within the modern range for the teal. The exception is the length which is a little longer than the modern range. The diagnosis of the specimen as a dabbling duck together with the size (Fig A in S1 File) would indicate that it probably represents a Eurasian teal *Anas* cf. *crecca*. Today Teal live in the subarctic to temperate zones and winter in warm temperate to Subtropical zones. They occur on small, shallow, slow moving freshwaters where they prefer thick emergent or marginal vegetation. The species will nest far from water in ground cover. They can be found on more open water bodies including shallow coastlines in winter [48].

The intermediate humerus (PH(F) 7844), is slightly larger than (PH(F) 9074), and was previously identified as that of a tufted duck *Aythya fuligula* by D. Bramwell and a long-tailed duck *Clangula hyemalis* by C.J.O. Harrison. Based on Fig A in S1 File the specimen would appear in size to be neither a Eurasian teal nor a mallard. The morphology of this specimen, with a closed pneumatic fossa at the proximal end is consistent with the specimen belonging to either a member of the genus *Aythya*, an eider (*Somateria* sp.), a sea duck (*Melanita*, *Histrionicus*, *Clangula*, *Bucephala*) or a stiff tail (*Oxyura*) [27]. The length of the humerus [28], would appear to eliminate all but the Smew *Mergus albellus*, the goldeneye *Bucephala clangula* and the long-tailed duck *Clangula hyemalis*, although in *Mergus* the morphology is more like that in *Anas* (with an open pneumatic fossa). So the specimen would appear to represent either a goldeneye or a long-tailed duck. Goldeneye breed today in the boreal to cool temperate zone and winter in the boreal to warm temperate zone. It nests in holes in trees near freshwater and when wintering is found on estuaries or sheltered coastlines or even open freshwater. The long-tailed duck today breeds in Arctic to Subarctic zones wintering in warmer Arctic to boreal zones. They breed on offshore islands or coastal tundra and if freshwater is present in inland tundra or Alpine shrub tundra. They are found in coastal areas in winter and occasionally in freshwaters [49].

The largest complete humerus (PH(F) 7805) (Fig 4E) is gracile, compared to that of a mallard (Fig A in S1 File). It has a similar proximal breadth to that of a mallard but has a very gracile distal end and shaft. It also has a deltoid crest that extends greatly in a distal direction and flares distally which is consistent with the morphology of a scoter *Melanitta* humerus [27]. This humerus was previously identified as common scoter *Melanitta nigra* and certainly the humeral length, distal and shaft breadths fall within the range of its measurements although the proximal breadth is too large (Table D in S1 File). Conversely, the length and proximal breadths fall in the range of the velvet scoter *M*. *fusca* while their distal and shaft breadths are too small for *M*. *fusca*. This specimen has therefore been identified as unspecified scoter *Melanitta* sp.

A left tarsometatarsus (PH(F) 13084), however, was previously identified as a common scoter *Melanitta nigra* and is cautiously confirmed here (*M*. cf. *nigra*). The genus is diagnosed due to the characteristically robust dimensions of the tarsometatarsus [27]. The species is derived from its relative length, as the breadth measurements fall within the overlap range of both European *Melanitta* species (Table D in S1 File). The common scoter breeds today on tundra by freshwater and usually winters in large flocks in marine waters. It seems likely, given the inland location of Pin Hole, that the bird represented by this element was a breeding individual. Velvet scoter has a very similar distribution today (Arctic to boreal zones—Summer, coastal boreal zone—Winter) also breeding near inland freshwater although often in more forested habitats than the common scoter. Like the common scoter it winters offshore although the velvet scoter is more likely to be found today near-shore among rocks and islands [49].

A final relatively complete tarsometatarsus (PH(F) 7591) was previously identified as *Lagopus lagopus*. PH(F) 7591 looks like a member of *Anas* and according to Fig A in S1 File is consistent with a small mallard *A*. *platyrhynchos* but because of the overlap with other members of the Anatinae it is best to consider this specimen as a medium-sized duck (Anatinae).

### Accipitriformes

An immature distal left tibiotarsus (No PH no.) (Fig 4F) belongs to that of an Accipitriforme rather than that of a Falconiforme due to the less complex morphology of the pons supratentineus with three apertures in the Falconiformes not seen in the present specimen. It is closest in size to that of modern long-legged buzzard *Buteo rufinus* and goshawk *Accipiter gentilis*. However, the distal articulation, when viewed distally, is wider medio-laterally in relative terms in *Buteo* than in *Accipiter*. This was confirmed with measurements and the specimen would appear to belong to that of a long-legged buzzard [30] (Fig B in S1 File). The species has not been found in the fossil record of Britain before now, though there are records from the Late Pleistocene of France and Luxembourg [51,14] and is therefore not wholly unexpected. The long-legged buzzard lives today in North Africa and Eastern Europe [45]. The species occurs in open plains, the steppes, semi-deserts and mountains in the breeding season and is sedentary [51].

### Falconiformes

A large falcon *Falco* sp. is represented by a proximal left tibiotarsus (PH(F) 8149) (Fig 4G). The specimen had previously been identified as that of a rough-legged buzzard *Buteo lagopus*. A morphological comparison between the specimen and those of buzzards as well as other Accipitriformes and large falcon tibiotarsi suggests that the specimen belongs to a member of the genus *Falco*. The shape of the facies gastrocnemialis is more rounded in *Falco* and the crista cnemialis cranialis projects proximally to a lesser extent in *Falco* than in Accipitriformes. The species is difficult to identify to species reliably as the specimen is damaged and the measurements taken by Solti [32] cannot be taken on the present specimen. It would appear that the specimen approximates the size of the lanner *Falco biarmicus*, saker *F*. *cherrug* and peregrine *F*. *peregrinus* falcons.

Five remains of small falcons were found in the Middle Palaeolithic level including a right carpometacarpus (PH(F) 2614) (Fig 4H), a left humerus (PH(F) 18548), a distal right tibiotarsus PH(F) 18545), an immature left tibiotarsus (PH(F) 8269) and an immature distal left tarsometatarsus (PH(F) 9307) (Fig 4I). The identities of these falcon bones are not easily diagnosed because there is some overlap in the dimensions of the bones of the smaller Western Palaearctic falcons today [33]. The carpometacarpus (PH(F) 2614) is consistent in length with that of the hobby *F*. *subbuteo* and kestrel *F*. *tinnunculus* today, if data from Solti [33] is included in the comparison, and too long for that of a merlin *F*. *columbarius* (Fig C in S1 File; Table E in S1 File). Its breadth dimensions are however relatively slender for its length compared to that of a hobby *F*. *subbuteo* (Fig C in S1 File; Table E in S1 File). The Pin Hole carpometacarpus is large compared to that in the red-footed falcon *F*. *vespertinus* and the lesser kestrel *F*. *naumanni* today (Fig C in S1 File; Table E in S1 File). The specimen is therefore tentatively identified as that of a kestrel *F*. cf. *tinnunculus*. The other specimens, the left humerus (PH(F) 18548), distal right tibiotarsus (PH(F) 18545), immature left tibiotarsus (PH(F) 8269) and immature distal left tarsometatarsus (PH(F) 9307) are more difficult to assign to species because they fall in the overlap range of more than one species of modern Western Palaearctic falcon which generally includes the kestrel and are therefore merely identified as small *Falco* sp.

### Charadriiformes

The second record of a skua for the Pleistocene of the British Isles is represented by a distal left humerus (PH(F) 7629) (Fig 4J). The specimen was formerly identified as that of a common gull *Larus canus* [17]. Comparison between the present specimen and a comparative common gull humerus reveals that it is unlikely to belong to a gull because of the degree of excavation (depth) of the fossa musculi brachialis of the fossil which is too shallow. In gulls this fossa is deeper. The specimen is not that of a large tern as the processus supracondylaris dorsalis is much more extensive in a proximal direction and generally larger in *Sterna* than in the fossil. The latter is a characteristic of tern humeri. Finally, the specimen appears to be that of *Stercorarius* rather than that of the large *Numenius* waders due to the less rounded proximal edge of the condylus dorsalis when viewed cranially and the more rounded profile of the ridge ventral to the sulcus musculi scapulotricipitis when viewed distally. The reliability of the second *Stercorarius* character described is that it is present in 94% (N = 34) of modern skua humeri while it is present in 5.6% (N = 18) of modern *Numenius* humeri. Therefore, the specimen appears to be that of a skua. Measurements taken on the specimen and plotted in Fig D in S1 File with those of modern comparative skua humeri suggest that the specimen belongs to either the Arctic skua *Stercorarius parasiticus* or the Long-tailed skua *S*. *longicaudatus* plotting between modern measurements of the two species. The other record of skua in Britain from the Pleistocene is of a long-tailed skua from Soldier’s Hole in Somerset from a deposit thought to date from 50–30,000 BP [52,14] and so is approximately the same age.

The Pin Hole skua specimen has small holes that seem to indicate small mammalian carnivore activity, predation or scavenging. The two potential skuas represented have similar ecological requirements, breeding on barren moorlands and tundra, nesting on the ground, passing along coastlines on passage and spending the winter at sea. They are very rarely seen inland outside the breeding season [45]. The diet of the long-tailed and Arctic skuas during the breeding season includes rodents such as lemmings and voles, particularly when these mammals are common [53,54].

A right coracoid fragment (PH(F) 8146) consisting of the scapular articular end was previously identified as that of a black guillemot *Cepphus grille* by Bramwell [17] and puffin *Fratercula arctica* by Colin Harrison (Unpublished notes). The specimen has no secure stratigraphic provenance but is included in the description because it is an unexpected find for a site at such a distance (>100 Km) from the sea. Comparisons made with the range of possible alcid coracoids in this study suggest that the specimen is difficult to identify to species with confidence due to its fragmentary nature. Guillemots *Uria* sp. and black guillemots *Cepphus grylle* can be eliminated as possible identities because they are too large and because the shape of the acrocoracoid process in those taxa is different to that in *Alca* and *Fratercula* when viewed from the top of the shoulder extremity. Therefore, the specimen would appear to belong to either that of *Alca torda* or *Fratercula arctica*. The razorbill varies between being a dispersive and migratory species depending on whether it lives under the influence of the North Atlantic Drift. It breeds in the Arctic to cooler temperate areas and wintering as far south as the Mediterranean [49]. The Puffin is similar although it has less of a tendency of being resident in the north during the winter. They both breed colonially although the razorbill breeds on rock faces and amongst rocks while the puffin breeds mainly in burrows and rock crevices. The distance from the coast for such a marine bird may be explained by the phenomenon particularly known in the little auk *Alle alle* as “wrecking”, the phenomenon whereby numbers of sea-birds, usually little auks, but also other auk species, are blown inland by storms [55,56].

The two left humeri, (PH(F) 7625) (Fig 4K) and (PH(F) 12575), can be seen to belong to members of the Charadriiformes due to the presence of a pronounced processus supracondilaris dorsalis and because of their sizes they most closely conform to those of the ruff *Philomachus pugnax*, the knot *Calidris canutus* and the redshank *Tringa tetanus* within the family Scolopacidae. There were, however, characters that allowed the elimination of the Scolopacidae suggesting that the identification lay amongst the Charadriidae. These included the more proximally extended processus supracondilaris dorsalis in Scolopacidae than in Charadriidae (broken off in PH(F) 7625 but visible in PH(F) 12575), and the presence of a pronounced ridge along the proximal margin of the crista pectoralis in *Charadrius* which was not seen in either *Philomachus* or *Calidris*. In *Philomachus*, *Tringa* and *Calidris* the muscle attachment area which is presumably the tuberculum dorsale runs distally into the crista pectoralis parallel with the shaft. Of the Charadriidae the most similar in size appears to be the modern dotterel *Charadrius morinella*. PH(F) 7625 and PH(F) 12575 respectively GL = 43.26 and 40.3 and the reference material ranges between GL = 39.3–42.52 (N = 4). The dotterel today breeds in open tundra areas in the Arctic zone and similar areas in the dispersed Alpine zone of Eurasia and winters in the warm temperate zone [49]. The breeding distribution has been used to suggest a formerly more continuous range over more lowland Europe during glacial times [49] and the present record is presumably confirmation of this.

In addition there is distal right tibiotarsus (PH(F) 8082) and a proximal left femur (PH(F) 9322) both of which appear to belong to small waders in the family Scolopacidae. The former because it is too robust for a member of the Charadriidae, and the latter because the proximal morphology (the shape of the crista trochanteris) is distinct from that in the Charadriidae. The bones appear to belong to small Scolopacidae species based on their greater robusticity than those of Charadriidae, although their fragmentary and worn states prevent further identification. Finally, there is a right carpometacarpus (PH(F) 18547) that belongs to a Charadriiforme, possibly a wader the size of a modern member of *Vanellus vanellus*, although too badly damaged to confirm.

### Columbiformes

The right carpometacarpus (PH(F) 2155) (Fig 4l) from Pin Hole appears to belong to a wood pigeon *Columba* cf. *palumbus* and not to other members of the genus *Columba* found in Europe today (*C*. *livia* and *C*. *oenas*) although in a number of measurements, including the greatest length, it falls a little above the ranges quoted by Fick [40] (Table F in S1 File). This discrepancy is difficult to interpret as there exists only one specimen from Pin Hole, although it is interesting to note that other bird species during the Late Pleistocene have larger individuals [57,39]. The possibility that the specimen belonged to a sandgrouse exists because of the occurrence of steppic taxa in the Pin Hole assemblage. Also these birds have been identified in the Late Pleistocene of Europe [41] although mostly in the Mediterranean [14]. The specimen does not, however, belong to that of a sandgrouse Pteroclidae because the carpometacarpus in Palaearctic *Pterocles* and *Syrrhaptes* are all significantly smaller (Table F in S1 File). The proximal morphology of the carpometacarpi in the two genera of sandgrouse are characterised as having an articulation which is less extensive relative to those in the Columbidae. The distal carpometacarpus differs in the Pteroclidae in having a less pronounced angle where the os metacarpale minus joins the os metacarpale majus. The woodpigeon today is found in much of Europe except the furthest north. They are resident to partially migratory to the south and west and migratory the further east and north. They nest in trees and have been recorded nesting on the ground.

### Strigiformes

**Fig 5:**
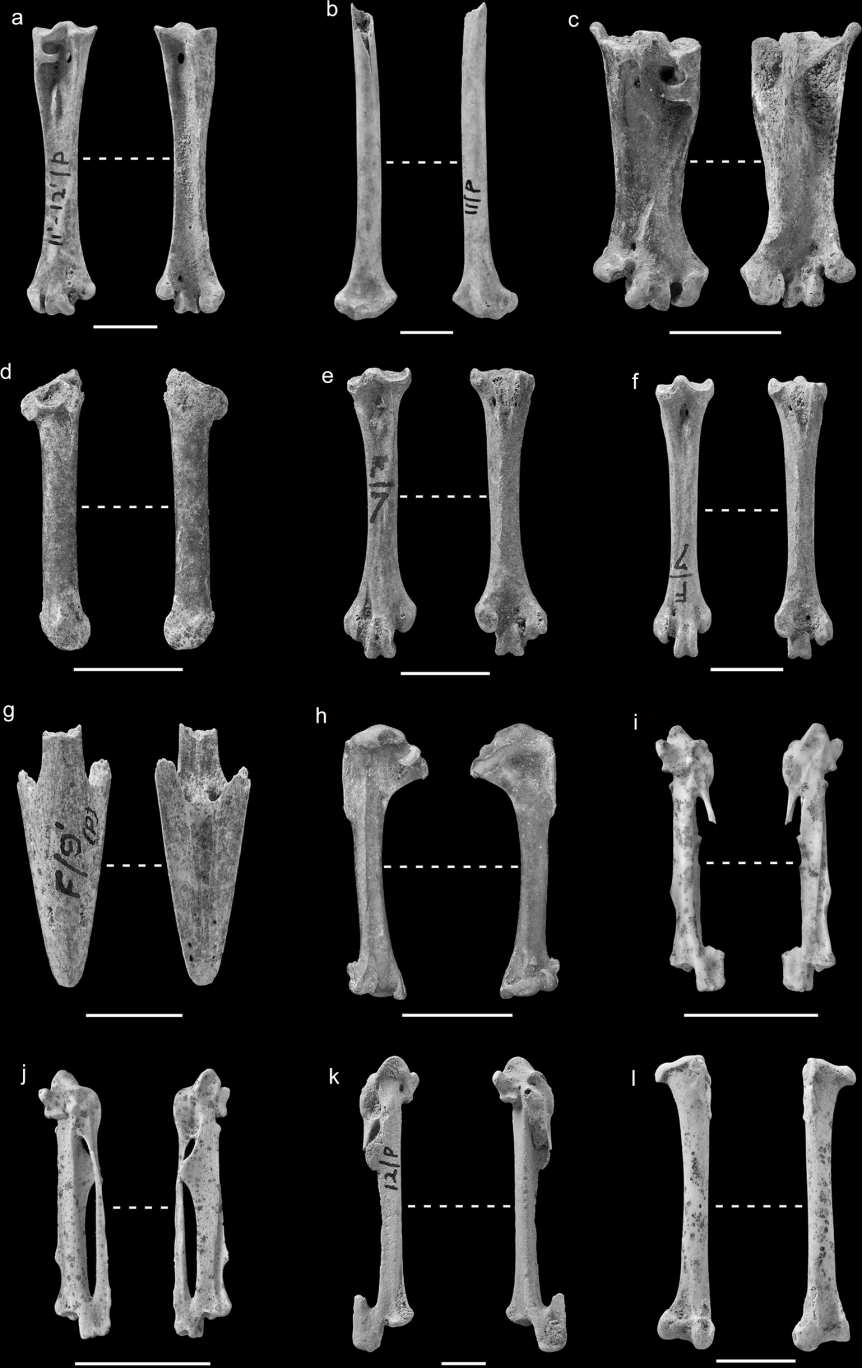
Further representative skeletal elements of taxa in the MIS 3 deposits at Pin Hole. a) *Asio flammeus* left tarsometatarsus PH(F) 7837/38; b) *Bubo* sp. distal left radius PH(F) 7801/00; c) *Surnia ulula* right tarsometatarsus PH(F) 8476; d) *Tachymarptis melba* right ulna PH(F) 18600; e) *Lagopus muta* right tarsometarsus PH(F) 9; f) *Lagopus lagopus* right tarsometarsus PH(F) 266; g) *Anthropoides virgo* premaxilla PH(F) 30; h) Alaudidae left humerus PH(F) 8064–8068; i) *Turdus* sp. right carpometacarpus PH(F) 9069–9071; j) *Sturnus* sp. right carpometacarpus PH(F) 7374; k) *Corvus corax* right carpometacarpus PH(F) 13075–13076; l) *Corvus* cf. *monedula* right femur PH(F) 8069.

Two owl tarsometatarsi, both with the same undifferentiated code (PH(F) 7837 and 7838) (Fig 5A), from Pin Hole were previously identified as those of the short-eared owl *Asio* cf. *flammeus*. Table G in S1 File, using data from Langer [35] confirms this identification. The measurements of *Asio flameus* are similar to those of the tawny owl *Strix aluco*, however, the non-metric morphology of the specimens seems to suggest that the fossils are more like the tarsometatarsi of *Asio flammeus* than those of *Strix aluco* due to a presence of a shorter, proximo-distally, crista medialis hypotarsi and a more asymmetrical trochlea metatarsi tertii when viewed from the distal end looking proximally.

Short-eared owls today breed on boggy moorland, upland pasture, young conifer plantations and marshes; they winter in open moorland, grassland and marshes. In Britain, they are a widespread breeding bird of open country, mainly in Scotland and northern England, a few pairs also breed on saltmarshes on the east coast. They are more numerous in winter, when they are most common on the east and south coasts of England. They are diurnal and therefore more easily seen than most owls. Widespread in northern Europe in summer, occurring in much of southern Europe in winter where coastal marshes and meadows (e.g. in Holland) are particularly favoured. They nest on the ground and feed on a range of vertebrates, particularly voles [58].

A large owl, of the genus *Bubo* is represented by a distal left radius (PH(F) 7801 or 7800) (Fig 5Bm), and possibly by a fragmentary right carpometacarpus, constituting the distal part of the os metacarpale majus (PH(F) 8200). Both are too fragmentary for confident attribution to either species of *Bubo* found in the region today, the eagle owl *Bubo bubo* or snowy owl *B*. *scandiaca*. The other large owls in the region today the Ural owl *Strix uralensis* and the great grey owl *S*. *nebulosa* are too small for either specimen. However, on visual inspection the size of the fragmentary carpometacarpus appears to be too small for the nominate subspecies *Bubo bubo bubo* signifying that its identity lies with *B*. *scandiaca*. However, caution needs to be exercised as here there are eagle owls that are distinctly smaller than the northern *Bubo bubo bubo* that live in desert areas such as North Africa although they are now considered to be the separate species *Bubo ascalaphus desertorum* [59]. Both species *B*. *bubo* and *B*. *scandiaca* are known from the British Quaternary record [59]. *B*. *bubo* is today a resident species in Subarctic to Subtropical zones. It occurs in all rocky or wooded areas from low lying parts to mountains, with vegetation from bare ground with small numbers of trees through grasslands to dense forests. It nests in rock cavities or old nests in trees [49]. Meanwhile, the snowy owl *B*. *scandiaca* is found in the Arctic zone into the Alpine zone in Scandinavia today. It nests on the ground, occurs in dry tundra and on rocky offshore islands, and is resident although will irrupt southwards under certain harsh conditions [49].

There is also a fragmentary left coracoid (PH(F) 1341) that can be identified as a medium sized owl in the genus *Strix* or *Asio*. *Tyto* can be eliminated as a possible identity due to the morphology. In *Tyto* the facies dorsalis is broader than in either *Asio* and *Strix*. The specimen had previously been identified as carrion crow (*Corvus corone*) although because of the presence of a foramen in the procoracoid (coracoideal fenestra) this can be discounted.

Finally, there is the complete right tarsometatarsus (PH(F) 8476) (Fig 5C) of a northern hawk owl *Surnia ulula* which, although it was not found in the MP levels, is a notable sole British record and is identified based on its very short length with robust breadth. The specimen was found between 3 and 6 feet depth, between 50 and 60 feet into the cave suggesting that it belongs to the upper fauna. The measurements of *Surnia ulula* reference material (GL = 24.02–25.96 (N = 13), SC = 5.2–6.02 (N = 13)) eliminate all other, similarly shaped, Strigiformes in the Western Palaearctic such as Tengmalm’s Owl *Aegolius funereus* ((PH(F) 8476 GL = 24.72, SC = 5.88) and *Aegolius funereus* (GL = 23.14–22.12 (N = 2), SC = 3.36–4.02 (N = 2)). Today, the northern hawk owl lives in the boreal forest region of the Palaearctic and Nearctic and usually stays within its breeding range, though it sometimes irrupts southward [49].

### Apodiformes

**Fig 6:**
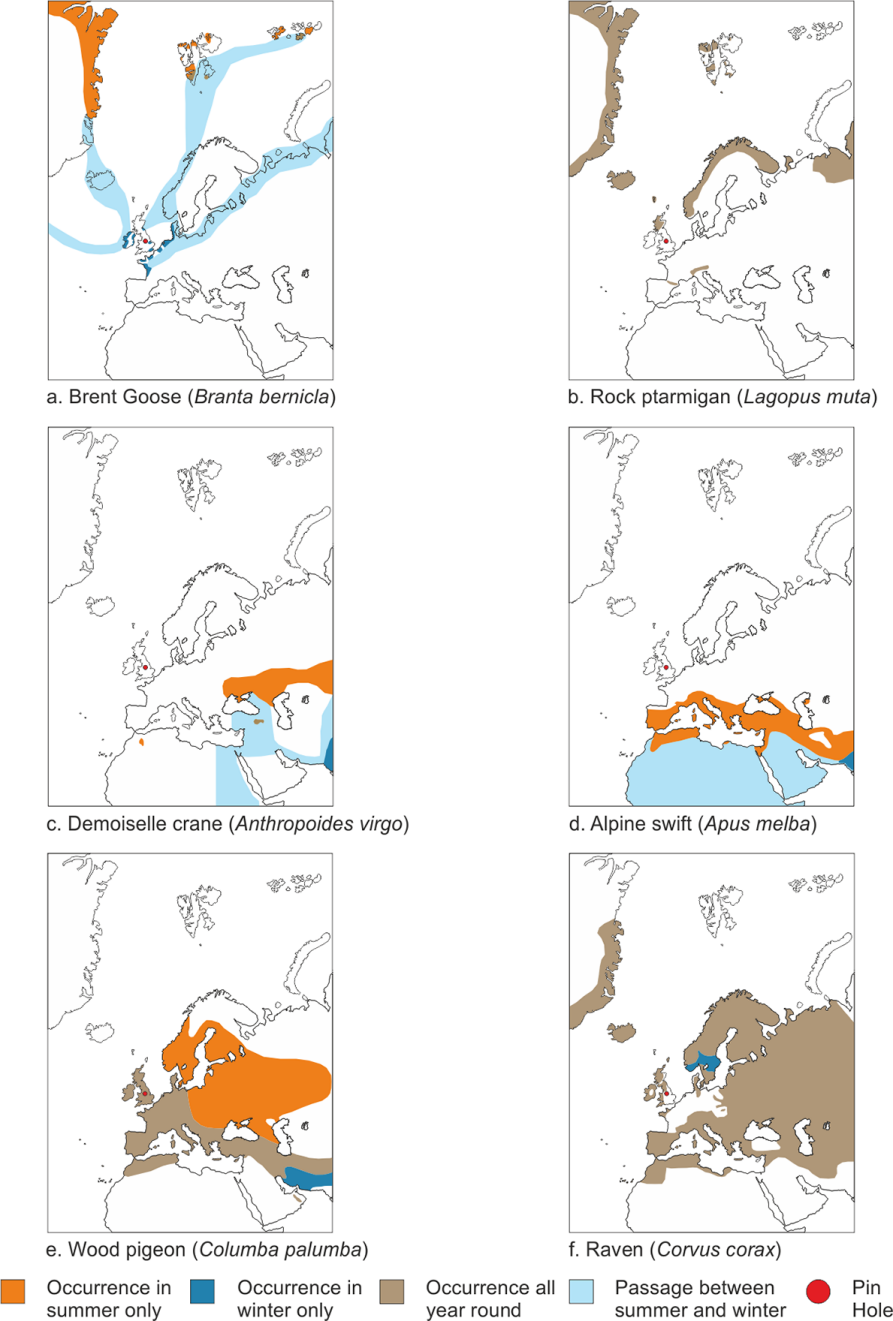
Modern geographic range of taxa found at Pin Hole. a. Brent goose *Branta bernicla*; b. Rock ptarmigan *Lagopus muta*; c. Demoiselle crane *Anthropoides virgo* d. Alpine swift *Tachymarptis melba*; e. Wood pigeon *Columba palumba*; f. Raven *Corvus corax*. Note that these maps are taken from Harrison [49] and that distributions may have changed since 1982 when they were originally published. This is certainly true of the raven whose range has expanded into the gap in the range in North West Europe since then.

Swift bones are some of the most distinctive avian bones due to their extreme adaptation to flight. There are four swift bones, a right tarsometatarsus, a right carpometacarpus and a left distal femur in the MIS 3 levels at Pin Hole as well as a right ulna which is unprovenanced (Fig 5D). They are large and correspond in size with that of the Alpine swift *T*. *submelba*, the largest European species. Fig E in S1 File demonstrates that the ulna is large and falls outside the breadth range of the two modern Alpine swifts available as well as the fossil remains attributed to the species from Devil’s Tower in Gibraltar. Jánossy [60] described large swift material of Middle Pleistocene *age as Tachymarptis* (formerly *Apus*) *submelba*, distinguishing it from *T*. *melba* as having longer, and particularly more robust, bones. It may be that the material from Pin Hole should be attributed to this species although it may be that *T*. *submelba* is a large form of *T*. *melba* and may not merit separate species status (see [61]). The Alpine swift today lives in the circum-Mediterranean area (Fig 6D), in rocky arid regions from sea level to the high altitudes of mountains [41].

### Galliformes

**Fig 7:**
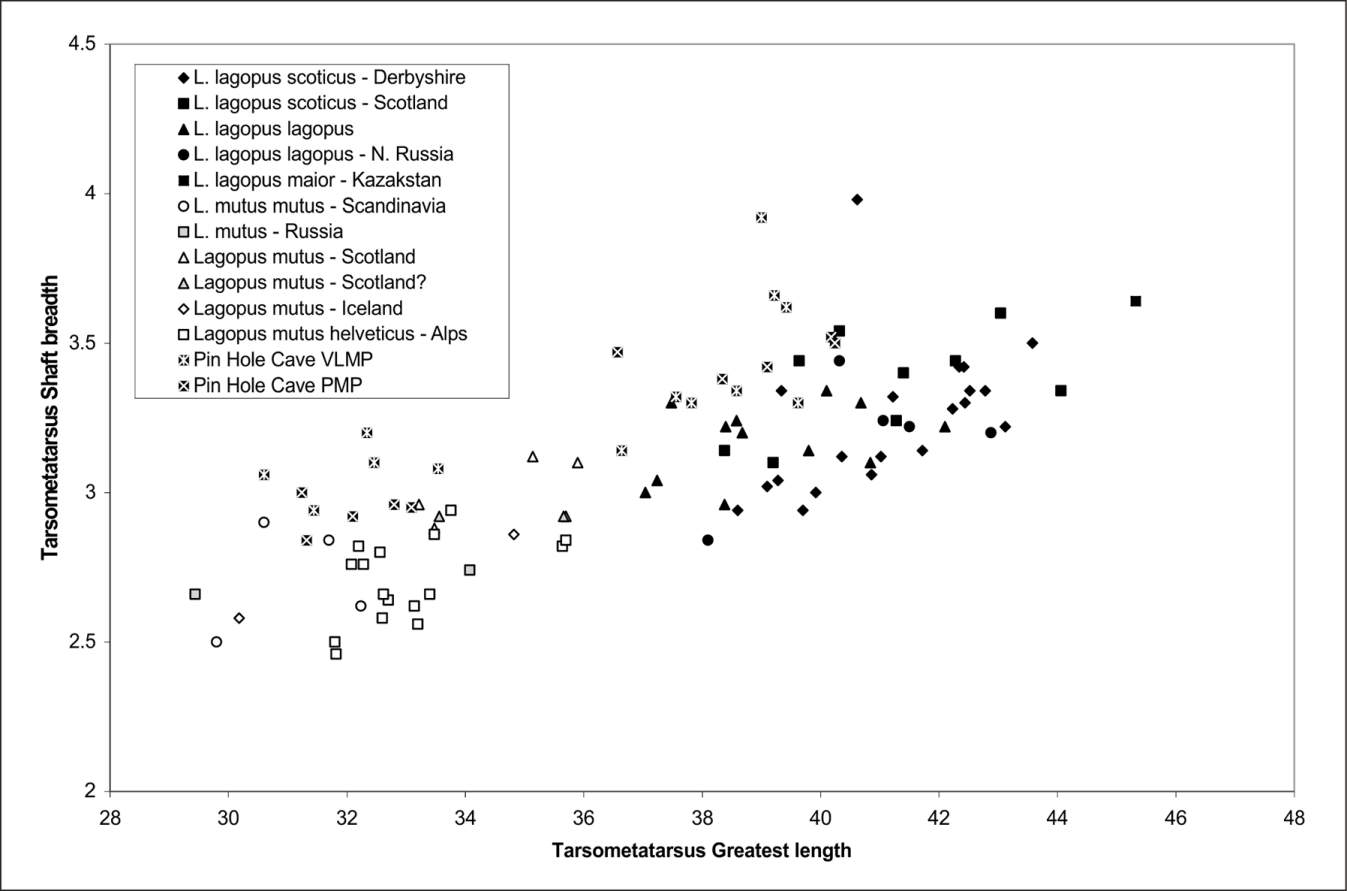
Scattergram with tarsometatarsus greatest length measurement versus shaft breadth of *Lagopus lagopus* and *L*. *muta* from Middle Palaeolithic of Pin Hole Cave together with those of modern comparative specimens of both species.

The most numerous bird remains in Pin Hole, and arguably in the entire European Pleistocene fossil record, belong to Galliformes in the genus *Lagopus* [62]. Of those the genus *Lagopus* is the best represented by both European species *L*. *muta* (Fig 5E) and *L*. *lagopus* (Fig 5F). The distinction between *Lagopus lagopus* the willow ptarmigan / red grouse and *Lagopus muta* the rock ptarmigan is best made using the tarsometatarsus and the humerus [38]. Based on these elements 13 specimens of *L*. *lagopus*, and 10 specimens of *L*. *muta* have been identified. In addition there were 16 specimens attributed to *Lagopus* sp. and a further 31 to undetermined medium sized Galliformes (see S1 File). The specimens identified to species were measured and these measurements together with metrical data for modern populations were plotted in a bivariate scatter to both confirm the identities as well as demonstrate that the tarsometatarsi have greater shaft breadths (Fig 7). The latter has been shown in former studies of European *Lagopus* [63,51], including ones on the Pin Hole material [59,38]. The humeri from Pin Hole have also been shown to have dimensions that differ from modern counterparts of the two species in having broader proximal ends [57,38]. The significance of the Pleistocene *Lagopus* species with greater tarsometatarsus shaft widths is probably due to greater bird weights which in turn may be due to the higher carrying capacity of the environment of the mid-latitudes of Europe at this time [57,38]. The different dimensions of the humeri may also be related to the birds greater weight in the past although it could be that they were more mobile. The latter, because it may be related to greater development of flight muscles, could also contribute to the birds greater weight [57,38]. It is also possible that these populations are now extinct having not contributed to modern European populations and may not have habitat tracked during the population contraction phase [64,65].

Today, the rock ptarmigan *Lagopus muta* lives in the northern reaches and mountainous areas of the Palaearctic and Nearctic in tundra and treeless moorlands and is a sedentary species changing plumage colour to a cryptic white in winter [66]. Meanwhile, the willow ptarmigan / red grouse *Lagopus lagopus* is similar although it generally lives at lower altitudes to the rock ptarmigan and is variable in the degree to which is has a white winter plumage phase [66].

### Gruiformes

A premaxilla (PH(F) 30) (Fig 5G) was identified as that of a demoiselle crane *Anthropoides virgo* by Cowles [67] but was subsequently thought to be lost. Recently the specimen was relocated in the collection of the Creswell Crags Museum and Education Centre, Nottinghamshire. A re-examination confirms that the specimen is likely to be that of a demoiselle crane although it differs from eight specimens of that species in being distinctly broader in relation to its length (Fig F in S1 File). The occurrence of this crane in Britain at some distance from its current distribution is of interest, and due to its distribution today in the steppe zone, would appear to be further evidence of this biotope in the north-west of Europe during early MIS 3. The species today lives in drier and more open areas than the common crane *Grus grus*, breeding in marshes and marshy river valleys, grassy plains and mixed grass / shrub steppes [49].

### Passeriformes

Two left humeri, (PH(F) 8064 to 8068) (Fig 5H) and (PH(F) 9323) appear to be those of a lark and were previously attributed to skylark *Alauda arvensis*. Jánossy [68] described characters to distinguish the humeri of larks from those of other passerines although he warned about difficulties of mistaking them for shrikes *Lanius*, orioles *Oriolus* and waxwings *Bombicilla*. Therefore, the specimens were compared to specimens of those genera as well as various Western Palaearctic lark genera and species (*Lullula*, *Eremophila*, *Alauda*, *Galerida*, *Calandrella* and *Melanocorypha*). The orioles could be eliminated on the basis that their humeri are considerably more robust and larger with a deeper pneumatic fossa, and a lack of excavation below the caput and the crista pectoralis that projects more cranially. The waxwings *Bombicilla* are similarly too robust for the Pin Hole specimens and their crista pectoralis is shorter and has an angle towards the proximal end, more like that in *Sturnus*. Finally, in the shrikes the edge of the crista pectoralis is rounded compared to the more proximo-distally elongated and cranially more flattened shape in Alaudidae. The dimensions of the lark humeri as seen in Fig G in S1 File demonstrate that they most likely belong to one or more of the following: skylark *Alauda arvensis*, crested lark *Galerida cristata*, Theckla lark *G*. *theklae*, and shore lark *Eremophila alpestris*. Larks are generally indicative of open habitats, living and nesting on the ground, and feeding on a mixture of seeds, plants and insects [49].

There is a carpometacarpus that can be identified as belonging to members of the genus *Turdus* due to the presence of a discontinuity in the articular facet of the facies articularis ulnocarpalis of the specimens. Measurements indicate that the specimen’s identification to species is difficult to confirm with certainty [34] (Table H in S1 File). PH(F) 9069 to 9071 (Fig 5I) could be ascribed to that of a blackbird *T*. *merula* and is just outside the upper part of the size range of the song thrush *T*. *philomelos* and the redwing *T*. *iliacus*. However, due to the overlap in measurements between the *Turdus* species in Britain today, the fact that no metrical comparison has been made with material of the different *Turdus* taxa beyond Britain to establish the size variation in the genus and within the species and because it is likely that the species varied in the Pleistocene, the specimens have not been identified beyond the level of genus. The problems inherent in identifying members of the genus *Turdus* have been discussed in detail elsewhere [61,69] as there is a clear tendency to consider only the species living in the general area from which the fossils were found. In the case of Pin Hole this would generally involve the six British members of the genus the blackbird *T*. *merula*, the song thrush *T*. *philomelos*, the missle thrush *T*. *viscivorus*, the ring ouzel *T*. *torquatus*, the redwing *T*. *iliacus* and the fieldfare *T*. *pilaris*. This maybe a problem because there are others in the western Palaearctic that are rarely considered such as the rock thrush *Monticolla saxatillis* and the blue rock thrush *M*. *solitaries* from southern Europe, and the eastern thrushes such as Siberian thrush *T*. *sibiricus*, the eye-browed thrush *T*. *obscurus*, Naumann’s thrush *T*. *naumanni*, the black-throated thrush *T*. *ruficollis* and White’s thrush *Zoothera dauma* [69]. The latter possibilities, which live in areas to the east of Europe are important to consider because Pin Hole has other similarly exotic taxa whose identities are unquestionable. Unfortunately, the available comparative material for the eastern thrushes is poor and so they cannot be reliably considered. This is another justification for leaving the identity of the *Turdus* remains at the level of genus. Members of the genus *Turdus* are found in many different types of habitats that include trees or scrub, in which they nest. They live in the Arctic / Alpine to warm temperate zones and are partially or fully migratory and their diet consists of invertebrates.

There are a number of specimens that can be identified as belonging to starlings *Sturnus* sp. although it is difficult to confidently ascribe them to species. The five carpometacarpi (Ph(F) 7372, 7373, 7374, 7375 and 7376) (Fig 5J) have been identified as belonging to *Sturnus* rather than *Turdus*, because they have no discontinuity in the articular facet of the facies articularis ulnocarpalis (no discontinuity in 87.18% *Sturnus* and present in 87.06% *Turdus*) [38]. The complete left humerus and the proximal right humerus (both PH(F) 9069 to 9071) as well as the proximal left humerus (Ph(F) 7370) can also be attributed to *Sturnus* due to their possessing a pronounced angle in the crista pectoralis unlike that in specimens of *Turdus* [34,38]. All these starling remains had previously been identified as those of members of the genus *Turdus* illustrating the difficulty that exists distinguishing these taxa.

Fig H in S1 File demonstrates that the carpometacarpi are within the range of measurements for those elements of common starling *S*. *vulgaris* and rose-coloured starling *Pastor roseus*. If they belong to the common starling, they are within the size range of individuals in Britain today and being relatively small may well be relatively migratory, although this is speculative [38,70]. Common starlings *Sturnus vulgaris* live today on grassland, particularly if it has been grazed and will also eat ectoparasites off the backs of large herbivorous mammals. They are particularly adapted to feeding in the turf by prying with their bills for insect larvae such as the leatherjacket, the cranefly larvae [70]. The rose-coloured starling *Pastor roseus* is also found in open habitats and is particularly fond of locusts and often accompany them during “plagues” [70]. The spotless starling *Sturnus unicolor*, not considered here a likely candidate for identification as it is too large, is similar but with a particular fondness for dung beetles [70].

The distinction of many elements of *Turdus* and *Sturnus* may not be possible and the following elements have not been identified beyond a category which includes both taxa (*Turdus* sp. / *Sturnus* sp.): Right ulna (No Ph number), right coracoid (PH(F) 8054), proximal left tarsometatarsus (PH(F) 9327–9330), left tarsometatarsus (PH(F) 8070), left tarsometatarsus (PH(F) 403), left tarsometatarsus (PH(F) 140), proximal left tarsometatarsus (PH(F)1098), distal left tibiotarsus (PH(F) 501), distal right tibiotarsus (PH(F) 7371), right ulna (PH(F) 9053 or PH(F) 9054 or PH(F) 9055 (18i)).

Five raven bones (a premaxilla (PH(F) 1044), a distal left ulna, a distal right radius, an immature left tibiotarsus, a distal left tibiotarsus and a distal left tarsometatarsus (PH(F) 1119 or PH(F) 6) come from the Middle Palaeolithic levels at Pin Hole and a further seven possibly emanate from that deposit (a proximal left ulna (No PH No), a right carpometacarpus (PH(F) 13075 to PH(F) 13076) (Fig 5K), a distal tibiotarsus (PH(F) 13075 to PH(F) 13076), a distal left carpometacarpus (No PH no.), a distal left femur (No PH number), an immature distal right humerus (No PH no.) and a right proximal coracoid (Ph(F) 7840)) [38].

The identification of these remains is relatively easily achieved as they are clearly those of a passerine and the raven is the largest member of that order in Europe. The sizes of the raven bones from Pin Hole are consistent with those of the population in Britain today, although the tarsometatarsi are relatively large and fall just outside the modern British sample [38] (Figs I and J in S1 File). The British birds today belong to the nominate subspecies *Corvus corax corax*, are larger than the Iberian subspecies *C*. *corax hispanu*s and smaller than members of the nominate subspecies from Scandinavia [38]. In fact it appears that the raven today conforms to Bergman’s rule, with larger individual coming from the north and smaller ones from the south [38]. This is not unequivocal as the population measured from Poland [38] does not follow the latitudinal trend as it has smaller individuals than expected. This implies that any conclusion along these lines should be formed with care. The Pin Hole ravens are all larger than the small Middle Pleistocene fossils from La Fage named *Corvus antecorax* (now *C*. *corax antecorax*) [51,71].

Amongst these raven elements are many parts of the skeleton of adult and immature individuals. The existence of immature bones and the relatively even distribution of all skeletal elements (albeit in a small sample), may suggest that the species bred on the rock outcrops forming Creswell Crags. Even distributions of elements in birds, such as those found in ravens at the Middle Pleistocene cave site of La Fage in France, probably suggest that a species is living and dying at the site as it indicates a relative lack of destructive depositional (taphonomic) processes [72]. Ravens today live in a variety of habitats and are known to regularly, although not solely, breed on rock faces [73]. They are omnivorous and their diet consists of everything from carrion (their most important food) through to small mammals, eggs and nestlings, littoral invertebrates and grassland insects, as well as seeds, fruit and other vegetable matter [73]. They are generally sedentary.

There are also two remains from smaller corvids which are more difficult to identify as there are a number of European candidates which differ in average measurements but overlap in the ranges of measurements. The latter signifies that identification may involve a range of possibilities. Therefore, the immature right femur (PH(F) 8069) (Fig 5l) falls in the size range of the jay *Garrulus glandarius*, magpie *Pica pica*, nutcracker *Nucifraga caryocatactes*, Alpine chough *Pyrrhocorax graculus* and jackdaw *Corvus monedula* according to the published measurements [42]. The left coracoid (PH(F) 9326) meanwhile is immature to the extent that the sternal articulation is not completely formed and the measurements are likely to be unreliable. Morphological characters described by Tomek and Bochenski [42] may also be problematic due to the lack of maturity of both the coracoid and the femur. The femur is, however, more like that of a jackdaw than any other smaller corvid [42]. If the smaller corvid remains belong to those of a jackdaw this would be in keeping with the frequent use of rock outcrops for nesting and roosting by that species. The immaturity of the bones is consistent with that scenario.

In addition to those described above, a number of specimens cannot be securely assigned to species or even family within the Passeriformes (see S1 File). Passeriformes are notoriously difficult to identify and little interpretation of such undetermined material can be made as there are many species in the group occurring in a great variety of habitats including deciduous and coniferous woodland as well as open habitats such as marshland, tundra, grasslands, moorland etc. There is also material from the Middle Palaeolithic level at Pin Hole which has been identified as merely that attributed to birds (see S1 File) and so cannot be interpreted at all.

## Discussion

### Changes in Ecological Community and Geographical Range

The bird remains give indications regarding the local and regional environment surrounding Pin Hole during early MIS 3, as well as forming part of the environment themselves. The dates, based on radiocarbon and ESR, from the Middle Palaeolithic levels at Pin Hole indicate that the bird remains are likely to date from the earlier parts of MIS 3, 40 ka ^14^C (ca. 44 ka cal BP) up to about 50 ka ^14^C years BP [18,24]. This time episode ends with Greenland stadials (GS) 12 and encompasses further such stadials as well as warm interstadials. Also included during this time is Heinrich (H) event 5, whose temperatures were apparently colder than the glacial maximum of the preceding colder MIS 4, and possibly the subsequent H4 [74,75].

The environmental inferences that can be taken from the birds present in the early MIS 3 deposits of Pin Hole are outlined in Table 2. Included amongst these are mountain, moorland and tundra taxa such as the two *Lagopus* species (*L*. *lagopus* and *L*. *muta* (Fig 6B)). There are also taxa that today breed in tundra and disperse to maritime or southern regions outside the breeding season such as the skua (*Stercorarius* cf. *parasiticus* / *longicaudatus*), the likely dotterel (cf. *Charadrius morinella*), the brent goose (*Branta bernicla*) (Fig 6A), the scoter (*Melanita* sp.) and the diver (*Gavia* cf. *stellata* / *arctica*). Most of these taxa are only found at a considerable distance to the north of Pin Hole today or in the case of the migrants (scoter, brent goose and divers) are only generally found in coastal areas in the UK today. Next there is a category of taxa that today have distribution that are significantly further south and east, living in open steppe to semi-desert environments like the demoiselle crane (cf. *Anthropoides virgo*) (Fig 6C) and the long-legged buzzard (*Buteo* cf. *rufinus*). To this category one can add the Alpine swift (*Tachymarptis melba)* which is similar although with a more Mediterranean focussed distribution in Europe (Fig 6D). There are taxa with very widespread distributions such as the raven (*Corvus corax*) (Fig 6F), the possible jackdaw (*Corvus* cf. *monedula*), the possible kestrel (*Falco* cf. *tinnunculus*) as well as the undetermined small and large falcons which tell us very little as they are mostly tied to rocky outcrops or trees for nesting and live in many different habitat terrains and are widespread in the Palaearctic today [49]. There are also taxa that are associated with various kinds of open habitats including grasslands such as the short-eared owl (*Asio* cf. *flammeus*), larks (Alaudidae) and starlings (*Sturnus* sp.) as well as taxa that usually nest in trees such as woodpigeon (*Columba* cf. *palumbus*) (Fig 6E), herons (*Ardea* cf. *cinerea*) and storks (*Ciconia* cf. *ciconia* / *nigra*). The undetermined thrush remains (*Turdus* sp.) require habitats with trees or at least shrubs to nest in. The eagle owl / snowy owl *Bubo* cf. *bubo* / *scandiaca* is difficult to interpret because the two species that could represent the Pin Hole material have different habitat requirements (Table 2). If the remains represent the snowy owl they would belong to the tundra habitat birds while if they are eagle owl then they can represent a much broader range of vegetational habitats including steppe, deciduous or boreal woodlands. Finally, there are the large number of birds which imply a local presence of water such as the many undetermined Anseriformes remains, the probable common scoter and Eurasian teal, the goldeneye or long-tailed duck, the brent goose, as well as the diver, the probable grey heron and possibly also some of the waders.

**Table 2:** Ecological affinities of the Middle Palaeolithic birds from Pin Hole. Data from [45, 49,66,144–146].

Taxon	Modern habitat	Breeding Distribution today	Sedentary / Migratory	Nesting sites	Diet
*Gavia* cf. *stellata* / *arctica*	Tundra into taiga (mostly *G*. *arctica*) close to water in breeding season. Large lakes and near-shore marine in winter.	Sub-arctic through boreal taiga to upper temperate zone.	Migrates between freshwater inland to inshore marine when water freezes.	On shallow fresh water or no further than 1.2m from water.	Fish
*Ardea* cf. *cinerea*	Usually associated with trees and bushes near water mainly in lowlands. Shallow freshwater preferred, flowing or still. Also on grassland.	Temperate zone.	Migratory, partial migrant or dispersive.	Usually tall trees but will nest on cliff ledges, in reed beds or on the ground.	Fish, amphibians, small mammals, insects, reptiles.
*Ciconia* cf. *ciconia* / *nigra*	*C*. *ciconia*: Open marshland. *C*. *nigra*: grassland or dense forest feeding in freshwater margins and open marshy areas. More open on migration.	Mid latitude continental Europe and Mediterranean.	Migratory. Europe to sub-Saharan Africa.	Mostly tall trees but sometimes cliff ledges.	Animals exclusively: amphibians especially frogs, insects, fish and small mammals.
*Branta* cf. *bernicla*	Low tundra with pools usually near the sea during breeding season. Shallow sea coasts and estuaries in winter with mudflats.	High Arctic Greenland and Spitzbergen.	Migratory. High Arctic (Summer) to coastal western Europe (Winter).	Dry hummock above flood level, often on small islands in rivers or offshore.	Vegetation by grazing and in shallow water.
*Anas* cf. *crecca*	Coastal tundra through steppe to desert fringes in breeding season. Winters mostly in temperate zone in Western Europe	Mid-latitudes up to Arctic fringes.	Mostly migratory. Small numbers sedentary in mid latitudes.	On ground in thick sheltered vegetation.	Omnivorous. Seeds and invertebrates.
*Melanita* sp.	Tundra or dwarf heath in breeding season. Marine waters in flocks in winter. *M*. *fusca* more associated with trees than *M*. *nigra*.	Low Arctic to boreal. *M*. *fusca* more boreal than *M*. *nigra*.	Migratory. Inland to sea.	On ground in concealed vegetation near water.	Mainly molluscs.
*Buteo* cf. *rufinus*	Plains, semi-desert, mountains and other treeless habitats.	Low to low-middle latitudes.	Migratory, partially migratory or resident.	On crags and low rocks. Often shaded. Occasionally on flatter ground or rarely trees.	Small mammals, reptiles and large insects.
*Falco* cf. *tinnunculus*	Diverse habitats except tundra, forest tundra and secluded taiga. Frequent in moorlands, heathland, frassland, forest fringes but avoids open wetlands, large forests and high mountains.	Widespread from southern almost to most northern latitudes of Palaearctic.	Migratory in north and east but partially migratory to dispersive.	Trees or rock ledges.	Small vertebrates. Chiefly small mammals with birds usually secondary.
*Falco* sp. (Small)	Diverse habitats depending on taxon.	Widespread depending on taxon.	Migratory to partially migratory and dispersive depending on taxon.	Trees or rock ledges.	Small vertebrates and insects.
*Falco* sp. (Large)	Diverse habitats depending on taxon.	Widespread depending on taxon.	Migratory to partially migratory and dispersive depending on taxon.	Trees or rock ledges.	Small vertebrates. Predominantly birds or small mammals depending on taxon.
*Lagopus lagopus*	Treeless moorland and tundra.	Arctic, subarctic, boreal zones and into northern part of temperate zone	Resident.	On the ground among thick vegetation.	Plants including heather, willow and birch, *Vaccinium*, *Empetrum* and sedges.
*Lagopus muta*	Treeless moorland and tundra on higher ground than *L*. *lagopus*.	Arctic, subarctic to boreal. Also on Arctic Alpine in middle latitudes.	Resident.	On the ground in the open partly sheltered.	Plants including heather, willow and birch, *Vaccinium*, *Empetrum* and sedges.
cf. *Anthropoides virgo*	Breeds in steppe regions in plains and high valleys with coarse grasslands and *Artemisia* brush with areas of bare salt-flats. In winter—sandbanks and marshes.	Middle latitudes between boreal and arid zones.	Migratory. Middle latitudes of Eurasia to Sub-Saharan Africa and India.	Shallow scrape on the ground.	Largely plant material with some invertebrates.
cf. *Charadrius morinella*	Mountain tops above the treeline and below the snowline. In tundra in breeding season and stony steppe, semi-desert in winter.	Arctic tundra and Arctic-Alpine.	Migratory.	On the ground in short vegetation or on bare gravel or soil.	Mostly insects and spiders. Some other invertebrates and plant material.
*Stercorarius* cf. *parasiticus* / *longicaudatus*	Coastal and inland barren moorlands and tundra in breeding season. *S*. *parasiticus* more coastal than S. *longicaudatus*. Along coasts on passage and pelagic in winter.	High Arctic to low Arctic boreal and cool temperate zones.	Migratory. Movement between sea and land.	On the ground. In open areas.	Feed on rodents, birds and insects if breeding inland, fish during winter and if breeding at coast.
*Columba* cf. *palumbus*	All forms of woodland. Avoids treeless areas, dense wetlands and rocky mountains.	Upper and lower middle latitudes. Continental and oceanic. Marginally in steppe and Mediterranean where wintering occurs.	Mainly migratory in north and east. Partial migrant in rest of distribution.	In trees and less often in thick vegetation on the ground.	Plants mainly: Seeds, nuts and fruit, green leaves etc.
cf. *Asio flammeus*	In Arctic tundra, boreal, temperate, steppe and Mediterranean zones. Open plains, moors, downs, rough hillsides, heaths, marshes and dunes.	In Western Palaearctic from high to middle latitudes.	Migratory to partial migrant	On ground, in thick cover or occasionally in open.	Small mammals: Mostly rodents and particularly voles.
*Bubo* cf. *bubo* / *scandiaca*	*B*. *bubo*: Forest, open woodland and open areas such as floodlands, heaths, grasslands. *B*. *scandiaca*: Beyond treeline on open tundra, sea-level to uplands.	*B*. *bubo*: boreal, temperate and steppe zones. *B*. *scandiaca*: High Arctic.	*B*. *bubo*: Resident in Europe, dispersive in Russia. *B*. *scandiaca*: Partial migrant and nomadic.	*B*. *bubo*: On rock ledge, on ground or in hole in tree. *B*. *scandiaca*: On ground, elevated on rock outcrop or hummock.	*B*. *bubo*: Mammals from water vole to hare, birds from jays to mallard. *B*. *scandiaca*: On tundra voles and lemmings, elsewhere rabbits and medium-sized birds
*Surnia ulula*	Open boreal coniferous forest with clearings and moors in lowlands or mountains.	Boreal zone in Eurasia and North America.	Resident but invades the south during some Atumns.	Nests in cavities on top of broken trunks, natural tree hollows, abandoned holes of large woodpeckers.	Takes mainly small mammals as prey, mostly lemmings and voles. Will also take birds, frogs and occasionally fish.
*Tachymarptis melba*	Over most habitats.	In lower middle latitudes, from Mediterranean, and steppe to temperate zones.	Migratory.	Ledge or hole in rock faces.	Arial plankton—insects, spiders etc.
Alaudidae	Open country if skylark.	Tundra (shore lark) to desert to semi-desert (long-billed and small-billed desert larks) through temperate grasslands (crested larks), temperate steppe (stout-billed larks).	Partial migrant.	Ground	Insects and seeds
*Turdus* sp.	Broad leaf and conifer woodland and scrub (If redwing—birch forest).	Arctic/Alpine, boreal to warm temperate zones.	Partial migrant to migrant.	Tree or bush	Earthworms, snails and other invertebrates and berries
*Sturnus* sp.	*S*. *vulgaris* / *unicolor*: Open woodland and grasslands. Avoids dense forests and arid areas. Often associated with cattle. *P*. *roseus*: semi-desert and steppe.	*S*. *vulgaris* / *unicolor*: Upper to lower middle latitudes. *P*. *roseus*: Lower to lower middle latitudes of Palaearctic.	*S*. *vulgaris* / *unicolor*: Migratory in north and east, more sedentary south and west. *P*. *roseus*: Migratory Down to India and Sri Lanka.	*S*. *vulgaris* / *unicolor*: Holes in trees or rocks. *P*. *roseus*: Hole among stones in scree.	*S*. *vulgaris* / *unicolor*: Animals and plants. Insects from turf and fruit. *P*. *roseus*: In breeding season mostly insects (grasshoppers and locusts) and otherwise much plant matter also.
*Corvus* cf. *monedula*	Boreal, steppe, temperate and Mediterranean lowlands.	Across middle and upper middle latitudes.	Resident to migratory	Rock crevices to hollow or shady tree	Invertebrates, fruit, seeds, carrion and occasional small vertebrates.
*Corvus corax*	Ubiquitous.	Arctic to Tropics.	Sedentary and dispersive in the north.	Trees and rock ledges, inland or at coast.	Carrion, small vertebrates and invertebrates and plant foods

Amongst these birds from the Pin Hole Middle Palaeolithic levels are included a number of predators and scavengers which may have relied on some of the vertebrate species found in the MIS 3 levels of Pin Hole (Text B in S1 File). These are the owls, long-legged buzzard and falcons as well as the skua. The skua and snowy owl today rely on good lemming and vole numbers to breed [76] and these rodents are well known from MIS 3 Europe.

It would appear that the mixture of bird taxa in the early MIS 3 assemblage at Pin Hole form a non-analogue community similar to those that have been identified during this time episode among other organisms including mammals and plants [65]. Such communities have been noted amongst birds on a number of sites [77]. It has been noted that European MIS 3 mammalian faunas often consist of non-analogue associations with species whose geographical ranges do not overlap today and including northern tundra and taiga, eastern steppic and more southern temperate species [78–84]. These faunas are also known from the mid to northern latitudes of Asia and North America (including Beringia) [85–88]. Accompanying the non-analogue mammals were non-analogue plant communities [89,90,91]. The latter had been suggested earlier from a study of plant macroscopic remains [92] although it was later explained as being due instead to a mixing of assemblages from discrete climatic episodes by Coope [93] who studied beetles from the same deposits.

So the first question about the Pin Hole bird assemblage from early MIS 3 is whether they form a contemporary community. Clearly the dates indicate that at least 15 thousand radiocarbon years are covered by the Middle Palaeolithic deposits in Pin Hole which include a great deal of climatic variation. The question, therefore, arises as to whether this climatic variation explains the assemblage of apparently contradictory bird species and that they represent a palimpsest, as suggested by Coope [93] for the mixed beetle assemblages. AMS carbon dates would be needed on individual skeletal elements of the contradictory bird taxa to resolve this as was achieved for small mammals of the Late Glacial of North America and Russia [94]. The problem with doing so on the birds from Middle Palaeolithic level (early MIS 3) of Pin Hole is that they are of a general age where the standard errors on the dates are an order of magnitude longer than for Late Glacial dates in the mammalian study [94]. This signifies that correlation between the dates and the millennial scale climatic events of MIS 3 are more difficult to achieve. Therefore, we have to take a broader brush approach to this issue and consider if the assemblage as a whole, or its constituent parts, are consistent with what we believe we know about MIS 3. For example, the mixture of northern tundra and eastern steppe taxa are to be expected within the so-called steppe-tundra or mammoth-steppe that has been evoked by mammalian palaeontologists for Europe during MIS 3 (and the Late Pleistocene in general) [94,95].

The non-analogue assemblage of birds from the MIS 3 has implications to how geographical range change takes place in birds. The dominant theory to explain these communities is that species respond to changes in climate and environment (the Gleasonian response) rather than ecological communities as a collective (the Clementsian response) [87–90,65]. Species are therefore said to be individualistic in their response. This signifies that species will vary in their speed of expansion and contraction of geographical range in relation to climate change. These differences will inevitably lead to ecological communities being transient, particularly given climatic regimes that are without equivalent today [96]. The greatest climatic anomaly, from a modern perspective, between faunal elements is between the northern Arctic and the continental steppic taxa and not between the Arctic and temperate taxa [82,83].

The individualistic nature of responses to climate change accounts for the fact that some species are in refugium during glacials while others are in their expansive phases. It also accounts for the fact that species have different refugial locations, sizes and ecologies [97]. The Arctic, boreal and steppe taxa would appear to be in their expansive ranges while the more temperate taxa should be in refugium. This is why the more temperate, and particularly the woodland, taxa in the MIS 3 levels at Pin Hole are more challenging to some views of the ecology of this time period [98]. An explanation could lie in the Cryptic Northern Refugium Hypothesis [99], an additional perspective to the southern peninsular refugium view of Hewitt [100], which is gaining some support [101,102] albeit not without controversy [103]. Evidence from palynology for a temperate arboreal component of the vegetation in Northern England during MIS 3 was recently published [104] and controversial aDNA evidence for boreal trees in Scandinavia during the LGM ([105] but see [106]). The pollen from speleothems in northern England dated to MIS 3 contained considerable amounts of oak pollen although the implications of this to the locations of refugia in Britain at that time were not explored [104]. Other support for the Cryptic Northern Refugium Hypothesis, indeed most of it, comes not from Quaternary palaeoecology but from phylogeography, the biogeography of distinct genetic populations [107], and aDNA studies but remains controversial.

The Quaternary fossil record is influential in the proposal of hypotheses for phylogeography [108]. An example being the idea that cryptic northern refugia had existed in the Late Pleistocene [99]. The importance of the fossil record in mediating any interpretations of phylogeographic studies was illustrated by the debate over the rock sandpiper in Beringia [109–112]. The debate centred on when it is that cold adapted species are in refugium. Pruett and Winker [109] believe that species like the rock sandpiper were in refugium during the last glacial while Stewart and Dalén [110] suggested that instead the species was in its expansive phase at that time. The latter is because cold adapted species are in their most restricted range today, during the Holocene interglacial rather than during the last glacial, as illustrated by the bird identifications herein.

It is therefore worth considering how the bird species described here affect the interpretations of corresponding published phylogeographies. The number of phylogeographies of birds in general is relatively low compared with mammals although some species identified from MIS 3 levels at Pin Hole have received attention. These include the raven [113–115], the common starling [116], the woodpigeon [117], the rock ptarmigan [118,119], the willow ptarmigan [120,121] and the snowy owl [122]. Of these there are widespread Holarctic species like the raven whose phylogeographic pattern today is not very differentiated with New World and Old World subclades within a mixed Holarctic clade and a haplotype shared between the Old and New Worlds [114]. The fossil ravens from MIS 3 Pin Hole merely provide further evidence of the species’ adaptable, and hence widespread, nature. Indeed for species with Holarctic distributions that also have a great latitudinal range it is less clear when they are in refugium as they may always have had a relatively large and continuous range that included the mid-laltitudes of the Holarctic.

The snowy owl is more problematic as the fossils are not conclusively identified as such. However, the snowy owl, if it is represented at Pin Hole, has no phylogeographic structure today [122] and the fossils confirm that this Holarctic species had a greater spread south in the Late Pleistocene than at present. It is wide ranging, although it is clearly in refugium during interglacials such as that today. The Pin Hole owl fossils may provide confirmation of the species broader range in the past, similar to the rock sandpiper, which in this case is not reflected in the phylogeography.

The rock ptarmigan meanwhile has only been studied in Beringia and North America, although the pattern observed is that there exists today a great deal of genetic differentiation among populations that is suggested to represent distinct refugia during the Late Pleistocene [118]. It is further proposed that these populations recolonised the North after the Pleistocene ice retreated. This paper makes the assumptions made by Pruett and Winker [109], that the species was in refugium during the last glacial. The MIS 3 Pin Hole fossils, together with a great many fossils from Europe [62], confirm that this is not the case and that the species was in its expansive phase rather than its refugial phase in the last glacial. Clearly the rock ptarmigan is in its interglacial refugium in the north and in Alpine areas today [110]. This suggests that the population differentiation probably dates from either before the last glacial, perhaps during the last interglacial (the Eemian / MIS 5e) because this was likely the last time the populations would have been cut off from each other or that it occurred since the last glacial. The latter would perhaps best explain the patterns seen in the Aleutian Islands where different genetic populations live on different islands [119]. These differentiations are less likely to be formed during the glacials when contact was more likely due to low sea levels and larger populations than during interglacials when sea-levels are high and populations smaller and more allopatric.

The willow ptarmigan, however has received a broader sampling programme in the study by Högland et al. [120]. Two patterns were visible in the Western Palaearctic at different phylogenetic hierarchies. First an East-West division among the samples, with Russian samples clustering with North American samples while all Western samples clustered together. This split did not respect the currently accepted subspecies, as *L*. *l*. *lagopus* was either side of the East-West divide and *L*. *l*. *lagopus* and *L*. *l*. *scoticus* were in the Western clade. The fossils of *Lagopus lagopus* at Pin Hole are evidence of the species’ former wider distribution which, when isolated into populations in the British Isles and Scandinavia, likely caused the differentiation of the island population into the red grouse *L*. *l*. *scoticus*. Whether this differentiation took place since the Pleistocene or during an earlier interglacial is not yet known [121].

The starling is similar to the snowy owl as its fossils are difficult to confirm as representing common starling *S*. *vulgaris*, although if we assume that they come from the *S*. *vulgaris* / *S*. *unicolor* clade they can inform interpretations of their phylogeography. They are divided into distinct species *S*. *vulgaris* and *S*. *unicolor* and subspecies with a biogeographic structure. This pattern is not however reflected in the genetics with common haplotypes shared between Spain, England, Scotland, Norway and even the Faeroes, where a distinct subspecies is located (*S*. *v*. *faroensis*) [70]. In addition there is more variation within *S*. *vulgaris* than between *S*. *vulgaris* and *S*. *unicolour*. This pattern seems at odds with the possible mechanism outlined for the evolution of the biogeographic pattern in *Sturnus* in Europe [61]. The mechanism being that the sedentary populations of the *S*. *vulgaris* / *S*. *unicolor* clade evolved from migratory populations and that becoming sedentary leads to allopatry [61]. *S*. *unicolor*, although only represented by 2 individuals in Neves et al [116], does form a separate clade within *S*. *vulgaris* but would seem to have diverged more recently than the age of the ancestor of all *S*. *vulgaris*. This means that the relative lack of migratory behaviour in *S*. *unicolor* may well sustain its distinction from *S*. *vulgaris*. In fact *S*. *unicolor* is paraphyletic with respect to *S*. *vulgaris* which is consistent with the idea that relatively low migratory behaviour causes a type of allopatry [61]. The Pin Hole specimens, assuming they represent *Sturnus* rather than *Pastor*, demonstrate that these starlings were widely distributed in MIS 3 in a similar way to the raven and were probably not differentiated genetically. Their possible migratory behaviour, indicated by their relative small sizes (see below), may contribute to removing any phylogeographic structure due to greater amounts of population mixing.

The wood pigeon meanwhile has a phylogeographic structure today with some distinct haplotypes restricted to the north, in the eastern Baltic region, in addition to haplotypes found both in the north and south [117]. This pattern is certainly one that could be explained by the existence of northern populations during the last glacial, in eventual cryptic northern refugia (during the LGM), although the phylogeographic study [117] did not include a thorough survey of Europe. Phylogeographic patterns today are, however, not wholly adequate to confirm the existence of northern refugia and the MIS 3 record of woodpigeon may add support to this possibility, albeit not a dated LGM record that would be the ultimate confirmation for a cryptic northern refugia.

### Size and shape differences in Late Pleistocene bird species

There are a number of bird remains from the MIS 3 of Pin Hole which appear to be anomalous in measurements with respect to modern populations of the species that they are thought to be attributable to. This assumes they represent extant species, which is not easy to demonstrate although it is more certain for some taxa than for it is for others. The two *Lagopus* species are perhaps the best example of taxa whose specific taxonomic identity is not in any serious doubt and show consistent differences in the past from the present. The latter has been noted by a number of authors [63,51,57,38]. The early MIS 3 Pin Hole material includes tarsometatarsi with more robust shaft dimensions as well as humeri with larger proximal breadths in both *Lagopus lagopus* and *L*. *muta* than in all modern populations examined [57,38] (Fig 7). Such differences seem to be caused by greater body mass which in turn may reflect the high carrying capacity of the MIS 3 environment [57,38]. However, due to the allometric nature of the larger size of these birds it may alternatively be due to an adaptive character such as greater development of flight musculature reflected in the greater breadth of the proximal humerus [57]. The latter may in turn reflect greater mobility and the additional weight caused by the greater muscle bulk is reflected in the tarsometatarsus [38]. This suggestion is contradicted by studies of the ecomorphology of migratory birds where mobility is generally correlated with lower bird weights [123]. Others have attributed the greater body mass of these birds to Begmann’s rule [63], the thermoregulatory rule that predicts that warm blooded vertebrate populations living in colder climates have larger body masses. A Europe wide metrical survey of Late Pleistocene *Lagopus* suggested that this greater size was not due to Bergmann’s rule as no latitude size cline was noted and all populations were consistently larger [38].

The wood pigeon carpometacarpus is also reasonably confidently identified although again it differs metrically from the corresponding bones of the modern species. In this case it exceeds in greatest length (GL), shaft breadth (KB) and distal metacarpal symphysis breadth (BS) a sample of 24 specimens of wood pigeon measured by Fick [40]. Fick’s sample was from Germany and Austria and so it is possible that in other parts of the woodpigeon’s range today they have similarly longer carpometacarpi. For example, it would be interesting to compare the Pin Hole specimen to a sample of woodpigeon carpometacarpi from a more migratory population because the species is more migratory to the north and east [45,49]; although it should be noted that the species is regarded as generally lacking in size or wing measurement variation across Europe today [143]. Migratory populations of bird species often have longer carpometacarpi relative to their humeri in line with the ecomorphological traits of migratory birds [123]. This phenomenon may be discerned in the fossil record given the availability of suitable data [61,38].

Other bird remains, from the MIS 3 deposits of Pin Hole, with measurements that fall outside the modern ranges for taxa are the brent goose humerus, the Alpine swift ulna, the demoiselle crane premaxilla and the Arctic or long-tailed skua distal humerus. The brent goose humerus had a shaft breadth dimension that exceeded a large range of the species today (Table C in S1 File), the demoiselle crane premaxilla was broad for its length (Fig F in S1 File) and the skua humerus fell between the Arctic and long-tailed skua dimensions (Fig D in S1 File). The Alpine swift meanwhile had a distal ulna depth that exceeded a sample of modern and Late Pleistocene Iberian individuals (Fig E in S1 File). All these taxa are interesting occurrences at Pin Hole as they exceed the geographic range for these species today (Fig 6A, 6C and 6D), certainly if they come from breeding individuals whose geographic range had expanded.

The robusticity of the brent goose humerus is difficult to interpret but would seem to imply that the birds had greater weights which have many biological correlates in birds [38]. One interesting correlate of larger size is that it can be indicative of a greater degree of sedentary, as opposed to migratory, behaviour although this cannot be confirmed and this would be unexpected for a migratory species such as this.

The anomalously sized and proportioned birds, from a modern point of view, mirror the patterns seen in mammals in the Late Quaternary [124–127]. Quaternary fossils of extant vertebrates, and particularly mammals and birds, with morphologies not seen today have been known for some time although they were often interpreted as distinct and hence extinct species and subspecies [126,51,61]. The Alpine swift is an interesting example here as the ulna seen in Fig E in S1 File demonstrates it is large and falls outside the breadth range of the two modern Alpine swifts available as well as the fossil remains attributed to the species from Devil’s Tower in Gibraltar. It is interesting that Jánossy [60] named a large swift *Tachymarptis* (formerly *Apus*) *submelba* from the Middle Pleistocene of Europe. Clearly the Pin Hole material could belong to that species although it is equally possible that *T*. *submelba* should be synonymised with *T*. *melba* (see [61]). An alternative view on these distinct individuals with different morphologies (sizes and shapes) is that they represent differently adapted populations of extant taxa [63,51,124–127].

The most often-quoted hypothesis to account for change in vertebrate body size during the Quaternary is Bergmann's Rule (see above). Bergmann’s rule has a long history of study, particularly using observations of patterns of size across geography today [128]. Debate has existed over both the degree to which species conform to Bergmann’s Rule [129,130] and the cause of these spatial patterns in body size [128]. In their survey of birds and mammals worldwide [130] it was found that 72% of birds and 65% of mammals conformed to the rule. They also found that sedentary birds were more likely to follow the rule than migratory birds. Explanations of the patterns observed vary from the original heat conservation hypothesis, through phylogenetic hypotheses to resource availability and starvation resistance hypotheses [128]. The original explanation was thermoregulation, namely that a larger body mass had a correspondingly lower surface area from which to loose heat than a smaller body mass which was therefore an advantage in colder climates [128]. Current views suggest that starvation resistance hypotheses or the availability of food are the most likely explanations for the size clines observed today [124,131,132]. It is interesting to note however that the proximal mechanism causing the size change has been recently suggested to be phenotypic plasticity rather than a genetic microevolutionary response [133].

Many Pleistocene mammals and birds from the last glacial were larger than today [124], and some authors have suggested that thermoregulation is the causal mechanism due to the generally colder conditions at the time [63,125]. There are of course many other ecological correlates of size variation which may be causally linked to it [38], and some palaeontologists and biologists have argued that Bergmann’s Rule has been applied where it may not be appropriate and that the subject is a much more complex one [134]. A proposed counterargument [135,95,136] is that it is not the climate that directly affects an animal's size but the consequences of the length and quality of the plant growing season, which in turn are affected by climate. This view is more in line with current explanations for Bergmann’s rule (e.g. [132]). The vegetational environment of MIS 3, called steppe-tundra or mammoth-steppe, has been described as very productive on the basis of the large herbivores it supported [95]. The vegetation was a mosaic of high diversity, although dominated by grassland. It should be noted, however, that some palynologists have disagreed with the concept of the mammoth-steppe. They believed that the vegetation was poor, a polar desert, based on the apparently low pollen influx at the time. The idea that the vegetational environment was a rich steppe-tundra was expanded by Lister and Sher [137], who have suggested that the steppe-tundra vegetation relied on a climatic regime that has vanished. They pointed out that detailed climatic records, such as studies of the Greenland ice cores, have shown that the Holocene is distinct from the Late Pleistocene in having unusually stable conditions. Pleistocene climatic instability may have allowed the mosaic vegetation of the steppe-tundra to persist. Once this climatic regime ceased to exist, the megafauna, which relied so heavily on the vegetation type the climate supported, changed along with it. Some animals became globally extinct, like the giant deer *Megaloceros giganteus* (Blumenbach) and the woolly rhinoceros *Coelodonta antiquitatis* (Blumenbach), or locally extinct, like the lion *Panthera leo* Linnaeus [138]. Others underwent a reduction in body size, such as the horse [127]. It is, therefore, an attractive hypothesis that certain birds, such as the two species of *Lagopus*, which abounded in the steppe-tundra environment, also underwent changes upon its demise, such as reduced geographic ranges and body size. Interestingly, the largest subspecies of the genus, *L*. *I*. *major*, lives today on the steppes of Kazakhstan which may be an analogous situation although the limited specimens available of *L*. *l*. *major* are isometrically large unlike the Pleistocene members of *Lagopus* (Fig 7) [38].

The metrical shape differences can be difficult to interpret as some represent size differences that are conferred to the skeletal elements allometrically. The *Lagopus* species differences, probably those of the brent goose and possibly those of the Alpine swift seem to represent size related differences. The skua bone may well be a large individual long-tailed skua but because it is not completely clear which skua species is represented it could be a small Arctic skua (Fig D in S1 File). The premaxilla of the demoiselle crane, assuming it belongs to that species, would meanwhile seem to be anomalous in shape rather than size. This may suggest that the species was somehow differently adapted at that time and/or that the population of the species in Britain (and North Western Europe) during the Late Pleistocene may have been genetically distinct from ones further south and east. It is not possible to explain the difference seen in the specimen from Pin Hole. It is notable, however, that the measurements taken on the modern demoiselle cranes (Fig K in S1 File) show a great deal of variation in bill-tip breadth relative to bill-tip length as shown by the poor statistical correlation between the two (Fig F in S1 File).

Subsequently the anomalous mammals and birds had been interpreted as being populations that were adapted to conditions in the past that no longer exist [139,38]. These differently adapted populations were probably assumed to have evolved into modern populations in the same areas today and were therefore phylogenetic ancestors to the modern populations [38]. Generally, the extinct form (species or subspecies) that had been described were thought to have been on questionable grounds [61]. While the nature of metrical characters is still considered questionable by one of us (JS) for the naming of extinct taxa, evidence is emerging to suggest that some of these anomalous fossils may represent extinct populations after all. This is because the significance of the distinct mammals is undergoing a reinterpretation due to the availability of data informing our understanding of their genetics. Ancient DNA studies of European Late Pleistocene lion *Panthera leo*, Arctic fox *Alopex lagopus* and Beringian wolf *Canis lupus* have shown that the past populations were both distinct genetically as well as ecomophologically [140,64,141]. For example, the Arctic fox of the Late Pleistocene in Belgium and Germany was found to represent extinct genetic population [64]. These extinct southern Arctic foxes were distinct in having smaller feet than modern northern populations [142,64]. This suggests an alternative interpretation of the distinct Late Pleistocene bird remains particularly when they can be shown to be consistently different across time and space from modern populations, as has been shown for the two *Lagopus* species in Europe. It may be that these Late Pleistocene populations became extinct as their geographical ranges contracted at the end of the Pleistocene as has been shown for the Arctic fox. Another example is among Late Pleistocene wolves where individuals appear adapted to hunting or scavenging megafauna [141]. These extinct populations were adapted to an environment no longer available and died out rather than evolved into modern forms. This may explain their anomalous morphologies, from a modern point of view. It may be, therefore, that these extinct populations should be considered to be related to the species level extinctions taking place at the end of the Pleistocene [83]. It is unclear, however, whether the distinct bird populations generally represent different (extinct) populations like the Arctic fox or are cases of phenotypic plasticity. It will be interesting to see whether the anomalous morphologies of the birds described above represent distinct genetic populations.

## Conclusion

The bird remains from the early MIS 3 deposits of Pin Hole represent over 20 different taxa with wetland birds dominating the assemblage species diversity. In absolute numbers, it is the Galliformes of the genus *Lagopus* that dominate the assemblage. In addition, there are taxa that lived in the cave or on rock outcrops, birds of open habitats and ones that require woodland to breed. The taxa identified here have added some important new taxa not on the original list of birds from the Middle Palaeolithic of Pin Hole such as the new records of long-legged buzzard *Buteo rufinus* and the skua *Stercorarius parasiticus* or *S*. *longicaudatus*. There have been a few that are not confirmed including the common gull now identified as the skua. Other taxa not confirmed are ones whose identities are difficult to identify due to the conservative skeletal anatomy of the Order including Anseriformes such as barnacle goose, white-fronted goose, ruddy shelduck, tufted duck, goosander, Passeriformes like skylark, woodlark, waxwing, redwing, hawfinch and Charadriiformes such as snipe and knot. The other taxa listed here are all confirmations of Bramwell [17] identifications and so, due to the inclusion of woodland species in MIS 3 levels, confirm his interpretations that these birds were in Britain before the Holocene, albeit in MIS 3 rather than in the Late Glacial.

The fact that they were here before the LGM signifies that these populations may be unlikely to have survived to contribute to the eventual Holocene/modern populations of the species seen today. Unfortunately the mixed nature of the upper fauna signifies that Late Glacial records of such taxa recorded by Bramwell [17] cannot be confirmed without an expensive and destructive radiocarbon dating program, some of which would likely entirely destroy the representative specimens. The climatic requirements of the birds in the MIS 3 levels at Pin Hole are varied and represent perhaps the most interesting result of the study as northern Arctic/Alpine tundra, steppe to Mediterranean as well as temperate taxa are included. This mirrors the environments indicated by the mammals in the MIS 3 levels of Pin Hole [18] as well as North West Europe more widely [78,82,84]. The latter is in line with the other indications for a non-analogue environment at this time. The mixture of steppe and tundra species signify that the birds from Pin Hole MIS 3 deposits represent the avian equivalent of the well documented steppe-tundra mammals of the Late Pleistocene of Europe and equivalent latitudes across the Palaearctic and into North America.

The occurrence of the birds from the North of England during MIS 3 also has an important contribution to make to the understanding of the historical biogeography of the species and particularly those with modern phylogeographic studies. In the case of the wood pigeon they may add support to an interpretation that northern refugial populations persisted through the last cold stage, while other taxa give a better context to understanding their broader climate and environmental adaptations or tolerance.

Accompanying the distinctive ecological associations of the birds from MIS 3 Pin Hole are some distinct morphologies. These morphological differences mostly appear to represent size differences in the MIS 3 Pin Hole populations which may either be of evolutionary significance or representative of phenotypic plasticity. The birds that manifest different sizes are the two *Lagopus* species, the brent goose, the Alpine swift and the wood pigeon which are all larger than their conspecifics in Europe today. The skua may also be larger although because it plots between the two smallest skuas today it is not clear whether it is a large long-tailed skua or a small Arctic skua. Either way, the explanations for these size differences (larger birds) are likely to driven by differences in carrying capacity in the past rather than the direct effects of thermoregulatory rules like the traditional explanation for Bergmann’s Rule. Finally, there is an anomalous demoiselle crane premaxilla which is more difficult to explain. It maybe that MIS 3 North-West European demoiselle cranes differed in diet from their conspecifics living to the South and East of Europe today.

The analysis of the birds from Pin Hole therefore demonstrate that the kinds of responses that birds undergo in response to long term climate change are very similar to that of mammals with both geographical displacement, resulting in non-analogue communities, as well as morphological change visible. At this stage it is, however, not known if the morphological changes detected are due to phenotypic plasticity or have a genetic mechanism and may hence involve evolutionary process. It should also be mentioned that some of the identifications here described, based on the morphology of the fossils may prove to be imprecise or even wrong once they are successfully analysed using aDNA methods [143].

## Supporting Information

S1 File Supporting texts, tables and figures. Text A in S1 File. Pin Hole—Site Description and Excavation History. Text B in S1 File. Taphonomy of the Pin Hole Bird Remains. Text C in S1 File. List of Pin Hole Middle Palaeolithic Bird Remains with Provenance. Text D in S1 File. Measurements of #MR40 (female mallard *Anas platyrhynchos*) in author (JS) collection. Table A in S1 File. Measurements of *Ardea* sp. humerus shaft probably from Pin Hole MIS 3 level PH(F) 5895 A in relation to those of those of modern Ardeid specimens [47]. Table B in S1 File. Measurements of Pin Hole MIS 3 level *Ciconia* sp. coracoid PH(F) 5896 in relation to those of those of modern European specimens [48]. Table C in S1 File. Measurements of large Anseriformes humeri and coracoidea from MIS 3 of Pin Hole together with comparative measurements [49,50]. Table D in S1 File. Measurements of *Melanita* spp. humeri and tarsometatarsi from the Middle Palaeolithic of Pin Hole together with comparative measurements [49,50]. Table E in S1 File. Measurements of Pin Hole MIS 3 small *Falco* remains and modern comparative material. Included are the present author’s measurements, taken on specimens from the Natural History Museum’s Collections at Tring, together with those in brackets from Solti [51]. Table F in S1 File. Measurements of Pin Hole MIS 3 carpometacarpus PH(F) 2155 in relation to those of modern specimens of Columbidae [52] and Pteroclidae (authors measurements). Table G in S1 File. Measurements of owl tarsometatarsi from the Pin Hole MIS 3 level in relation to those of those of modern specimens [53]. Table H in S1 File. Measurements of the carpometacarpi of Turdidae showing the Pin Hole MIS 3 specimen in relation to extant *Turdus* species [36]. Table I in S1 File. Skeletal element representation of Galliformes and Anatinae at Pin Hole compared to West Runton, Boxgrove and the La Fage ravens (*Corvus antecorax*) [37,38,39, 54]. Figure A in S1 File. Percentage ratio diagram of the Pin Hole MIS 3 anatine bone measurements. Ratios are expressed as percentage of a female mallard. Also shown are the modern range of the mallard *Anas platyrhynchos* for each measurement (the grey area in the middle of the diagram) as well as that for the teal *A*. *crecca* (the smallest European duck, the grey area to the left of the diagram) both taken from [50]. All the numbers in the diagram are Pin Hole specimen numbers from the Armstrong excavation and would normally have the prefix PH(F). Figure B in S1 File. Scattergram with the tibiotarsus distal breadth measurement versus distal depth of the long-legged buzzard *Buteo* cf. *rufinus* from the Middle Palaeolithic of Pin Hole together with those of modern comparative specimens of European buzzards and hawks. Figure C in S1 File. Scattergram with carpometacarpus greatest length measurement versus proximal breadth of *Falco* cf. *tinnunculus* from Middle Palaeolithic of Pin Hole together with those of modern comparative specimens of smaller falcon. Figure D in S1 File. Scattergram with humerus distal breadth measurement versus measurement X (see Figure K in S1 File) of skua from Middle Palaeolithic of Pin Hole together with those of modern comparative specimens of *Stecorarius*. Figure E in S1 File. Scattergram with ulna greatest length measurement versus distal depth of *Tachymarptis melba* from Middle Palaeolithic of Pin Hole together with those of modern comparative and Pleistocene specimens of swifts Apodiformes. Figure F in S1 File. Scattergram with measurements (Mandible length Ml and Mandible breadth Mb, see Figure K) taken on the premaxilla of *Anthopoides virgo* from Middle Palaeolithic of Pin Hole together with those of modern comparative specimens of the species. Figure G in S1 File. Scattergram with humerus greatest length measurement versus shaft breadth of Alaudidae from Middle Palaeolithic of Pin Hole together with those of modern comparative specimens of European larks. Figure H in S1 File. Scattergram with humerus proximal breadth measurement versus shaft breadth of starlings from Middle Palaeolithic of Pin Hole together with those of modern comparative specimens of European starlings. Figure I in S1 File. Scattergram with carpometacarpus greatest length measurement versus proximal breadth of raven *Corvus corax* from Middle Palaeolithic of Pin Hole together with those of modern comparative specimens of European ravens and the Middle Pleistocene raven *C*. *corax antecorax* from France. Figure J in S1 File. Scattergram with tarsometatarsus distal breadth measurement versus shaft breadth of raven *Corvus corax* from Middle Palaeolithic of Pin Hole together with those of modern comparative specimens of European ravens and the Middle Pleistocene raven *C*. *corax antecorax* from France. Figure K in S1 File. Measurements taken on *Anthropoides virgo* premaxilla and Stercorariidae distal humeri. *Anthropoides virgo* premaxilla: Mb—premaxilla length from distal ends of the concavitas palati to distal tip, Ml—premaxilla breadth at distal ends of the concavitas palati; Stercorariidae distal humerus: Bd—breadth of distal humerus as described in figure; X—diagonal measurement from processus supracondylaris dorsalis to epicondylus ventralis; SD—depth of humerus shaft at distal end at the proximal margin of the fossa musculi brachialis.
(DOC)
