# Correction: The Long Term Response of Birds to Climate Change: New Results from a Cold Stage Avifauna in Northern England

**DOI:** 10.1371/journal.pone.0133107

**Published:** 2015-07-14

**Authors:** The PLOS ONE Staff

## Notice of Republication

This article was republished on June 26, 2015, to correct Fig 4, which was darker than intended. The publisher apologizes for the error. In addition, the family names "Scolopacidae" and "Charadriidae" have been un-italicized in Table 1, and tracked changes have been removed from S1 File. Please download this article again to view the corrected version.
