# Supplementary material for: The Long Term Response of Birds to Climate Change: New Results from a Cold Stage Avifauna in Northern England
Source: PLoS One. 2015 May 20;10(5):e0122617. doi: 10.1371/journal.pone.0122617 (PMC4439125; doi:10.1371/journal.pone.0122617)
Supplement: S1 File — Text A in S1 File. Pin Hole—Site Description and Excavation History. Text B in S1 File. Taphonomy of the Pin Hole Bird Remains. Text C in S1 File. List of Pin Hole Middle Palaeolithic Bird Remains with Provenance. Text D in S1 File. Measurements of #MR40 (female mallard Anas platyrhynchos) in author (JS) collection. Table A in S1 File. Measurements of Ardea sp. humerus shaft probably from Pin Hole MIS 3 level PH(F) 5895 A in relation to those of those of modern Ardeid specimens [47]. Table B in S1 File. Measurements of Pin Hole MIS 3 level Ciconia sp. coracoid PH(F) 5896 in relation to those of those of modern European specimens [48]. Table C in S1 File. Measurements of large Anseriformes humeri and coracoidea from MIS 3 of Pin Hole together with comparative measurements [49,50]. Table D in S1 File. Measurements of Melanita spp. humeri and tarsometatarsi from the Middle Palaeolithic of Pin Hole together with comparative measurements [49,50]. Table E in S1 File. Measurements of Pin Hole MIS 3 small Falco remains and modern comparative material. Included are the present author’s measurements, taken on specimens from the Natural History Museum’s Collections at Tring, together with those in brackets from Solti [51]. Table F in S1 File. Measurements of Pin Hole MIS 3 carpometacarpus PH(F) 2155 in relation to those of modern specimens of Columbidae [52] and Pteroclidae (authors measurements). Table G in S1 File. Measurements of owl tarsometatarsi from the Pin Hole MIS 3 level in relation to those of those of modern specimens [53]. Table H in S1 File. Measurements of the carpometacarpi of Turdidae showing the Pin Hole MIS 3 specimen in relation to extant Turdus species [36]. Table I in S1 File. Skeletal element representation of Galliformes and Anatinae at Pin Hole compared to West Runton, Boxgrove and the La Fage ravens (Corvus antecorax) [37,38,39, 54]. Figure A in S1 File. Percentage ratio diagram of the Pin Hole MIS 3 anatine bone measurements. Ratios are expressed as [file pone.0122617.s001.doc]

S1 Supporting Information

Text A Pin Hole - Site Description and Excavation History

The Middle Palaeolithic (Marine Oxygen Isotope Stage 3) deposits of Pin Hole, Creswell Crags, Derbyshire in England have yielded the bird remains that form the topic of this paper. The material was collected by A.L. Armstrong during the 1920’s and 1930’s, between 1924-1936 [1-5]. Pin Hole is one of four caves at Creswell Crags to have yielded both Pleistocene fauna and Palaeolithic artefacts. Creswell Crags is a shallow gorge incised in the Lower Permian Magnesian Limestone on the borders of the counties of Derbyshire and Nottinghamshire in the English East Midlands. Pin Hole is on the Derbyshire (northern) side of the gorge and at its western end (Figure 1). The cave is a narrow, solutionally enlarged linear fissure some 46 metres (m) in length. This linear fissure is the “main passage” and it widens locally to create the “outer” and “inner chambers”. A short passage leads off eastwards from the inner chamber (Figure 2).

There have been several excavations inside Pin Hole, in addition to those of Armstrong. Mello and Heath dug at its entrance in 1875 [6,7] and smaller scale excavations have taken place after Armstrong’s work, by Collcutt in 1974 [8] and most recently by Jenkinson from 1984-1989. This latest excavation is as yet unpublished.

In the main passage Armstrong explored a distance of 80´ (24.4 m) inwards of a datum point at the cave entrance. This included a completion of the initial 22´ (7.0 m) which had been partially excavated by Mello and Heath. He also discovered and excavated the “eastern passage” and the “trefoil-shaped chamber” at its inner end.

Armstrong recognized two principal sediment bodies - an “upper (red) cave-earth” and a “lower (yellow) cave-earth”. At the beginning of his excavation (from November, 1924 – July, 1925) Armstrong kept a *Register of Finds* which lists all the artefacts discovered and supposed humanly modified fauna. From this it is apparent that the small number of artefacts from the lower cave-earth are all likely to be Late Middle Palaeolithic (Mousterian), while those from the upper cave-earth are of very mixed ages ranging probably from the Late Middle Palaeolithic to the Neolithic and with the largest number of Upper Palaeolithic date.

Each of the finds listed in the *Register* was recorded in terms of its distance into the cave, as measured from an entrance datum, and depth, as measured directly downwards from a thin flowstone at the surface of the deposits. After the abandonment of the *Register*, Armstrong continued to record distance into the cave and depth below the flowstone for most of the artefacts excavated from the main passage and for many of the bones and teeth, particularly those which seemed capable of identification or were in any way unusual. This information was marked in pencil on the specimens at the time of excavation and these were often subsequently re-marked in ink. However, Armstrong no longer systematically recorded from which of the cave-earths individual items came.

The flowstone can be traced along the walls of the main passage and can be seen to rise irregularly from near the front towards the back of the cave. Its geometry has been recorded by Jenkinson [9]. Using the contours of this flowstone as it extends back into the cave and the information recorded by Armstrong on many of the finds it is possible to re-plot these onto a longitudinal profile of the main passage [10,11]. When this is done, the archaeological material can be seen to divide stratigraphically. In the lower part of the plot all the artefacts appear to be Late Middle Palaeolithic (Mousterian), but in the upper part the pattern discerned from the entries in the *Register* is confirmed and there is clear mixing of artefacts of several different ages.

There is a similar division within the large-mammal fauna. Its lower part documents a long period of faunal accumulation by spotted hyaenas (*Crocuta crocuta*). This is the fauna which has been used to define the Pin Hole mammal assemblage-zone of the Middle Devensian [12]. Principal prey species were wild horse (*Equus ferus*) and woolly rhinoceros (*Coelodonta antiquitatis*) and there are important groups of fossils from lion (*Panthera leo*), woolly mammoth (*Mammuthus primigenius*), giant deer (*Megaloceros giganteus*) and reindeer (*Rangifer tarandus*).

There are now numerous radiocarbon determinations for this lower fauna and these complement electron spin resonance (ESR) and Uranium-series dates in suggesting an age for it of between 55-40 kyr BP [10,11]. Some stratigraphic inversions amongst the radiocarbon determinations indicate that individual older bones have been moved so that they now seemingly overlie younger bones. It is likely that these have been eroded from older sediments at the upslope (inner) end of the cave-fill and re-deposited above younger sediments downslope. Burrowing by hyaenas may have been another cause of the translocation of bones. The birds whose identifications we report here were found with this lower fauna.

The upper parts of the cave sediments, as could have been predicted from their archaeological content, have yielded a fauna whose components are of varying ages. Radiocarbon determinations for single bones and teeth indicate a presence of Middle Devensian fauna and it is assumed that these have been derived from the older part of the cave-fill, perhaps, in part, due to the activities of badgers (*Meles meles*). There are in addition bones, for example of Arctic hare (*Lepus timidus*), reindeer and wild cattle (*Bos primigenius*), which have been shown by radiocarbon dating to be of Lateglacial age and cut-marks indicate that some of the hare bones owe their presence in the cave to Later Upper Palaeolithic hunters. There are also bones of domestic animals, such as sheep (*Ovis aries*) and pig (*Sus scrofa*). We have not re-examined the birds from these upper sediments although they were considered important by Bramwell and led him to suggest that woodland birds had arrived in Britain before the Holocene, during the Late glacial. A small number of remains are mentioned because they represent important and unusual records of bird taxa in Britain (Category D in the list of material below).

The sample which we have included in this study consists of all the bird bones from the main passage whose co-ordinates indicate as being parts of the deeper Middle Devensian fauna. Armstrong also divided the length of the main passage, between where Mello and Heath finished their excavation at 22´ (7.0 m) from the entrance and his termination at 80´ (24.4 m), into seven sections termed A-G from front to back. Smaller bones were often attributed to just a section rather than a measured distance from the entrance datum. Bird bones with only section and depth marked on them have been included in this study when the depth measurement indicates that they could only have been part of the Middle Devensian fauna.

The very smallest bones, probably those recovered by sieving with a ¼ inch mesh, were stored by Armstrong in carefully labeled tins and boxes. Most of these have been re-packed in recent times. Since we are uncertain how accurately the original information has been transcribed and the original associations preserved, we have not included into our study bones for which there are only recent labels or labels in an unfamiliar handwriting i.e. script not recognized as belonging to Armstrong. Inevitably, this means that the bones of the smaller birds are under-represented in our sample. On the other hand, hopefully, we have guarded against the inclusion into the Middle Devensian fauna of spurious records by the use of strict criteria for acceptance of bird remains as belonging to the Middle Palaeolithic level (see below).

The eastern passage leading to the trefoil-shaped chamber (Figure 2) were recorded by Armstrong in a slightly different way. Near its entrance bones and artefacts were still documented in terms of the main passage with distance into the cave and depth below the flowstone recorded on them. In addition to these measurements, Armstrong either recorded an off-set measurement of distance into the eastern passage or simply added the suffix ‘P’ to the first two measurements to indicate an origin for the specimen in the entrance to the eastern passage. Further in, where this became impractical, he cleared its contents in three (assumed) horizontal spits recorded as 10´P, 11´P and 12´P, with depth measured from a datum at the entrance to the eastern passage. Conjoins between flints and teeth whose parts were found in the main passage and in the eastern passage suggest that the sediments sloped downwards into the eastern passage. The fauna from its filling is Middle Devensian and it is apparent that this is the part of the cave where hyaena denning had been most intense.

However, we have listed the birds from the eastern passage separately as there are records from here of yellow-necked mouse (*Apodemus flavicollis*). The yellow-necked mouse are considered to be Postglacial immigrant into the British Isles [13] and its presence in the eastern passage may be evidence of disturbance, most probably by burrowing. A possibility that some of the bird bones from this area may also be intrusive has to be considered and, for this reason, the avifauna from the eastern passage is listed as separate categories (EP1 and EP2) in the list of Pin Hole bird remains (see below). Alternatively, it is possible that the species *A. flavicollis* was genuinely living at the latitude of Pin Hole during MIS 3. While there are no records of *A. flavicollis* at this latitude in Europe at this time there are records of *A. sylvaticus*, a closely related congener with a similar geographic range today, in northern Europe during MIS 3 [14,15].

Text B Taphonomy of the Pin Hole Bird Remains

Bird remains in cave deposits, like those of mammals, often have multiple accumulation modes. The taxa identified from the Middle Palaeolithic levels of Pin Hole include birds that are likely to have lived in and around the cave as well as ones that today have no association with rock faces and/or caves. Among the cave and cliff dwelling birds are possibly the large owl, the Alpine swift, the raven, the possible jackdaw, the starlings, the long-legged buzzard and the large and small falcons, including the kestrel. Eagle owls nest on rock faces and in old nests of birds in trees while snowy owls usually nest on the ground in tundra. Alpine swifts today nest in caves and on cliffs [16] (Table 2). Many of the small and large falcon species nest today on rock ledges as well as in trees [17] (Table 2). Present day ravens and jackdaws are often found on cliffs and quarry exposures and use rock ledges for nesting [18] (Table 2). Finally, starlings will use holes in trees or rock faces to nest [19]. Therefore the remains of these birds may be explained as deaths at their roosting or nesting sites. The immaturity of the possible jackdaw remains as well as some of the raven remains appears to confirm this interpretation.

Many of the other birds found in the early MIS 3 level of Pin Hole, including the many waterfowl and the gallinaceous birds, would not generally be found living in or around caves and rock faces. There is, however, a caveat to this for the waterfowl and that is that the cave may have been at some close proximity to an open water body in the vicinity of Creswell Crags during the Late Pleistocene [20]. This may explain the large number and variety of wetland birds present in Pin Hole. The local presence of a water body may be the source of the water birds but it does not explain how these non-rock-face or non-cave-dwelling birds were deposited in the cave. There is no indication that the sediments in the cave possessed any characteristics of a water-lain deposit in fact they have been interpreted as being formed “by the accumulation of autochthonous sediments, derived from weathering of the cave walls” [21]. The river Poulter in the gorge is likely to have been present during MIS 3 which may help explain matters although it may be that standing water was also present nearby. The remains of pike *Esox lucius* in the Middle Palaeolithic levels of Pin Hole [20] would be consistent with the local presence of a water body in the form of either a slow flowing river or a lake.

The most likely mechanism is that a predator was responsible for the non-cave and non-rock face dwelling bird remains from Pin Hole and although a single predator species is unlikely to have accumulated all the remains, the large size of some taxa, like the geese, crane and heron, restricts the likely possible candidates. Evidence that one or more predatory mammals were responsible for the bird assemblage exists in the relatively frequent occurrence of gnaw marks on the remains. These consist of small holes punctured into the articular ends of the bones as well as crenulated margins to bone breaks, particularly on shafts where articular ends are missing. These marks would seem to indicate that small carnivorous mammals had predated the remains bringing them into the cave. Of the predatory species found in the Middle Palaeolithic deposits at Pin Hole [10] the red fox *Vulpes vulpes* and the two polecats *Mustela putorius* and *Mustela putorius robusta* are the most likely candidates, and for the smaller birds the stoat *Mustela erminea* may have additionally contributed. The diet of all these mammals today includes some birds [22]. The possibility exists, however, that the marks were caused by the scavenging of remains already in the cave and so those four taxa may not have accumulated the remains in the first instance. Other mammalian predators present in the assemblage include the spotted hyaena *Crocuta crocuta* (probably responsible for the large mammal bones in the assemblage), lion *Panthera leo*, wolf *Canis lupus* and brown bear *Ursus arctos*, all of which could have conceivably contributed bird remains but would not have produced the small holes and are not thought to include birds as significant prey.

The final mammalian predator that potentially contributed bird remains to the Middle Palaeolithic assemblage at Pin Hole is the Neanderthal. Recent work on bird assemblages in Gibraltar and elsewhere has claimed that Neanderthals exploited birds and particularly raptors and corvids [23-32]. The evidence includes that where cut marks have been found on the long bone shafts of various skeletal elements that was used to suggest that Neanderthals were using flight feathers of these birds. This evidence is new and the assumption had been that the exploitation of birds by Neanderthals was at best rare. It would, however, be good to see an analysis of the cut-marks on bird bones done using the methods of Bello [33] which is the most objective method for validating cut-marks. No cut marks, or signs of charring, have been found on the birds remains from the Middle Palaeolithic levels of Pin Hole so there is no reason to suppose that Neanderthals were involved in their accumulation at this site.

It is likely that the bird remains from Pin Hole were at least partly accumulated by birds of prey. The avian predators and scavengers in the assemblage are the eagle owl / snowy owl, the possible short-eared owls, long-legged buzzard, small falcons (including the probable kestrel), the large falcon and the raven. The large owl *Bubo* cf. *bubo* / *scandiaca*, particularly if it represents an eagle owl, is likely to have contributed to the assemblage if, as has been suggested [34], eagle owls may have been the principal accumulators of bird remains in Pleistocene cave deposits in Poland. Of course the possible candidates for accumulation are not restricted to the taxa found as remains in the cave itself and many other birds could have contributed, particularly species that live in tundra, or temperate situations today. The best physical evidence for accumulation by an avian predator or scavenger is an undetermined passerine sternum (PH(F)446) with V-shaped notches along the keel, a classic result of avian predation, or perhaps scavenging [35,36].

Using the present day habits of the birds represented in the assemblage as well their potential predators to invoke the source of the bird remains can be augmented by an examination of the proportions of the different remains. For example, if remains are the result of deaths at nesting and roosting sites it is likely that a more equal representation of skeletal elements will exist, as shown by the relatively even representation of skeletal elements of ravens at La Fage in France [37,38] (Table I). The La Fage raven assemblage is biased towards a better representation of more robust elements but not as severely, as for example, the ducks from open-air sites such as Boxgrove and West Runton [39,38] (Table I). Biases in the representation of more robust elements (coracoids of Anseriformes and tarsometatarsi of Galliformes) are due to the greater likelihood that processes such as predators, weathering, erosion and compaction will negatively impact, in a mechanical way, on less robust elements. Therefore, it is interesting that the ravens from Pin Hole, although in small numbers, include a cranial element such as the os premaxillare (bill) and other normally under-represented elements such as the radius.

The percentage representation of skeletal elements was calculated for the two most common taxonomic categories of remains at Pin Hole, the Anatinae and the medium-sized Galliformes (Table I). The ducks demonstrate a departure from the results for all open-air sites studied with the best represented elements being the humeri and tarsometatarsi, while in the open-air sites it is the coracoid followed by the carpometacarpus or ulna [39,38]. If the open-air site pattern of skeletal elements represents a bias caused by relative mechanical strength [40,39] the Pin Hole pattern in Anatinae may be due to a mixture of mechanical strength (the humerus is relatively well represented in open-air sites as well as at Pin Hole) and bones that are from meat-poor regions of carcasses which makes them less likely to being completely destroyed during feeding. This would explain the large numbers of tarsometatarsi of Anatinae at Pin Hole. It is notable that the pattern of skeletal element representation is unlike that reported by Ericson [41] where anterior (wing) bones outnumbered posterior (leg) elements in ‘natural’ accumulation and the opposite for humanly produced assemblages.

The Galliformes have the same best represented elements as the Anatinae at Pin Hole, although the tarsometatarsi vastly outnumber all other elements (Table I). The skeletal element ratios of medium sized Galliformes have received significant attention [42,43]. Mourer-Chauviré found that distal elements (tarsometatarsi and carpometacarpi) were likely to be over-represented due to bird of prey (owl) predation while proximal limb bones (humeri and femura) would be due to human predation. In the Pin Hole assemblage the tarsometatarsi vastly outnumbered other elements with 58.5% of all elements, although they were followed by the humerus at 13.8%, while the carpometacarpus is only at 6.2% and the femur at 9.2%. This result is a mixture of distal and proximal limb bones scoring highly, The reason for this is unclear although a non-human accumulator is perhaps most likely without any evidence to show otherwise. This is consistent with the lack of cut marks or charring that would have suggested human predation. The humans responsible for the Middle Palaeolithic archaeology are Neanderthals and it has been suggested that there is very little, if any, convincing evidence for the exploitation of birds or smaller mammals by Neanderthals [44-46] although recently this position has changed [23-32].

Text C List of Pin Hole Middle Palaeolithic Bird Remains with Provenance

Key to provenance

The provenance categories relate to the recording methods of A.L. Armstrong and the way in which the data is recorded (see above). The specimen numbers are listed below and start with the prefix ‘PH(F)’.

MP (Very likely Middle Palaeolithic):

A1 – Main passage >7 Ft, Bone labelled, regardless of whether in ink or pencil.

A2 – Main passage >7 Ft, Bone not labelled.

PMP (Probably Middle Palaeolithic):

B1 – Main passage 6.5 – 7 Ft., Bone labelled, regardless of whether in ink or pencil.

B2 – Main passage 6.5 – 7 Ft., Bone not labelled.

EP1 – East Passage and trefoil chamber, Bone labelled, regardless of whether in ink or pencil.

EP2 – East Passage and trefoil chamber, Bone not labelled.

C1 – One set of dimensions (assumed to be depth), Bone labelled, regardless of whether in ink or pencil.

C2 – One set of dimensions (assumed to be depth), Bone not labelled.

Unprovenanced

No provenance recorded.

Not MP (Not Middle Palaeolithic):

D – Taxa of interest but not included in provenance categories A, B or C.

R – Specimen not included in any of the above provenance categories but which had been accepted by Jacobi.

Notes:

- Based on information in Jacobi *et al.* (1998) any number under 20 Ft. is assumed to be depth. This is thought to be reasonable because Armstrong (the original excavator of the material) collected from 20 Ft. onwards into the cave and so numbers lower than 20 should indicate depth and not distance into the cave.

Gaviformes

*Gavia arctica* / *stelata*

PMP Material:

Left ulna, Immature. (PH(F) 18602) (11’ – 12’/P). (prev. id. as *Gavia arctica* by D. Bramwell and *G. stellata* by C.J.O. Harrison). (Measurements: GL – 11.76, Did – 12.4). (EP1) (Photo).

Ciconiformes

*Ciconia* sp.

MP Material:

Right coracoid, Immature. (PH(F) 5896) (G/9’) (prev. id. as *C. ciconia*). (Measurements: Lm – 74.08 after Gruber 1990) (A1) (Photo).

*Ardea cinerea*

MP Material:

Right coracoid, gnawing damage. (No PH(F) No.). (E/7’). (not prev. identified). (No measurements). (A1) (Photo).

Left humerus, gnawing visible on proximal and distal the end (PH(F)18565) (11’ 73) (prev. id as *Ardea cinerea*). A1 [But not accepted by R.J. so excluded from this study]

Not MP:

Left Humerus shaft (PH(F) 5895 A) (G/2’0”) (prev. identified as *Anser anser* by D. Bramwell and *A. cinerea* by C.J.O. Harrison). (Measurements: SC – 10.28). (R).

Anseriformes

*Branta*  cf. *bernicla*

MP Material:

Left humerus (PH(F) 18543) (F/9’/64 (P), (prev. identified as *Branta bernicla* by D. Bramwell). (Measurements: GL –123.9, SC – 8.62). (A1) (Photo).

Large Anseriformes

MP Material:

Right coracoid (PH(F) 13083) (8’ 67) (prev. identified as *Anser brachyrhynchos* by D. Bramwell and *Branta leucopsis* by C.J.O. Harrison). (Measurements: GL – 62.06, LM – 54.90, BF – 24.70, SC – 7.4). (A1).

Left coracoid fragment (PH(F) 18542) (F/9’) (prev. identified as *Branta bernicla* by D. Bramwell and *Branta leucopsis* by C.J.O. Harrison). (Measurements: SC – 7.3). (A1).

Proximal right femur (PH(F) 7905) (E/10’) (prev. identified as *Anser brachyrhynchus* by D. Bramwell and *Anser albifrons*  by C.J.O. Harrison). (No measurements possible). (A1).

Furculum symphysis (PH(F) 2947) (D/6’-6) (prev. identified as *Branta leucopsis* ‘Barnacle size’ by D. Bramwell?). (No measurements possible). (A1).

Left scapula (PH(F) 18567) (8/62 - 70) (prev. identified as *Branta leucopsis?* by D. Bramwell). (No measurements possible). (A1).

Right coracoid (PH(F) 18540) (8/63’) (prev. identified as *Branta bernicla* by D. Bramwell). (Measurements: GL – 56.18, LM – 49.76, BF – 21.22, SC – 6.2). (A1).

Right scapula fragment (PH(F) 281) (A/7’) (prev. id. as undet. Aves sp.). A1 [Not on original Jacobi list]

PMP Material:

Left tarsometatarsus (PH(F) 13065) (8’ – 12’) (prev. identified as *?Tadorna ferruginea* by D. Bramwell and *T. tadorna* by C.J.O. Harrison). (Measurements: GL – 57.97, Bp – 12.57+, Bd – 13.05, SC – 5.10). (C1).

Right coracoid (PH(F) 5895 B) (12’/P although on bag and presumably incorrect 2’/3’ or O) (prev. identified as *Anser anser* by D. Bramwell and *A. fabalis* by C.J.O. Harrison). (Measurements: GL – 67.2, LM – 60.12, BF – 25.82, SC – 8.12). (EP1)

Distal left humerus (PH(F) 13082) (8 – 12’) (prev. identified as *Branta bernicla* by D. Bramwell). (Measurements: Bd – 16.18). (C1).

Anterior sternal articulation (PH(F) 13090 A) (8/12’) (prev. identified as *Anser brachyrhynchos* or *A. albifrons* by D. Bramwell ). (No measurements possible). (C1).

Anterior sternal articulation (PH(F) 29197) (11 – 12’/P) (prev. identified as *Branta bernicla* by D. Bramwell ). (No measurements possible). (EP1).

Distal right tibiotarsus (no articulation – damaged) (PH(F) 13090 B) (9’ – 10’) (prev. identified as *Anser anser* by D. Bramwell and *A. albifrons* by C.J.O. Harrison). (No measurements possible). (C1).

Left scapula (PH(F) 7902 and 7903) (12/P) (Previously id as *Anser brachyrhynchos*? By D. Bramwell 1970 and *A. albifrons* by C.J.O. Harrison). EP1 [Not on original Jacobi list]

*Anas* cf. *crecca*

PMP Material:

Right humerus (PH(F) 9074) (11/P) (prev. identified as *Anas crecca* by D. Bramwell). (Measurements: GL – 62.12, Bp – 13.5, Bd – 9.2, SC – 4.3). (EP1).

*Melanita* cf. *fusca*

PMP Material:

Left humerus (PH(F) 7805) (12/P) (prev. identified as *Melanitta nigra* by D. Bramwell). (Measurements: GL – 96.09, Bp – 21.08, Bd – 12.0, SC – 5.84). (EP1). (Photo).

*Melanita* sp.

PMP Material:

Left tarsometatarsus (PH(F) 13084) (9’ – 10’) (prev. identified as *Melanitta nigra* by D. Bramwell). (Measurements: GL – 43.33, Bp – 10.38, Bd – 10.28, SC – 4.21). (C1) (Photo).

cf. *Clangula hyamalis / Bucephala clangula*

PMP Material:

Left humerus (PH(F) 7844) (6/64 or F/6’/64) (prev. identified as *Aythya fuligula* by D. Bramwell and *Clangula hyamalis* by C.J.O. Harrison). (Measurements: GL – 69.46, Bp – 17.00, Bd – 10.26, Sc – 5.26). (B1).

Anatinae

MP Material:

Right tarsometatarsus (PH(F) 7591) (50’-55/7’) (prev. id. as *L. lagopus*). (Measurements: GL – 40.47, SC – 3.83). (A1).

Right ulna – adult (prev. id as *Corvus corone*). (PH(F) 759). (B/8’-6). (Measurements: GL – 73.94; Did – 10.05; SC – 4.98). (A1).

Distal right carpometacarpus (PH(F) 145) (F/9) (prev. id. as *Lagopus* sp.) (Measurements: Bd – 7.24). (A1).

Distal left tibiotarsus (PH(F) 18597) (15’/74’) (prev. identified as *Mergus merganser* by D. Bramwell). (Measurements: Bd – 10.28, Dd – 9.87+). (A1).

Proximal left tibiotarsus with shaft (PH(F) 7813) (C/39/9’ or C/33/9’) (prev. identified as *Melanita nigra* by D. Bramwell and *Tadorna tadorna* by C.J.O. Harrison). (Measurements: Dip – 15.68). (A1).

Distal left humerus with shaft (PH(F) 7806) (9’/65) (prev. identified as *Melanita nigra* by D. Bramwell). (Measurements: Bd – 12.9, SC – 6.02). (A1).

Left coracoid fragment (PH(F) 518, PH(F) 524, PH(F) 42 or PH(F) 234) (50 – 54/9’ – 10’) (prev. identified as *Anas* sp. by D. Bramwell and *Mergus serrator* by C.J.O. Harrison). (No measurement). (A1).

Distal right humerus (PH(F) 518, PH(F) 524, PH(F) 42 or PH(F) 234) (50 – 54/9’ – 10’) (prev. identified as *Anas* sp. by D. Bramwell and *Mergus serrator* by C.J.O. Harrison). (Measurements: Bd – 12.7). (A2).

Left scapula fragment (PH(F) 518, PH(F) 524, PH(F) 42 or PH(F) 234) (50 – 54/9’ – 10’) (prev. identified as *Anas* sp. by D. Bramwell and *Mergus merganser* by C.J.O. Harrison). Diseased shaft. (Measurements: Dic – 12.18). (A2).

Distal right tarsometatarsus (PH(F) 518, PH(F) 524, PH(F) 42 or PH(F) 234) (50 – 54/9’ – 10’) (prev. identified as *Anas* sp. by D. Bramwell and *Mergus merganser* by C.J.O. Harrison). (Measurements: Bd – 9.45, SC – 4.63). (A2).

Left coracoid (PH(F) 651, PH(F) 141) (6’ D/9’) (prev. identified as *Anas platyrhynchos* by D. Bramwell and C.J.O. Harrison). (Measurements: GL – 53.87, Lm – 48.87, BF – 20.18). (A1).

Mandibular articulation (PH(F) 2613) (E 13’ – 15’) (prev. identified as *Anas platyrhynchos*). (No measurements). (A2).

Distal left humerus (PH(F) 7807) (8’/62 - 70) (prev. identified as *Melanitta nigra* by D. Bramwell). (Measurements: Bd – 13.10, SC – 6.03). (A1).

Left coracoid (PH(F) 7812) (8’/62’ – 70’) (prev. identified as *Melanitta nigra* by D. Bramwell). (Measurements: GL – 49.2, Lm – 45.56, BF – 20.02+). (A1).

Proximal radius (PH(F) 7820) (8/62’) (prev. identified as Anatidae by D. Bramwell and *Melanita nigra* by C.J.O. Harrison). (No measurements). (A1).

Distal right carpometacarpus (No PH(F) no.) (9 – 65’) (prev. identified as *Anas platyrhynchos* by D. Bramwell). (Measurements: Bd – 7.68). (A1).

Furculum symphysis (PH(F) 7811) (8’/62 - 70) (prev. identified as *Melanitta nigra* by D. Bramwell). (No measurements). (A1).

PMP Material:

Distal right humerus (PH(F) 8074) (9 – 10’) (prev. identified as *Anser erythropus*? by D. Bramwell and as a male *Mergus mergus* by C.J.O. Harrison). (Measurements: Bd – 13.06). (C2).

Right humerus shaft (No PH(F) No.) (P/11) (prev. id. as *Buteo buteo*). (Measurements: SC – 5.45). (EP1).

Left tarsometatarsus shaft (PH(F) 7609 – 7611) (55’ – 9’ 6’ – 7’) (prev. id. as *L. scoticus* and *L. lagopus*). (Measurements: SC – 3.8). (B2).

Right carpometacarpus (No PH(F) No.) (75’) (prev. id. as ??). (Measurements: GL – 56.95, Bp – 12.87, Bd – 7.11). (C1).

Distal right humerus (PH(F) 651, PH(F) 141) (6’ D/9’) (prev. identified as *Anas platyrhynchos* by D. Bramwell and *Melanitta nigra* by C.J.O. Harrison). (Measurements: Bd – 13.18). (B1).

Furcular symphysis (PH(F) 7819) (11/P) (prev. identified as Anatidae D. Bramwell). (No measurements). (EP1).

Proximal right humerus (PH(F) 9075) (F/6’) (prev. identified as *Anas querquedula* by D. Bramwell). (Measurements: Bp – 13.5). (B1).

Distal left humerus (PH(F) 13064) (8’ – 12’) (prev. identified as *Tadorna ferruginea* by D. Bramwell). (Measurements: Bd – 14.3). (C1).

Left coracoid fragment (PH(F) 18598) (12/P) (prev. identified as *Mergus merganser* by D. Bramwell). (No meassurements). (EP1).

Right ulna (PH(F) 7810) (12’/P) (prev. identified as *Melanitta nigra* by D. Bramwell). (Measurements: GL – 87.96, Bp – 8.77, Dip – 10.98, Did – 9.71, SC – 4.73). (EP1).

Proximal right carpometacarpus (PH(F) 8075) (9’ – 10’) (prev. identified as *Anas* sp. Mallard or Pintail by D. Bramwell). (Measurements: Bp – 11.83). (C2).

Proximal right tibiotarsus (PH(F) 7814) (12’/P) (prev. identified as *Melanitta nigra* by D. Bramwell). (No measurements). (EP1).

Sternum fragment (PH(F) 29238) (7’-6A) (prev. id. as undet. Aves sp.). B1 [Not on original Jacobi list]

Accipitriformes

*Buteo rufinus*

PMP Material:

Distal left tibiotarsus, Immature (No PH(F) No.) (P/10’ – 12’). (Measurements: BD - 14.58; TD - 9.76). (EP1). (Photo).

Falconiformes

*Falco* cf*. tinnunculus*

MP Material:

Right carpometacarpus (PH(F) 2614) (50’ – 55’/7’) (prev. identified as *Falco tinnunculus* by D. Bramwell and *F. subbuteo* by C.J.O. Harrison). (Measurements: GL – 28.22, Bp – 9.59, Bd - 6.06). (A2). (Photo).

*Falco* sp. (Small falcon)

MP Material:

Left humerus (A. 7’-6”). (PH(F) 18548). (prev. id as *Falco tinnunculus* by D. Bramwell and *F. vespertinus* by C.J.O. Harrison). (Measurements: GL – 50.74, Bp – 10.66, Bd – 8.64, SC – 4.27). (A1).

Distal left tarsometatarsus – grainy so immature (10’-12’/70). (PH(F) 9307). (From 75 specimens). (prev. id as *Falco tinnunculus*). (Measurements: Bd – 6.57, SC – 3.23). (A2). (Photo).

PMP Material:

Distal right tibiotarsus (11/P). PH(F) 18545). (On bag: “from Top 12”). (prev. id as *Falco tinnunculus*). (Measurements: Bd – 7.19, Dd – 5.21). (EP1).

Left tibiotarsus – immature as grainey (11’ – 12’/P). (PH(F) 8269). (On bag: “from (a) package 6 specimens”). (prev. id as *Falco tinnunculus*). (Measurements: GL – 61.73, Bd – 6.89, Dd – 5.49, SC – 3.08). (EP1).

*Falco* sp. *(*Large falcon)

PMP Material:

Proximal left tibiotarsus (PH(F) 8149) (12/P) (prev id as *Buteo lagopus* and *Falco peregrinus*). (EP1). (Photo).

Charadriiformes

Not MP:

*Alca torda* or *Fratercula arctica*

Right coracoid (PH(F) 8146) (Top of 0 – 1’0’’) (prev. id. as ?*Cepphus grille* by D. Bramwell and *Fratercula arctica* by C.J.O. Harrison). (D).

*Stercorarius parasiticus* or *S. longicaudatus*

MP Material:

Distal left humerus (PH(F) 7629) (D/6’) 362 (prev. id. as *Larus canus* by D. Bramwell and C.J.O. Harrison). (Measurements: BD – 11.32; SD – 4.56; X – 4.65). (D). (Photo).

*Charadrius morinella*.

MP Material:

Left humerus (PH(F) 7625) (D/9’) 311 (prev. id. as *Gallinago gallinago* and *Eudromias morinellus* by C.J.O.Harrison) (Measurements: GL – 40.32, SC – 2.88). (A1). (Photo).

Left humerus (PH(F) 12575) (F/7) (prev. id. as Charadriidae small by D. Bramwell and *Eudromias morinellus* by C.J.O.Harrison) (Measurements: GL – 43.2, SC – 2.95). (A1).

Undet Scolopacidae

MP Material:

Distal right tibiotarsus (PH(F) 8082) (9’ – 10’). (prev. id. as *Arenaria interpres* by C.J.O.Harrison). (Measurements: Bd – 4.38, Dd – 3.82). (A2).

PMP Material:

Proximal left femur (PH(F) 9322) (11’/P and 12’/P) (from 12 specimens). (prev. id as *Calidris canutus)*. (Measurements: Bp - 5.88). (EP2).

Undet Charadriiformes

MP Material:

Right carpometacarpus (PH(F) 18547) (8’/62-70) (prev. id as *Falco tinnunculus* and *Vanellus vanellus* by C.J.O.Harrison). (Measurements: GL – 38.93 - appears worm). (A1).

Columbiformes

*Columba palumbus*

MP Material:

Right carpometacarpus (D. 9’) (PH(F) 2155) (prev. id. as *Columba palumbus*) (Measurements: GL – 40.34, Bp – 11.68, Dd – 6.68, BS – 8.94, KB – 3.4). (A1). (Photo).

Strifgiformes

*Bubo* cf*. bubo* / *scandiaca*

PMP Material:

Distal left radius (PH(F) 7801 or 7800. 11/P). (prev, id. as *A. cinerea* by D. Bramwell). (Measurements: Bd – 12.36). (EP1). (Photo).

Post-MP/Unstratified Material:

Distal right carpometacarpus (PH(F) 8200). (Unprovenanced) (prev. id. as Nyctea sp.). (D).

*Asio cf. flammeus*.

PMP Material:

Left tarsometatarsus (prev. id as *Asio flammeus* by D. Bramwell and *Strix aluco*). (11’/12’/P). (PH(F) 7837 and 7838). (Measurements: GL - 45.37, Bp – 9.67, KC – 4.85, TP – 8.58, Td – 8.81, Bd – 10.58). (EP1). (Photo).

Right tarsometatarsus (prev. id as *Asio flammeus* by D. Bramwell and *Strix aluco*). (F/5’). (PH(F) 7837 and 7838). (Measurements: GL – 44.50, Bp – 9.15, KC – 4.74, TP – 8.42, Td – 8.22, Bd – Damaged). (D).

Undetermined Strigiformes (*Asio* / *Strix*)

MP Material:

Left coracoid fragment (PH(F) 1341). (D/9’) (prev. id as *C. corone* by D. Bramwell?). (No measurements). (A1).

*Surnia ulula*

Post-MP:

Right tarsometatarsus (PH(F) 8476) (3’ – 6’/50’ – 60’) (Measurements: GL – 24.72, Bp – 8.62, Bd – 9.82, SC – 5.88) (prev. id. as *Surnia ulula*). (D). (Photo).

Apodiformes

*Tachymarptis melba*

MP Material:

Right carpometacarpus (PH(F) 8147) (7’ / 51 – 54) (prev. id as *A. melba* by D. Bramwell, confirmed by C.J.O. Harrison). (Measurements: GL – 26.59, Bp – 8.2). (A2).

Distal left femur (PH(F) 8147) (7’ / 51 – 54) (prev. id as *A. melba* by D. Bramwell, confirmed by C.J.O. Harrison). (Measurements: Bd – 4.25). (A2).

PMP Material:

Right tarsometatarsus (PH(F) 8145) (11’ – 12’/P) (prev. id as *A. melba* by D. Bramwell, confirmed by C.J.O. Harrison). (Measurements: GL – 14.64, SC – 1.92). (EP2).

Unprovenanced Material:

Right ulna (prev. id as *A. melba* by D. Bramwell, confirmed by C.J.O. Harrison). (Unprovenanced). (PH(F) 18600). (Measurements: GL – 25.62, SC – 3.1, Did – 5.4). (D). (Photo).

Galliformes

*Lagopus lagopus*

MP Material:

Right tarsometatarsus (PH(F) 266). (F/7). (prev. id as *Lagopus* sp. and *Lagopus lagopus*) (Measurements: GL – 39.42, BP – 7.20, KB – 3.62, BD – 7.84, DD – 8.58). (Photo).

Left tarsometatarsus PH(F) 3). (12/P). (prev. id as *Lagopus* sp. and *Lagopus lagopus*) ( (Measurements: GL – 39.62, BP – 8.64, KB – 3.30, BD – 7.44, DD – 8.44).

Right tarsometatarsus (PH(F) 2934 to 2941). (3’ – 6’/47 - 50). (prev. id as *Lagopus lagopus*) (Measurements: GL – 37.82, BP – 8.68, KB – 3.3, BD – 7.96, DD – 8.94).

Left tarsometatarsus (PH(F) 2934 to 2941). (D/7 – 5’/37-40). (prev. id as *Lagopus lagopus*) (Measurements: GL – 38.58, BP – 8.18, KB – 3.34, BD – 7.76, DD – 8.9).

Right tarsometatarsus (PH(F) 7616 to 7617). (F.10’). (prev. id as *Lagopus lagopus*) (Measurements: GL – 39.00, KB – 3.92)

Left tarsometatarsus (PH(F) 7568 - 7573). (50 – 54’, 9’ – 10’). (prev. id as *Lagopus mutus*) (Measurements: GL – 37.56, KB – 3.32).

Right tarsometatarsus (PH(F) 7568 - 7573). (50 – 54’, 9’ – 10’). (prev. id as *Lagopus mutus*) (Measurements: GL – 36.64, KB – 3.14).

Left tarsometatarsus (PH(F) 9308). (10’ - 12’/70). (On bag: from 75 specimens). (prev. id as *Lagopus mutus* by D. Bramwell). (Measurements: GL – 38.34, KB – 3.38). (A2).

Right tarsometatarsus (PH(F) 965). (8/63’) (prev. id as *Lagopus* sp. and *L. lagopus*). (Measurements: GL – 39.2, BP – 8.9, KB – 3.47, BD – 7.84). (A1).

Right tarsometatarsus (PH(F) 294). (10/60) (prev. id as *Lagopus* sp. and *L. lagopus*). (Measurements: GL – 40.24, BP – 8.5, KB – 3.52). (A1).

PMP Material:

Right tarsometatarsus (PH(F) 980). (8/CHB) (prev. id as *Lagopus* sp. and *L. lagopus*). (Measurements: GL – 40.24, BP – 8.56, KB – 3.6, BD – 8.2). (C1).

Right tarsometatarsus, damaged (PH(F) 8076 - 8082). (9’ – 10’). (prev. id as *L. mutus* by Colin Harrison). (Measurements: GL – 36.57, BP – 7.92, KB – 3.47). (On bag: from 12 specimens). (C2).

Right tarsometatarsus (PH(F) 7609 - 7611). (55 – 59’, 6’ – 7’). (prev. id as *Lagopus lagopus*) (Measurements: GL – 39.22, KB – 3.66). (B2).

*Lagopus muta*

MP Material:

Right tarsometatarsus (PH(F) 9). (F/7). (prev. id as *Lagopus mutus*) (Measurements: GL – 32.34, BP – 7.34, KB – 3.2, BD – 7.38, DD – 8.04). (Photo).

Right tarsometatarsus (PH(F) 529). (F/9). (prev. id as *Lagopus mutus*) (Measurements: GL – 33.54, BP – 7.36, KB – 3.08, BD – 6.76, DD – 7.9).

Left tarsometatarsus (PH(F) 1137). (F/7). (prev. id as *Lagopus mutus*) (Measurements: GL – 31.44, BP – 7.12, KB – 2.94, BD – 6.46, DD – 7.24).

Right tarsometatarsus (PH(F) 533). (F/7). (prev. id as *Lagopus mutus*) (Measurements: GL – 30.60, KB – 3.06, BD – 7.18, DD – 8.44).

Left tarsometatarsus (PH(F) 7565 to 7567). (50’ – 55’, 7’). (prev. id as *Lagopus mutus*) (Measurements: GL – 32.46, KB – 3.1).

Right tarsometatarsus (PH(F) 8071 - 8072). (9’ – 10’). (prev. id as *L. mutus* by D. Bramwell). (Measurements: GL – 31.32, BP – 6.84, KB – 2.84, BD – 7.30, DD – 7.13). (On bag: from 12 specimens).

Right tarsometatarsus (PH(F) 8059 - 8061). (7’ – 151 - 54). (prev. id as *L. mutus* by D. Bramwell). (Measurements: GL – 33.09, BP – 7.48, KB – 2.95, BD – 7.09, DD – 7.45). (On bag: from 19 specimens). (A2).

Left tarsometatarsus (PH(F) 1064). (10/64). (prev. id as *Lagopus mutus* by D. Bramwell). (Measurements: GL – 31.24, KB – 3.00). (A1).

Left tarsometatarsus (PH(F) 542). (8/62’ – 70’). (prev. id as *Lagopus mutus*). (Measurements: GL – 32.8, KB – 2.96). (A1).

PMP Material:

Right tarsometatarsus (PH(F) 190). (8’CHB, right depth but where in cave? Could be Trefoil Chamber?). (prev. id as *Lagopus mutus*). (Measurements: GL – 32.1, KB – 2.92). (C1).

*Lagopus* sp.

MP Material:

Distal left tarsometatarsus (PH(F) 7565) (50’ – 55’ / 7’) (prev. id. as *L. mutus*) (Measurements: BD – 7.7, DD – 8.6).

Proximal left tarsometatarsus (PH(F) 7565) (50’ – 55’ / 7’) (prev. id. as *L. mutus*) (Measurements: BP – 7.8).

Distal left tarsometarsus (prev. id as *Lagopus lagopus*) (PH(F) 2934 to 2941). (D/7 – 5’/37-40). (Measurements: BD – 7.76, DD – 8.94).

Left tarsometatarsus, proximal end, shaft and part of distal end (PH(F) 41). (F/7). (prev. id as *Lagopus lagopus* by C.J.O.H.) (Measurements: BP – 8.54, KB – 3.54). (A1).

Right tarsometatarsus, proximal end (PH(F) 299). (F/7). (prev. id as *Lagopus mutus*) (Measurements: BP – 8.04, KB – 3.70). (A1).

Distal left tarsometatarsus (PH(F) 7568 - 7576) (50 – 54’ / 9’ – 10’) (prev. id. as *L. mutus*) (Measurements: BD – 8.4, DD – 8.6).

Distal left tarsometatarsus (PH(F) 7568 - 7576) (50 – 54’ / 9’ – 10’) (prev. id. as *L. mutus*) (Measurements: BD – 8.2, DD – 8.8).

Distal right tarsometatarsus (PH(F) 7568 - 7576) (50 – 54’ / 9’ – 10’) (prev. id. as *L. mutus*) (Measurements: BD – 8.0, DD – 8.59).

Right tarsometatarsus, damaged (PH(F) 634). (8/62 – 70’). (prev. id as *L. mutus* by D. Bramwell). (Measurements: BP – 7.64, KB – 3.07). (A1).

Proximal left tarsometatarsus (PH(F) 963). (8’/62 – 70’). (prev. id as *L. mutus* by D. Bramwell). (Measurements: BP – 7.36, KB – 3.00). (A1).

Proximal Left humerus (PH(F) 56) (A/7’) (prev. id. as *Lagopus* sp.). (Measurements: Bp – 16.24). A1 [Not on original Jacobi list]

PMP Material:

Distal right tarsometatarsus (PH(F) 1165). (“5.6.80” = 5’6’’ - depth too shallow but at 80’ into cave likely is Middle Palaeolithic). (prev. id as *L. mutus* by D. Bramwell). (Measurements: KB – 3.26). (B1).

Distal right tarsometatarsus (PH(F) 8076 to 8082). (9’ – 10’). (prev. id as *Lagopus lagopus* by D. Bramwell). (On bag: from 12 specimens). (Measurements: KB – 3.31, BD – 8.25, DD – 8.61). (C2).

Distal left tarsometatarsus (PH(F) 8076 to 8082). (9’ – 10’). (On bag: from 12 specimens). (prev. id as *Lagopus lagopus* by D. Bramwell). (Measurements: KB – 3.72, BD – 7.47, DD – 8.80). (C2).

Proximal left humerus fragment (PH(F) 80). (12/P). (prev. id as *Lagopus* sp. by D. Bramwell and L. lagopus by C. Harrison). (EP1).

Proximal left humerus fragment (PH(F) 1060). (12/P). (prev. id as *Lagopus* *mutus* by D. Bramwell). (EP1).

Proximal left humerus fragment (No PH no.). (11’ – 12’ / Pc). (On bag: from “a package 6 specimens”). (prev. id as *Lagopus* *lagopus* ? by D. Bramwell). (EP2).

Undet. galliformes

MP Material:

Left carpometacarpus (PH(F) 2934 to 2941). (5’/37-40). (prev. id as *Lagopus lagopus*) (Measurements: GL – 36.82, DD – 7.70).

Left distal humerus (PH(F) 2934 to 2941). (4 – 5 / 47 - 50). (prev. id as *Lagopus lagopus*) (Measurements: TD – 11.62, KT – 5.58).

Left distal humerus (PH(F) 2934 to 2941). (5’/37-40). (prev. id as *Lagopus lagopus*) (Measurements: TD – 10.66, KT – 5.60).

2 furculum symphyses (PH(F) 2934 to 2941). (D/7 – 5’/37-40). (prev. id as *Lagopus lagopus*)

Right carpometacarpus (PH(F) 261). (F/7’). (prev. id as *Lagopus lagopus*) (Measurements: GL – 36.52, TP – 10.58, DD – 7.74, GT – 6.02, HS – 4.78). (A1).

Symphysis of furcula and undet. bone (PH(F) 8059 - 8061). (7’ – 151 - 54). (On bag: from 19 specimens). (prev. id as *L. mutus* by D. Bramwell). (A2).

Distal left femur (PH(F) 1083). (10 / 39). (prev. id as *Lagopus* sp. by D. Bramwell). (Measurements: BD – 9.94, TD – 8.17). (A1).

Proximal left tibiotarsus (PH(F) 13072). (9’ / 65’). (prev. id as *Lagopus lagopus* by D. Bramwell). (Measurements: DP – 11.94, BP – 8.41). (A1).

Distal left ulna (PH(F) 229). (33’/ 7). (prev. id as *Lagopus mutus* by D. Bramwell). (Measurements: DD – 6.55). (A1).

Distal left humerus (PH(F)632) (A/7-8-6”) (prev. id. as *Lagopus muta*). (Measurements: Bd – 10.84). A1 [Not on original Jacobi list]

Proximal right tarsometatarsus (PH(F)9309) (10’-12’/70 from 75 specimens) (prev. id. as *Lagopus muta*). (Measurements: Bp - 7.97, SC – 3.18). A2 [Not on original Jacobi list]

Right coracoid fragment (PH(F)993) (A/7) (Previously id as *Lagopus* sp.). (Measurements: BB – 12.82, BD – 17.28). A1 [Not on original Jacobi list]

PMP Material:

Right proximal femur (PH(F) 39). (P/12’). (prev. id as *Lagopus lagopus*) (Measurements: TP – 10.58, TP - 7.52, BP – 12.78). (EP1).

Left coracoid fragment (PH(F) 7568 – 7573) (50’ – 54’ / 9’ – 10’) (prev. id. as *L. mutus* by D. Bramwell and *L. lagopus* by C.J.O. Harrison).

Distal right humerus (PH(F) 7609 – 7611) (55’ – 9’ 6’ – 7’) (prev. id. as *L. scoticus* and *L. lagopus*). (Measurements: BD– 11.12). (B2).

Left carpometacarpus (PH(F) 7616 – 7617) (F/10’) (prev. id. as *L. scoticus* / *L. lagopus*) (Measurements: GL – 33.79).

Distal right tarsometatarsus Damaged (PH(F) 1210) (D6, at 6’ should be rejected but Jabobi seemed to think it was definitely Middle Palaeolithic) (prev. id. as *L. mutus*). (Measurements: KB – 3.08, BD – 7.72). (R).

Right distal humerus (PH(F) 1343). (12/P). (prev. id. as *Lagopus lagopus*) (No measurements). (EP1).

Distal right humerus fragment (No PH no.). (11’ – 12’ / Pb). (On bag: from “a package 6 specimens”). (prev. id as *Lagopus* *lagopus* ? by D. Bramwell). (EP2).

Distal tibiotarsus (PH(F) 8071 - 8072). (9’ – 10’). (prev. id as *L. mutus* by D. Bramwell). (Measurements: BD – 6.48, DD – 6.32). (On bag: from 12 specimens).

Right scapula (PH(F) 8076 - 8082). (9’ – 10’). (prev. id as *L. mutus* by Colin Harrison). (Measurements: BC - 10.28, KC –4.29). (On bag: from 12 specimens). (C2).

Right carpometacarpus (PH(F) 543). (5/80’, 5’ at 80’’ into cave means it is possibly MP as top layer is very thin here). (prev. id as *Lagopus* sp. by D. Bramwell and *L. lagopus* by C. Harrison). (Measurements: GL – 35.25, TP – 9.97, GT – 5.27, DD – 7.00, HS – 4.45). (B1).

Proximal right femur (PH(F) 8076 to 8082). (9’ – 10’). (On bag: from 12 specimens). (prev. id as *Lagopus lagopus* by D. Bramwell). (Measurements: BP – 6.63, TP –12.20). (C2).

Distal left femur (PH(F) 8076 to 8082). (9’ – 10’). (On bag: from 12 specimens). (prev. id as *Lagopus lagopus* by D. Bramwell). (Measurements: BD – 11.93, TD – 8.83). (C2).

Distal left femur (PH(F) 8076 to 8082) (9’ – 10’) (On bag from 12 specimens). (prev. id as *L. mutus* by C.J.O.H.) (Worn so no measurements). (C2).

Distal left femur (PH(F) 966). (12 / P). (prev. id as *Lagopus* sp. by D. Bramwell). (Measurements: BD – 9.69, TD – 8.35). (EP1).

Distal right tibiotarsus (PH(F) 520). (12 / P). (prev. id as *Lagopus* sp. by D. Bramwell and *Lagopus* *lagopus* by C. Harrison). (Measurements: BD – 7.54, KT – 3.81). (EP1).

Proximal left femur (PH(F) 1327). (8 / CHB). (prev. id as *Lagopus* sp. by D. Bramwell and *L. lagopus* by C. Harrison). (Measurements: TP – 7.25, BP – 12.20). (C1).

Distal right humerus (PH(F) 149). (12/P). (prev. id as *Lagopus* sp. by D. Bramwell and *L. lagopus* by C. Harrison). (Measurements: KT – 5.95, KB – 4.36,TD - 11.19). (EP1).

Distal left ulna (No PH no.). (1’ – 2’ / 35’ – 41’ d, too shallow but Jacobi had it as a maybe). (prev. id as *Lagopus lagopus* ? by D. Bramwell). (Measurements: DD – 8.08, KT – 3.71). (R).

Fragment of pelvis (PH(F) 8270). (11’ – 12’ / P). (On bag: from “a package 6 specimens”). (prev. id as *Lagopus* *mutus* by D. Bramwell and *L. lagopus* by C. Harrison). (Measurements: DR – 4.22). (EP2).

Proximal right tarsometatarsus fragment (PH(F) 9327 – 9330). (11’/P and 12’/P) (from 12 specimens). (prev. id as *Turdus* sp.?). (EP2).

Distal right tibiotarsus (PH(F) 18595). (8/70’). (prev. id as *Corvus monedula*). (Measurements: KT – 3.40, Bd – 7.58, Dd – 7.36). [A1]

Furcula fragment (No PH No.) (11’-12’/Pd “(a) from package 6 specimens”) (Previously id as *Lagopus lagopus?*). EP2 [Not on original Jacobi list]

Gruiformes

*Anthropoides virgo*

MP Material:

Premaxilla (PH(F) 30, 395) (F/9’) (prev. id. as *Anthropoides virgo* by Cowles (1981). (A1). (Photo).

Passeriformes

Alaudidae

MP Material:

Left humerus (PH(F) 8064 to 8068) (7’/51 – 54). (Note on bag: From 19 specimens, 5 bones). (prev. id as *Alauda arvensis*) (Measurements: GL – 24.8, SC – 2.34). (A2). (Photo).

PMP Material:

Left humerus (PH(F) 9323) (11’/P and 12/P) ((Note on bag: From 12 specimens). (prev. id as *Alauda arvensis*) (Measurements: GL – 24.69, SC – 2.11). (EP2).

*Turdus* sp.

PMP Material:

Right carpometcarpus (PH(F) 9069 to 9071) 11 / P (Note on bag: Possibly misidentified) (prev. id as *S. vulgaris*) (Measurements: GL – 20.08, Bp - 5.22, Bd – 4.47). Is *Turdus* i.e. discontinuity in prox. articulation. Size is in lower part of range of *T. merula* and just outside the upper part of the range of *T. philomelos* and *T. iliacus*. (EP2). (Photo).

*Sturnus* sp.

MP Material:

Proximal left humerus (PH(F) 7370) (E 13’ – 15’) (prev. id as *Turdus philomelos*, and subsequently *S. vulgaris* by Stewart 1992) (Measurements: BP – 8.63 – slightly damaged). (A2).

Left carpometacarpus (PH(F) 7372) (E 13’ – 15’) (prev. id as *Turdus philomelos*, and subsequently *S. vulgaris* by Stewart 1992) (Measurements: GL – 20.3, BP – 5.38). (A2).

Left carpometacarpus (PH(F) 7373) (E 13’ – 15’) (prev. id as *Turdus philomelos*, and subsequently *S. vulgaris* by Stewart 1992) (Measurements: GL – 19.26, BP – 4.78). (A2).

Right carpometacarpus (PH(F) 7374) (E 13’ – 15’) (prev. id as *Turdus philomelos*, and subsequently *S. vulgaris* by Stewart 1992) (Measurements: GL - 19.92, BP - 5.1). (A2). (Photo).

Right carpometacarpus (PH(F) 7375) (E 13’ – 15’) (prev. id as *Turdus philomelos*, and subsequently *S. vulgaris* by Stewart 1992) (Measurements: GL – 19.42, BP – 5.2). (A2).

Right carpometacarpus (PH(F) 7376) (E 13’ – 15’) (prev. id as *Turdus philomelos*, and subsequently *S. vulgaris* by Stewart 1992) (Measurements: GL – 20.5, BP - 5.18). (A2).

Left humerus (No PH No.) (A/7) (prev. id. as undet. Aves sp.). (Measurements: GL – 28.42, Bp – 9.05, Bd – Damaged, SC – 2.63). (A1) [Not on original Jacobi list]

PMP Material:

Left humerus (PH(F) 9069 to 9071) (11 / P) (Note on bag: Possibly misidentified) (prev. id as *S. vulgaris*) (Measurements: GL – 26.94, Bp - 9.04, Bd – 6.94, SC – 2.55). (EP1).

Proximal right humerus (PH(F) 9069 to 9071) (11 / P) (Note on bag: Possibly misidentified) (prev. id as *S. vulgaris*) (Measurements: Bp - 9.12). Has hole which appears to be caused by a small carnivore canine. (EP2).

*Turdus* sp./ *Sturnus* sp.

MP Material:

Distal right tibiotarsus (PH(F)7371) (E 13’ – 15’) (prev. id as *Turdus philomelos*) (Measurements: BD – 4.0, DD – 3.98). (A2).

Right ulna (PH(F) 9053 or PH(F)9054 or PH(F)9055 (18i)) (B / 9’) (Measurements: GL – 34.68, BP – 4.9, Did – 4.48). (A1).

Left tarsometatarsus (PH(F)403) (D.9’) (prev. id as *Turdus iliacus*, and subsequently *S. vulgaris* by Stewart 1992) (Measurements: GL – 29.06, BP – 4.42, BD – 3.46, SC – 1.8). (A1).

Left tarsometatarsus (PH(F)140) (E13’ - 15’) (prev. id as *Turdus iliacus*, and subsequently *S. vulgaris* by Stewart 1992) (Measurements: GL – 27.06, BP – 4.42, BD – 3.36, SC – 1.66). (A2).

Proximal left tarsometatarsus (PH(F)1098) (50’ – 55 / 7’) (prev. id. *T. merula*) (Measurements: Bp – 4.6). (A2).

Distal left tibiotarsus (PH(F)501) (50’ – 55 / 7’) (prev. id. *T. merula*) (Measurements: Bd – 4.2, Dd – 4.27). (A2).

Right coracoid (PH(F) 8054) (7’ / 51 – 54) (On bag: from 19 specimens) (prev. id as *T. merula*?) (Measurements: GL – 28.64 , Bp – 27.41, BF – 6.19). (A2).

Left tarsometatarsus (PH(F) 8070) (7’ / 51 – 54) (Note on bag: from 19 specimens) (prev. id as *Sturnus vulgaris*) (Measurements: GL – 29.48, Bp – 4.45, Bd – 3.47, SC – 1.66). (A2).

PMP Material:

Right ulna (No Ph number) (11’ – 12’ / P) (Note on bag: “(a) from package 6 specimens”) (prev, id as *Turdus pilaris*) (Measurements: GL - 37.49, Bp - 5.29, Bd - 4.74). If is a member of *Turdus* it is in the size range of *T. pilaris*, just below the size range of *T. viscivorus* and within the range of the three *T. torquatus*. (EP2).

Proximal left tarsometatarsus (PH(F) 9327 – 9330) (11’ / P and 12 / P) (On bag: from 12 specimens) (prev. id as *Turdus* sp.) (Measurements: Bp – 4.36). (EP2).

Left scapula (PH(F) 9327 – 9330) (11’/P and 12’/P). (On bag: from 12 specimens) (prev. id. a *Turdus* sp. by Bramwell). (EP2)

Left tibiotarsus (PH(F) 9053 or PH(F) 9054 or PH(F) 9055 (17i)) (12/P?) (prev. id. as *Turdus merula*?). (Measurements: GL – 47.74, Bp – 7.0, Bd – 3.72, Dd – 3.76). (EP1?) [Not on original Jacobi list]

Left ulna (PH(F) 9325 (11’/P and 12/P from 25 specimen) (prev. id. as *Turdus philomelos*?). (Measurements: GL – 32.56, Bp – 4.19, Did – 4.4). (EP2) [Not on original Jacobi list]

*Corvus corax*

MP Material:

Os premaxillare (PH(F) 1044) (63’ / 15’) (131) (prev. id as *Corvus corax*) (No measurements). (A1).

Distal right radius (PH(F) 639) (50’ – 54’ / 9’ – 10’; pencil mark on bone in match box 8’) (131) (prev. id as *Corvus corax*) (Measurements: f – 8.56). (A1).

Distal left ulna (PH (F) 1122) (D9’) (Measurements: f - 11.9; g – 9.98). (A1).

Left tibiotarsus (No PH No.) (7’ / 64b) (Measurements: a – 110.1+; d – 18.56; f- 6.1). Articulations are gnawed? Bone is grainey so is immature. (prev. id as *Corvus corax*). (A1).

Distal left tibiotarsus (PH(F) 1119 or PH(F) 6) (F. 10’) (Measurements: h – 11.72, i – 12.16, j – 13.03). (A1).

Distal left tarsometatarsus (PH(F) 1119 or PH(F) 6) (F10’; on bone in pencil 10’ / 60’) (Measurements: d – 10.2; e – 6.2; f – 4.4; c – 5.64+/-). (A1).

Distal left carpometacarpus - described as juvenile on bag but no sign of this (No PH no.). (A/7’6”). (prev. id as *Corvus corax*). (A1).

Right coracoid fragment (Ph(F) 7840). (A/7’). (prev. id as *Corvus corax* by D. Bramwell, Mar. 1970). (A1).

Distal humerus - juvenile as shaft is grainey and articulation is unformed (prev. id as *Corvus corax*). (No PH no.). (7’/64 a). (A1).

PMP Material:

Proximal left ulna (No PH No.) (12P). Shaft is gnawed. Bone is grainey so is immature. (prev. id as *Corvus corax*). (Measurements: Bp – 13.34, Dip - 16.16, SC – 6.18). (EP1).

Right carpometacarpus (PH(F) 13075 to PH(F) 13076). (12/P) (prev. id as *Corvus corax*). (Measurements: GL – 67.46, BP – 16.18, Bd – 15.86) (EP1). (Photo).

Distal tibiotarsus – no articulations and is very grainey (PH(F) 13075 to PH(F) 13076). (No code). (prev. id as *Corvus corax*). (EP1).

Distal left femur - juvenile as shaft is grainey (No PH no.). (P 11’ – 12’). Gnawed. (prev. id as *Corvus corax*). (Measurements: Bd – 14.39, SC – 6.23). (EP1).

*Corvus* cf. *monedula*

MP Material:

Right femur (PH(F) 8069). (7’/51 – 54). (On bag: from 19 specimens). (prev. id as *Corvus monedula*). (Measurements: GL – 36.99, Bp - 6.94, Dp – 4.12, Bd – 7.20, SC – 3.09). (A2). (Photo).

Undet. smaller corvidae

PMP Material:

Left coracoid – immature as proximal end is unformed and grainey (PH(F) 9326). (11’/P and 12’/P). (On bag: from 12 specimens). (prev. id as *Pica pica*). (Measurements: GL – 31.49, SC – 2.22). (EP2).

Passeriformes - Family, Genus and species unknown

MP Material:

Right humerus – immature (PH(F) 7628) (E13’ – 15’) (prev. id. as young plover and young great grey shrike *Lanius excubitor*) (Measurements: GL – 25.63, SC – 2.45). (A2).

Left femur (in 2 frags) (PH(F) 9313). (10’ - 12’/70). (On bag – from 75 specimens). (prev. id. a *Passer domesticus* by D. Bramwell and *Carduelis chloris* by CJOH). (A2).

Right coracoid (PH(F) 9310). (10’ - 12’/70). (On bag – from 75 specimens). (prev. id. As *Alauda arvensis*). (A2).

Right ulna (PH(F) 9311 PH(F) 9312). (10’-12’/70). (On bag – from 75 specimens). (prev. id. as *Alauda arvensis*). (A2).

Proximal right ulna (PH(F) 9311 PH(F) 9312). (10’-12’/70). (On bag – from 75 specimens). (prev. id. as *Alauda arvensis*). (A2).

Left ulna (PH(F) 832). (50’-55’/7’) 97 (prev. id. as *Carduelis canabina*). (A2).

Left ulna (PH(F) 8064 – PH(F) 8068). (7’/51 – 54). (On bag – from 19 specimens). (prev. id. as *Alauda arvensis* by D. Bramwell). (A2).

Left coracoid (PH(F) 8064 – PH(F) 8068). (7’/51 – 54). (On bag – from 19 specimens(prev. id. as *Alauda arvensis* by D. Bramwell). (A2).

Left tibiotarsus (PH(F) 8064 – PH(F) 8068). (7’/51 – 54). (On bag – from 19 specimens) (prev. id. as *Alauda arvensis* by D. Bramwell). (A2).

Proximal right femur (PH(F) 8064 – PH(F) 8068). (7’/51 – 54). (On bag – from 19 specimens). (prev. id. as *Alauda arvensis* by D. Bramwell). (A2). [Not on original Jacobi list]

Sternum fragment, has possible avian predator/scavenger damage) (PH(F)446) (A/9) (prev. id. as undet. Aves sp.). A1 [Not on original Jacobi list]

Furcula (No PH No.) (9’-10’) (Previously id as *Turdus philomelos?*) (D.W. Yalden – “morphology wrong for redwing; too small for blackbird”). A2 [Not on original Jacobi list]

PMP Material:

Left ulna (PH(F)9324) (11’/P and 12’/P from 12 specimens) (prev. id. as *Fringilla coelebs*?). (Measurements: GL – 20.99, Bp – 2.91, Did – 2.56). EP2 [Not on original Jacobi list]

Left ulna (Ph(F) 8055 – 8058) (7’ / 51’ – 54) (prev. id. as Finch / Bunting / Undetermined). B2

Distal left ulna (Ph(F) 8055 – 8058) (7’ / 51’ – 54) (prev. id. as Finch / Bunting / Undetermined). B2 [Not on original Jacobi list]

Distal tibiotarsus (cant be sided as articulation covered in sediment) (Ph(F) 8055 – 8058) (7’ / 51’ – 54) (prev. id. as Finch / Bunting / Undetermined). B2 [Not on original Jacobi list]

Aves - Family, Genus and species unknown

MP Material:

Distal left radius, immature (PH(F)284) (E/7’) (prev. id. as undet. Aves sp.). (A1).

Cervical vertebra (large bird) (PH(F) 43) (E/10) (prev. id. as undet Aves sp.). (A1).

Fragment of synsacrum (PH(F) 12554) (E/13’ – 15) (prev. id. as undet. Aves sp.). (A2).

Phalange of pes (PH(F)8053) (7’/51-54) (Previously id as *Lagopus lagopus* by Bramwell? and indet. by C.J.O. Harrison). A2

5 cranial fragments (apparently belonging together and not recognized as belonging to same bone) (PH(F) 12581 – 12585) (8 / 6?) (Previously id as Undetermined). A1 [Not on original Jacobi list]

Synsacrum (PH(F) 12581 – 12585) (8 / 62) (Previously id as Undetermined). A1 [Not on original Jacobi list]

PMP Material:

Proximal radius (PH(F)150) (12/P) (prev. id. as undet. Aves sp.). EP1 [Not on original Jacobi list]

Phalange of pes (PH(F) 167) (E / 13) (Previously id as Undetermined). C1 [Not on original Jacobi list]

Phalange of pes (PH(F) 1224) (E / 13) (Previously id as Undetermined). C1 [Not on original Jacobi list]

Vertebra fragment (Ph(F) 8055 – 8058) (7’ / 51’ – 54) (prev. id. as Finch / Bunting / Undetermined). B2 [Not on original Jacobi list]

2 thoracic vertebrae (No PH no.) (13’ – 16’) ) (Previously id as Undetermined). C2 [Not on original Jacobi list]

Phalange of manus No PH no.) (13’ – 16’) ) (Previously id as Undetermined). C2 [Not on original Jacobi list]

Text D Measurements of #MR40 (female mallard *Anas platyrhynchos*) in author (JS) collection

Specimen used to transforms all the Pin Hole duck measurements to a percentage of an arbitrary mallard *Anas platyrhynchos* (Fig. A).

It may be that the way the measurements were taken of #MR40 and the Pin Hole specimens of the distal tarsometatarsus (Bd) are different to those defined in Bacher [49].This would explain why the tarsometatarsi of the female mallard #MR40 are smaller than all mallard measured by Bacher.

Humerus

GL – 90.82

Bp – 20.96

SC – 6.7

Bd – 14.64

Ulna

GL – 76.38

Bp – 9.88

SC – 5.06

Did – 10.26

Radius

GL – 69.6

Bp – 4.78

Bd – 6.12

Carpometacarpus

GL – 56.44

Bp – 12.82

Bd – 6.96

Coracoid

GL – 52.5

Lm – 47.02

BF – 20.18

Scapula

Dic – 1.68

Femur

GL – 48.38

Bp – 10.88

Bd – 10.68

Dd – 8.04

Tibiotarsus

GL – 76.98

Bp –8.74

Dip –12.4

Bd – 8.4

Dd – 8.6

SC – 3.72

Tarsometarsus

GL – 43.28

Bp – 9.03

SC – 4.02

Bd – 8.95

References

1. Armstrong, A.L. 1925. Excavations at Mother Grundy’s Parlour, Creswell Crags, Derbyshire, 1924. *Journal of the Royal Anthropological Institute*, 55: 146-178.

2. Armstrong, A.L. 1926. Excavations at Creswell Crags, Derbyshire. 1924-1926. *Transactions of the Hunter Archaeological Society*, 3(2): 116-122.

3. Armstrong, A.L. 1932. Excavations in the Pin Hole Cave, Creswell Crags, Derbyshire. *Proceedings of the Prehistoric Society of East Anglia*, 6(4) for 1931: 330-334.

4. Armstrong, A.L. 1939. Palaeolithic Man in the North Midlands. *Memoirs and Proceedings of the Manchester Literary and Philosophical Society*, 83 for 1938-39: 87-116.

5. Armstrong, A.L. 1956. Palaeolithic, Neolithic and Bronze Ages. In Linton, D.L. (ed.) *Sheffield and its Region: a Scientific and Historical Survey*, 90-110. London: British Association for the Advancement of Science.

6. Mello, J.M. 1875. On some bone-caves in Creswell Crags. *Quarterly Journal of the Geological Society of London*, 31(4), 679-691.

7. Mello, J.M. 1876. The bone-caves of Creswell Crags – 2nd paper. *Quarterly Journal of the Geological Society of London*, 32(3), 240-244.

8. Collcutt, S.N. 1975. *The Stratigraphy of Creswell Crags*. Unpublished M.A. Thesis, University of Edinburgh.

9. Jenkinson, R.D.S. 1984. *Creswell Crags. Late Pleistocene Sites in the East Midlands*. Oxford: British Archaeological Report (British Series) 122.

10. Jacobi, R.M., Rowe, P.J., Gilmour, M.A., Grün, R. and Atkinson, T.C. 1998. Radiometric dating of the Middle Palaeolithic tool industry and associated fauna of Pin Hole Cave, Creswell Crags, England. *Journal of Quaternary Science*, 13(1), 29-42.

11. Jacobi, R.M., Higham, T.F.G. and Bronk Ramsey, C. 2006. AMS radiocarbon dating of Middle and Upper Palaeolithic bone in the British Isles: improved reliability using ultrafiltration. *Journal of Quaternary Science*, 21(5), 557-573.

12. Currant, A.P. and Jacobi, R.M. 2001. A formal biostratigraphy for the Late Pleistocene of Britain. *Quaternary Science Reviews*, 20(16-17), 1707-1716.

13. Yalden, D.W. 1999. *The History of British Mammals*. London: T.& A.D. Poyser.

14. Stewart, J.R., van Kolfschoten, M., Markova, A. and Musil, R. 2003. The Mammalian Faunas of Europe during Oxygen Isotope Stage Three. Chapter 7. In: T. H. van Andel and S.W. Davies (Eds.). *Neanderthals and Modern Humans in the European Landscape during the Last Glaciation, 60,000 to 20,000 years ago: Archaeological Results of the Stage 3 Project*. McDonald Institute Monograph Series, pp. 103 - 129.

15. Stewart, J. R. and Parfitt, S.A., 2011. [Late Quatermary environmental change at Walou Cave: evidence from a preliminary analysis of the small mammals.](http://eprints.bournemouth.ac.uk/17717/) In: Draily, C., Pirson, S. and Toussaint, M. (eds.) *La Grotte Walou a Trooz (Belgique).* Namur, Belgium: Departement du Patrimoine et l'Institut du Patrimoine Wallon, pp. 38-59.

16. Cramp, S. 1985. *Handbook of the birds of Europe, the Middle East and North Africa: the birds of the Western Palaearctic. Vol. 4. Terns to Woodpeckers*. Oxford University Press, Oxford.

17. Cramp, S. and Simmons, K.E.L. 1980. *Handbook of the birds of Europe, the Middle East and North Africa: the birds of the Western Palaearctic. Vol. 2. Hawks to Bustards*. Oxford University Press, Oxford.

18. Cramp, S and Perrins, C. 1994. *Handbook of the birds of Europe, the Middle East and North Africa: the birds of the Western Palaearctic. Vol. 8. Crows to Finches*. Oxford University Press, Oxford.

19. Harrison, C.J.O. 1982. *An Atlas of the Birds of the Western Palaearctic*. Collins, London.

20. Gilbertson D.D. and Jenkinson R.D.S. (Eds.). *In the Shadow of Extinction: a Quaternary Archaeology and Palaeoecology of the Lake, Fissures and smaller Caves at Cresswell Crags SSSI.* University of Sheffield - John Collis - Publisher.

21. [Fieller,](http://www.sciencedirect.com/science/article/pii/0305440392900455) N.R.J., [Gilbertson](http://www.sciencedirect.com/science/article/pii/0305440392900455), D.D., [Griffin](http://www.sciencedirect.com/science/article/pii/0305440392900455), C.M., [Briggs](http://www.sciencedirect.com/science/article/pii/0305440392900455), D.J. and [Jenkinson, R.D.S.](http://www.sciencedirect.com/science/article/pii/0305440392900455)  1992. The statistical modelling of the grain size distributions of cave sediments using log skew laplace distributions: Creswell Crags, near Sheffield, England. [*Journal of Archaeological Scienc*e](http://www.sciencedirect.com/science/journal/03054403) [19(2](http://www.sciencedirect.com/science/journal/03054403/19/2)): 129–150.

22. Macdonald, D. and Barrett, P. 1993. *Mammals of Britain and Europe*. Harper Collins publishers, London.

23. Finlayson, C., Brown, K., Blasco, R., Rosell, J., Negro, J.J., Bortolotti, G.R., et al. 2012. Birds of a Feather: Neanderthal Exploitation of Raptors and Corvids. *PLoS ONE* 7(9): e45927. doi:10.1371/journal.pone.0045927.

24. Blasco, R., Fernández Peris, J., 2009. Middle Pleistocene bird consumption at level XI of Bolomor Cave (Valencia, Spain). *Journal of Archaeological Science* 36, 2213-2223.

25. Blasco, R., Rosell, J., Fernández Peris, J., Arsuaga, J.L., Bermúdez de Castro, J.M., Carbonell, E. 2013. Environmental availability, behavioural diversity and diet: a zooarchaeological approach from the TD10-1 sublevel of Gran Dolina (Sierra de Atapuerca, Burgos, Spain) and Bolomor Cave (Valencia, Spain). *Quaternary Science Reviews* 70, 124-144.

26. Blasco, R., Finlayson, C., Rosell, J., Sánchez Marco, A., Finlayson, S., Finlayson, G., et al. 2014. The earliest pigeon fanciers. *Scientific Reports* 4: 5971 DOI: 10.1038/srep05971

27. Cochard, D., Brugal, J.-Ph., Morin, E., Meignen, L., 2012. Evidence of small fast game exploitation in the Middle Paleolithic of Les Canalettes (Aveyron, France). *Quaternary International* 264, 32-51.

28. Hardy, B.L., Moncel, M.-H., 2011. Neanderthal use of fish, mammals, birds, starchy plants and wood 125-250,000 years ago. *PLoS ONE* 6 (8), e23768.

29. Hardy, B.L., Moncel, M.-H., Daujeard, C., Fernandes, P., Béarez, Ph., Desclaux, E., et al. 2013. Impossible Neanderthals? Making string, throwing projectiles and catching small game during Marine Isotope Stage 4 (Abri du Maras, France). *Quaternary Science Reviews* 82, 23-40

30. Morin, E., Laroulandie, V., 2012. Presumed symbolic use of diurnal raptors by Neanderthals. *PLoS ONE* 7, e32856.

31. Romandini, M., Peresani, M., Laroulandie, V., Metz, L., Pastoors, A., Vasquero, M., et al. 2014. Convergent Evidence of Eagle Talons Used by Late Neanderthals in Europe: A Further Assessment on Symbolism. *PLoS ONE* 9(7): e101278. doi:10.1371/journal.pone.0101278

32. Peresani, M., Fiore, I., Gala, M., Romandini, M., Tagliacozzo, A., 2011. Late Neandertals and the intentional removal of feathers as evidenced from bird bone taphonomy at Fumane Cave 44 ky B.P., Italy. *Proceedings of the National Academy of Sciences* 108, 3888e3893.

33. Bello, S.M., Soligo, C., 2008. A new method for the quantitative analysis of cutmark micromorphology. *Journal of Archaeological Science* 35, 1542-1552.

34. Lorenc, M. 2006. On the taphonomic origins of Vistulian bird remains from cave deposits in Poland. *Acta Zoologica Cracoviensa* 49A(1–2): 63–82.

35. Bang, P. and Dahlström, P. 1974. *Collins guide to animal tracks and signs*. Collins, London.

36. Stewart, J.R. 1992. *The Turdidae of Pin Hole Cave, Derbyshire*. Unpublished MSc. Dissertation, City of London, North London and Thames Polytechnics.

37. Mourer-Chauviré, C. 1975. Les Oiseaux du Pleistocene Moyen et Superieur de France. *Documents de Laboratoire de Geologie de la Faculte de Lyon* No. 64, 2 Fasc., 624pp.

38. Stewart, J.R. 2010. [The bird remains from the West Runton Freshwater Bed Norfolk, England.](http://eprints.bournemouth.ac.uk/16929/) *Quaternary International*, 228: 72-90.

39. Harrison, C.J.O. and Stewart, J.R. 1999. The birds remains. In: M.B. Roberts and S.A. Parfitt (Eds.). The *Middle Pleistocene Site at ARC Eartham Quarry, Boxgrove, West Sussex, U.K*. English Heritage Monograph Series 16, London.

40. Livingston, S.D. 1989. The taphonomic interpretation of avian skeletal part frequencies. *Journal of Archaeological Science*, 16: 537 - 547.

41. Ericson, P.G.P. 1987. Interpretation of archaeological bird remains: a taphonomic approach. *Journal of Archaeological Science*, 14: 65 - 75.

42. Mourer-Chauviré, C. 1983. Les oiseaux dans les habitats palaeolithiques: gibier des hommes ou proies des rapaces? In: C. Grigson and J. Clutton-Brock (Eds.). *Animals in Archaeology, Tome II. Shell Middens, Fishes and Birds*. BAR International Series 183, Oxford. 111 - 124.

43. Baales, M. 1992. Accumulation of bones of *Lagopus* in Late Pleistocene sediments. Are they caused by man or animals? *Cranium* 9(1): 17 - 22.

44. Stiner MC. 2001. Thirty years on the ‘Broad Spectrum Revolution’ and Paleolithic demography. *Proceedings of the National Academy of Sciences* 98(13): 6993–6996.

45. Stewart, J.R. 2004. Neanderthal-Modern Human Competition?: A comparison between the mammals associated with Middle and Upper Palaeolithic industries in Europe during OIS 3. *International Journal of Osteoarchaeology* 14: 178 – 189.

46. Fa, J.E., Stewart, J.R., Lloveras, L., Vargasa J.M. 2013. Rabbits and hominin survival in Iberia. *Journal of Human Evolution* [64(4](http://www.sciencedirect.com/science/journal/00472484/64/4)), 233–241.

47. Kellner, M. 1986. Vergleichend morphologische Untersuchungen an Einzelknochen des Postkranialen Skeletts in Europa Vorkommender Ardeidae, Ludwig-Maximilians-Universität, München.

48. Gruber, A. 1990. *Vergleichend morphologische Untersuchungen an Einzelknochen in Ägypten Vorkommender Ciconiidae*. Ludwig-Maximilians-Universität, München.

49. Bacher, A. 1967. *Vergleichend morphologische Untersuchungen an Einzelknochen des postkranial Skeletts in Mitteleuropa vorkommender Schwäne und Gänse*. Ludwig-Maximilians-Universität, München.

50. Woelfle, E. 1967. *Vergleichend morphologische Untersuchungen an Einzelknochen des postcranialen Skeletts in Mitteleuropa vorkommender Enten, Halbgänse und Säger*. Ludwig-Maximilians-Universitat, Munchen.

51. Solti, B. 1996. The Comparative osteomorphological study of the European small-statured falcon (Aves: Falconidae). *Folia Historico Naturalia Musei Matraensis* 21: 5 – 282.

52. Fick, O.K.W. 1974. *Vergleichend morphologische Untersuchungen an Einzelknochen europäischer Taubenarten*. Ludwig-Maximilians-Universität, München.

53. Langer, G. 1980. *Vergleichend morphologische Untersuchungen an Einzelknochen in Mitteleuropa vorkommender mittelgrosse Eulenarten*. Ludwig-Maximilans-Universität, München.

54. Stewart, J.R. In press. The Bird Remains from the 1995/96 Season (the Hominid Area), Boxgrove, West Sussex. In: M.B. Roberts and S.A. Parfitt (Eds.). *The Boxgrove Hominid Site*. English Heritage Monograph Series, London.

**Table A. Measurements of *Ardea* sp. humerus shaft probably** from Pin Hole MIS 3 level PH(F) 5895 A in relation to those of those of modern Ardeid specimens [47] .

|  | **Humerus KC** |
| --- | --- |
| PH(F) 5895 A | 10.28 |
| *Ardea cinerea* | 9.1 – 11.0 (N = 41) |
| *Ardea pupurea* | 7.0 – 8.0 (N = 24) |
| *Botaurus stelaris* | 6.8 – 8.1 (N = 10) |
| *Casmodius albus* | 7.9 – 10.0 (N = 11) |
| *Egretta garzetta* | 5.4 – 6.5 (N = 13) |
| *Ardeola ralloides* | 4.1 – 5.3 (N = 15) |
| *Bubulcus ibis* | 5.0 – 6.1 (N = 29) |

**Table B. Measurements of Pin Hole MIS 3 level *Ciconia* sp. coracoid PH(F) 5896 in relation to those of those of modern European specimens [48**].

|  | **Coracoid LM** |
| --- | --- |
| PH(F) 5896 | 73.94 |
| *Ciconia ciconia* | 66.0 – 78.5 |
| *C. nigra* | 67.0 – 76.2 |

**Table C. Measurements of large Anseriformes humeri and coracoidea from MIS 3 of Pin Hole together with comparative measurements [49,50**].

|  | **Humerus** | | | | **Coracoid** | | |
| --- | --- | --- | --- | --- | --- | --- | --- |
|  | **GL** | **BP** | **BD** | **KT [= SC]** | **GL** | **LM** | **BB** |
| L humerus  PH(F) 18543 | 123.9 | 26.41 | 17.54 | 8.62 |  |  |  |
| Distal L humerus  PH(F) 13082 | - | - | 16.18 | - |  |  |  |
| R coracoid  PH(F) 5895B |  |  |  |  | 67.2. | 60.12 | 25.8 |
| R coracoid  PH(F) 13083 |  |  |  |  | 62.06 | 54.9 | 24.7 |
| R coracoid  PH(F) 18540 |  |  |  |  | 56.18 | 49.76 | 21.22 |
| *Anser anser* | 151.7 – 184.9  (N=96) | 30.6 – 37.8  (N=96) | 22.5 – 27.0  (N=96) | 8.1 – 10.4  (N=96) | 67.8 – 84.1  (N=102) | 59.2 – 73.1  (N=102) | 25.5 – 33.3  (N=102) |
| *A. fabalis* | 152.2 – 178.4  (N=54) | 30.7 – 37.7  (N=54) | 22.6 – 27.8  (N=54) | 8.1 – 10.8  (N=54) | 67.4 – 84.2  (N=62) | 58.9 – 72.6  (N=62) | 25.3 – 33.8  (N=62) |
| *A. brachyrhynchos* | 143.0 – 165.2  (N=44) | 30.7 – 35.5  (N=44) | 22.5 – 25.4  (N=44) | 7.8 – 9.7  (N=44) | 63.0 – 79.8  (N=50) | 56.3 – 74.6  (N=50) | 23.7 – 33.0  (N=50) |
| *A. albifrons* | 139.5 – 159.1 (N=48) | 29.0 – 32.7  (N=48) | 21.0 – 24.4  (N=48) | 7.7 – 9.1  (N=48) | 61.9 – 71.2  (N=48) | 53.5 – 61.7  (N=48) | 23.5 – 28.9  (N=48) |
| *Branta leucopsis* | 133.7 – 180.9  (N=35) | 27.2 – 31.0 (N=35) | 19.7 – 22.1 (N=35) | 7.1 – 8.7 (N=35) | 59.6 – 67.3 (N=38) | 50.4 – 58.4 (N=38) | 22.3 – 29.2 (N=38) |
| *B. bernicla* | 116.2 – 131.6  (N=56) | 23.7 – 27.0  (N=56) | 16.4 – 19.9  (N=56) | 6.1 – 7.5  (N=56) | 50.7 – 59.3  (N=60) | 44.7 – 57.1  (N=60) | 19.1 – 23.5  (N=60) |
| *Tadorna tadorna* | 89.8 – 111.5 (N=38) | 19.9 – 24.0 (N=39) | 13.7 – 17.0 (N=37) | 6.7 – 8.8 (N=39) | 48.5 – 59.5 (N=44) | 40.0 – 52.9 (N=44) | 19.5 – 24.3 (N=44) |
| *Somateria mollissima* | 98.2 – 121.3 (N=60) | 21.9 – 27.8 (N=61) | 14.7 – 17.4 (N=61) | 6.0 – 8.1 (N=61) | 59.2 – 73.9 (N=83) | 54.8 – 68.2 (N=83) | 23.1 – 29.6 (N=84) |

**Table D. Measurements of *Melanita* spp. humeri and tarsometatarsi from the Middle Palaeolithic of Pin Hole together with comparative measurements [49,50].**

|  | **Humerus** | | | | **Tarsometatarsus** | | | |
| --- | --- | --- | --- | --- | --- | --- | --- | --- |
|  | **GL** | **BP** | **BD** | **KS [= SC]** | **GL** | **BP** | **BD** | **SC** |
| L humerus  PH(F) 7805 | 96.09 | 21.08 | 12.0 | 5.84 |  |  |  |  |
| L Tarsometatarsus  PH(F) 13084 |  |  |  |  | 43.33 | 10.38 | 10.28 | 4.21 |
| *Melanitta fusca* | 93.2 – 103.3 (26) | 20.2 – 22.9 (26) | 13.6 – 15.0 (26) | 6.2 – 7.5 (26) | 44.6 – 50.8 (24) | 10.2 – 11.9 (23) | 9.2 – 11.9 (24) | 4.0 – 5.4 (24) |
| *Melanitta nigra* | 86.1 – 98.2 (38) | 17.3 – 20.7 (40) | 11.7 – 13.3 (39) | 5.4 – 6.4 (39) | 41.4 – 50.2 (42) | 9.5 – 10.9 (42) | 9.0 – 11.2 (42) | 3.8 – 5.2 (42) |

**Table E. Measurements of Pin Hole MIS 3 small *Falco* remains and modern comparative material. Included are the present author’s measurements, taken on specimens from the Natural History Museum’s Collections at Tring, together with those in brackets from Solti [51**].

|  | **Humerus** | | | | **Carpometacarpus** | | | **Tibiotarsus** | | | | **Tarsometatarsus** | |
| --- | --- | --- | --- | --- | --- | --- | --- | --- | --- | --- | --- | --- | --- |
|  | **GL** | **Bp** | **Bd** | **SC** | **GL** | **Bp** | **Bd** | **GL** | **Bd** | **Dd** | **SC** | **Bp** | **SC** |
| PH(F) 2614 |  |  |  |  | 38.22 | 9.59 | 6.06 |  |  |  |  |  |  |
| PH(F) 18548 | 50.74 | 10.66 | 8.64 | 4.27 |  |  |  |  |  |  |  |  |  |
| PH(F) 18545 |  |  |  |  |  |  |  |  | 7.19 | 5.21 |  |  |  |
| PH(F) 8269 |  |  |  |  |  |  |  | 61.73 | 6.89 | 5.49 | 3.08 |  |  |
| PH(F) 9307 |  |  |  |  |  |  |  |  |  |  |  | 6.57 | 3.23 |
| *Falco tinnunculus* | 50.4 – 57.26 N=21 (50.3 – 58.4 N=54) | 11.42 – 13.1 N=21 (10.7 – 12.7 N=56) | 8.9 – 10.06 N=21 (8.7 – 10.3 N=55) | 3.72 – 4.7 N=21 (4 – 4.9 N=57) | 32.6 – 37.4 N=21 (33.1 – 39.3 N=52) | 8.9 – 10.14 N=21 (8.2 – 10.2 N=52) | 5.5 – 6.42 N=21 (5.5 – 6.6 N=52) | 56.28 – 64.32 N=21 (55.7 – 63.4 N=52) | 6.04 – 7.3 N=21 (6.1 – 7.5 N=52) | 5.06 – 5.88 N=21 (5 – 6.1 N=52) | 2.9 – 3.42 N=21 (2.8 – 3.5 N=52) | 6.24 – 7.46 N=19  (6.6 – 8 N=51) | 2 – 3.5 N=19  (2.6 – 3.4 N=51) |
| *F. columbarius* | 44.04 – 51.8 N=11  (43.8 – 54 N=38) | 10.98 – 13.3 N=11  (10.3 – 12.9 N=39) | 8.6 – 10.12 N=11  (8 – 10.3 N=38) | 4.12 – 4.6 N=11  (3.9 – 4.6 N=38) | 30.02 – 35.1 N=11  (29.6 – 36.6 N=38) | 8.4 – 9.72 N=11  (8.2 – 10 N=39) | 5.3 – 5.82 N=11  (5.3 –6.3 N=40) | 52.36 – 61.6 N=11  (51.8 – 61.2 N=37) | 5.9 – 6.9 N=11 (6.1 – 7.6 N=38) | 4.78 – 5.5 N=11 (4.8 – 6 N=38) | 2.4 – 3.08 N=11  (2.5 – 3.2 N=36) | 5.68 – 6.64 N=11  (6.3 – 7.9 N=39 | 2.26 – 2.8 N=11 (2.2 – 2.9 N=39) |
| *F. subbuteo* | 51.56 – 58 N=5  (51.3 – 60.2 N=17) | 11.62 – 13.9 N=6  (11.6 – 14.6 N=20) | 9.04 – 10 N=5  (9.9 – 11.4 N=19) | 4.28 – 4.52 N=5  (4.5 – 5.2 N=19) | 35.06 – 40.82 N=7  (34.9 – 40.2 N=18) | 9.2 – 10.8 N=6  (10 – 11.5 N=18) | 6.06 – 6.7 N=7  (6.2 – 7.4 N=18) | 53.52 – 60.2 N=7  (54.2 – 60.6 N=16) | 6.16 – 7.1 N=7  (6.5 – 8 N=17) | 4.68 – 5.8 N=7 (4.7 – 5.7 N=17) | 2.66 – 3.08 N=7  (2.7 – 3.7 N=17) | 5.8 – 6.94 N=6  (6.5 – 8.3 N=18) | 2.2 – 2.66 N=6  (2.4 – 2.9 N=18) |
| *F. vespertinus* | 46.02 – 50.84 N=4  (48 – 53.7 N=31) | 10.44 – 11.7 N=4  (9.8 – 11.7 N=31) | 8.2 – 9.02 N=4  (8.2 – 9.4 N=31) | 3.68 – 4.1 N=4  (3.5 – 4.2 N=31) | 31.34 – 35.1 N=4  (31.4 – 34.6 N=31) | 8.8 – 9.2 N=4  (8.3 – 9.6 N=31) | 5.4 – 6.08 N=4  (5.1 – 6.1 N=31) | 48.2 – 54.6 N=4  (45.6 – 52 N=30) | 5.3 – 6.24 N=4  (5.3 – 6.6 N=31) | 4.42 – 4.8 N=4  (4.2 – 5 N=31) | 2.4 – 2.8 N=4  (2.3 – 2.9 N=31) | 5.7 – 6.12 N=4  (5.6 – 6.5 N=31 | 2.2 – 2.4 N=4  (2.2 – 2.7 N=31 |
| *F. naumanni* | 47.9, 51.58 (47.7 – 52 N=5) | 10.2, 11.4  (9.5 – 10 N=4 | 7.96, 8.84  (8.2 – 8.6 N=4 | 3.48, 3.94  (3.5 – 4.1 N=4) | 31.88, 34.02  (31.7 – 32 N=4) | 8.42, 8.8  (8.0 – 8.4 N=4) | 5.42, 5.8  (5.3 – 5.7 N=4) | 46.6, 51.04  (46 – 50 N=4) | 5.42, 5.9  (5.8 – 6.2 N=4) | 4.6, 4.62  (4.7 – 4.9 N=3) | 2.4, 2.62  (2.5 – 3 N=4) | 5.44, 5.54  (6 – 6.4 N=4 | 2.12, 2.16  (2.2 – 2.6 N=4) |

**Table F. Measurements of Pin Hole MIS 3 carpometacarpus PH(F) 2155 in relation to those of modern specimens of Columbidae [52**] and Pteroclidae (authors measurements).

|  | **Carpometacarpus** | | | | |
| --- | --- | --- | --- | --- | --- |
|  | **GL** | **BP** | **DD** | **BS** | **KB** |
| PH(F) 2155 | 40.34 | 11.68 | 6.68 | 8.94 | 3.40 |
| *Columba palumbus* | 36.1 – 40.3 (24) | 10.5 – 11.9 (24) | 6.1 – 7.1 (24) | 7.9 – 8.9 (24) | 2.6 – 3.3 (24) |
| *C. livia* | 30.8 – 33.0 (8) | 8.6 – 9.7 (8) | 5.3 – 5.6 (8) | 6.1 – 6.9 (8) | 2.4 – 2.8 (8) |
| *C. oenas* | 31.8 – 35.3 (18) | 8.9 – 9.8 (18) | 5.0 – 5.8 (18) | 6.4 – 7.0 (18) | 2.3 – 2.7 (18) |
| *Streptopelia turtur* | 22.5 – 26.5  (15) | 6.8 – 7.7 (15) | 3.9 – 4.5 (15) | 5 – 5.9 (15) | 1.8 – 2.1 (15) |
| *S. decaocto* | 25.6 – 28.3  (55) | 7.4 – 8.4 (55) | 4.1 – 5 (55) | 5.4 – 6.5 (55) | 1.9 – 2.3 (55) |
| *Pterocles alchata* | 30.2 – 33.04 (4) | 8.44 – 10.28 (3) | 4.8 – 5.2 (4) | 6.34 – 6.76 (4) | 2.34 – 2.72 (4) |
| *P. exudus* | 27.82 | 7.82 | 4.62 | 6.22 | 2.24 |
| *P. orientalis* | 33.56, 35.42 | 10.06, 10.3 | 5.4, 5.6 | 7.54, 7.46 | 2.6, 2.7 |
| *P. lichtenstein* | 27.9, 30.02 | 7.66, 7.8 | 4.78*, 7.8 | 5.9*, 5.6 | 2.12, 2.24 |
| *P. senegalensis* | 27.52 | 7.9 | 4.66 | 5.92 | 2.5 |
| *Syrrhaptes paradoxus* | 28.34, 26.28 | 9.56, 8.52 | 5.28, 5.14 | 7.1, 6.8 | 2.28, 2.36 |

* - Appears to be pathological.

**Table G. Measurements of owl tarsometatarsi from the Pin Hole MIS 3 level in relation to those of those of modern specimens [53**].

|  | **Tarsometatarsus** | | | | | |
| --- | --- | --- | --- | --- | --- | --- |
|  | **GL** | **Bp** | **KC** | **Tp** | **Td** | **Bd** |
| L. tmt. (PH(F) 7837 and 7838) | 45.37 | 9.67 | 4.85 | 8.58 | 8.81 | 10.58 |
| R. tmt (PH(F) 7837 and 7838) | 44.5 | 9.15 | 4.74 | 8.42 | 8.22 | - |
| *Tyto alba* | 55.4 – 64.6 (29) | 8.4 – 9.9 (29) | 3.5 – 4.3 (29) | 6.8 – 8.6 (21) | 7.5 – 8.8 (21) | 9.5 – 11.2 (28) |
| *Strix aluco* | 45.5 – 50.1 (25) | 8.9 – 10.7 (26) | 4.7 – 5.9 (26) | 8.3 – 10.7 (26) | 7.8 – 9.9 (25) | 10.3 – 12.1 (25) |
| *Asio otus* | 36.8 – 41.5 (55) | 7.6 – 9.1 (55) | 3.8 – 4.8 (55) | 7.3 – 8.5 (43) | 6.4 – 7.6 (43) | 8.7 – 10.6 (55) |
| *Asio flammeus* | 41.1 – 48.7 (22) | 8.1 – 9.8 (23) | 3.7 – 5.1 (23) | 7.2 – 12.5 (20) | 6.6 – 8.1 (20) | 9.1 – 11.1 (23) |

**Table H. Measurements of the carpometacarpi of Turdidae showing the Pin Hole MIS 3 specimen in relation to extant *Turdus* species [36**].

|  | **Pin Hole** | **Modern Comparative Data** | | | | | |
| --- | --- | --- | --- | --- | --- | --- | --- |
|  | PH(F) 9069 to 9071  20.08  5.22 | *Turdus iliacus* | *T. philomelos* | *T. merula* | *T. torquatus* | *T. pilaris* | *T. viscivorus* |
| Carpometacarpus Greatest Length GL |  | 17.92 – 19.62 (19) | 18.04 – 19.78  (20) | 19.58 - 21.22 (21) | 22.26 | 22.66 – 25.1 (16) | 22.04 – 25.78 (9) |
| Carpometacarpus Proximal Breadth BP |  | 4.56 – 5.16 (19) | 4.28 – 5.32  (20) | 5.02 - 6.03 (21) | 5.56 | 5.82 – 6.62 (16) | 6 – 6.92 (9) |

Table I. Skeletal element representation of Galliformes and Anatinae at Pin Hole compared to West Runton, Boxgrove and the La Fage ravens (*Corvus antecorax*) [37,38,39, 54].

| Skeletal element | % if elements equally represented | % (No.) | | | | | |
| --- | --- | --- | --- | --- | --- | --- | --- |
|  |  | Pin Hole MP | | West Runton  Anatinae | Boxgrove Quarry 2  Anatinae | Boxgrove Quarry 1  Anatinae | La Fage  *C. corax antecorax* |
|  |  | Galliformes | Anatinae |  |  |  |  |
| Humerus | 11.1 | 13.8(9) | 32.3 (10) | 16.7 | 14.3 | 13.4 | 8.7 |
| Ulna | 11.1 | 3.1 (2) | 6.5 (2) | 3.7 | 17.1 | 11.9 | 15.7 |
| Radius | 11.1 | 0 | 3.2 (1) | 7.4 | 8.6 | 4.5 | 9.2 |
| Carpometacarpus | 11.1 | 6.2 (4) | 12.9 (4) | 18.5 | 11.4 | 14.9 | 14.5 |
| Coracoid | 11.1 | 1.5 (1) | 12.9 (4) | 31.5 | 34.3 | 32.8 | 11.2 |
| Scapula | 11.1 | 1.5 (1) | 3.2 (1) | 7.4 | 11.4 | 11.9 | 4.9 |
| Femur | 11.1 | 9.2 (6) | 0 | 0 | 0 | 0 | 11.8 |
| Tibiotarsus | 11.1 | 6.2 (4) | 9.7 (3) | 7.4 | 0 | 4.5 | 11.7 |
| Tarsometatarsus | 11.1 | 58.5 (38) | 19.4 (6) | 7.4 | 2.9 | 2.9 | 12.5 |

**
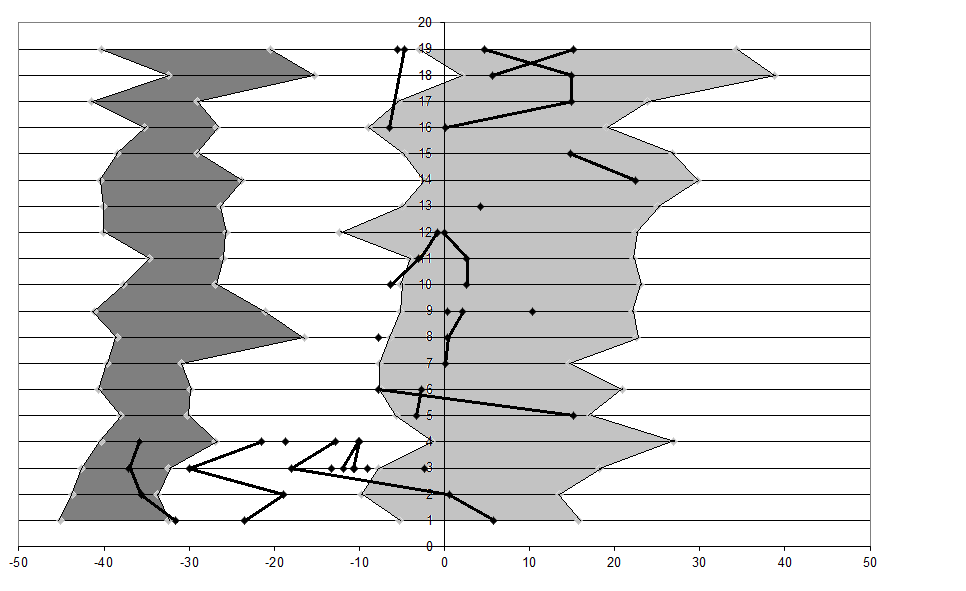
**

Tibiotarsus Dd

Tarsometatarsus GL

Tarsometatarsus Bp

Tarsometatarsus Bd

Tarsometatarsus SC

518, 524, 42, 238

13084

7609 - 7611

7591

18597

Tibiotarsus Bd

518, 524, 42, 238

Scapula Dic

*A. crecca*

Coracoid BF

651, 141

Coracoid Lm

7812

*A. platyrhynchos*

Coracoid GL

Carpometacarpus Bd

145

Carpometacarpus Bp

8075

No no.

No no.

Carpometacarpus GL

7806

Ulna Did

7810

759

Ulna GL

No no.

Humerus SC

13064

9074

8074

Humerus Bd

Humerus Bp

Humerus GL

7844

7805

518, 524,

42, 238

7807

651, 142

9075

**Figure A. Percentage ratio diagram of the Pin Hole MIS 3 anatine bone measurements. Ratios are expressed as percentage of a female mallard. Also shown are the modern range of the mallard *Anas platyrhynchos* for each measurement (the grey area in the middle of the diagram ) as well as that for the teal *A. crecca* (the smallest European duck, the grey area to the left of the diagram) both taken from [50]. All the numbers in the diagram are Pin Hole specimen numbers from the Armstrong excavation and would normally have the prefix PH(F).**

**Figure B. Scattergram with the tibiotarsus distal breadth measurement versus distal depth of the long-legged buzzard *Buteo* cf. *rufinus* from the Middle Palaeolithic of Pin Hole together with those of modern comparative specimens of European buzzards and hawks.**

**Figure C. Scattergram with carpometacarpus greatest length measurement versus proximal breadth of *Falco* cf. *tinnunculus* from Middle Palaeolithic of Pin Hole together with those of modern comparative specimens of smaller falcon.**

**
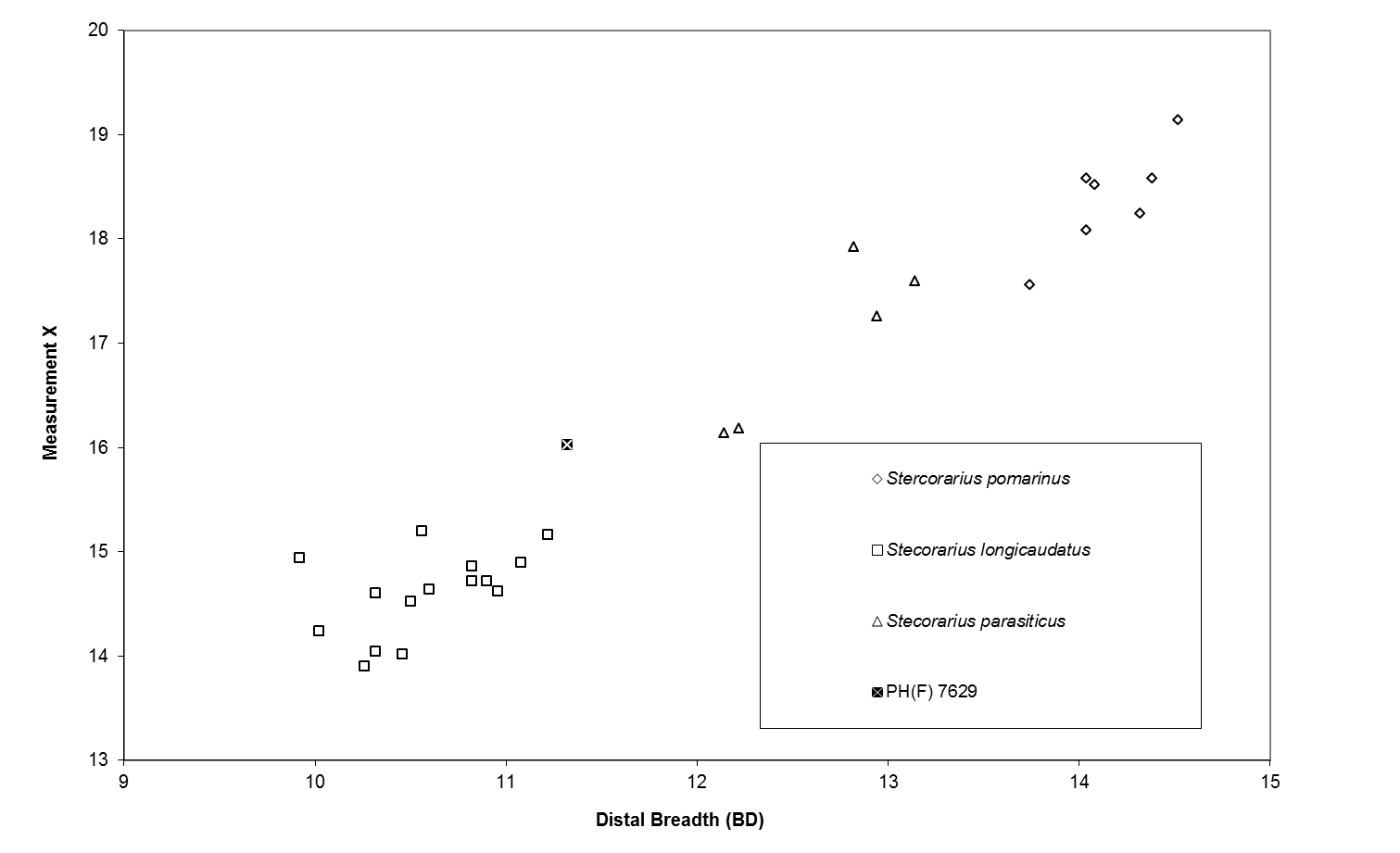
Figure D. Scattergram with humerus distal breadth measurement versus measurement X (see Figure K) of skua from Middle Palaeolithic of Pin Hole together with those of modern comparative specimens of *Stecorarius*.**

**
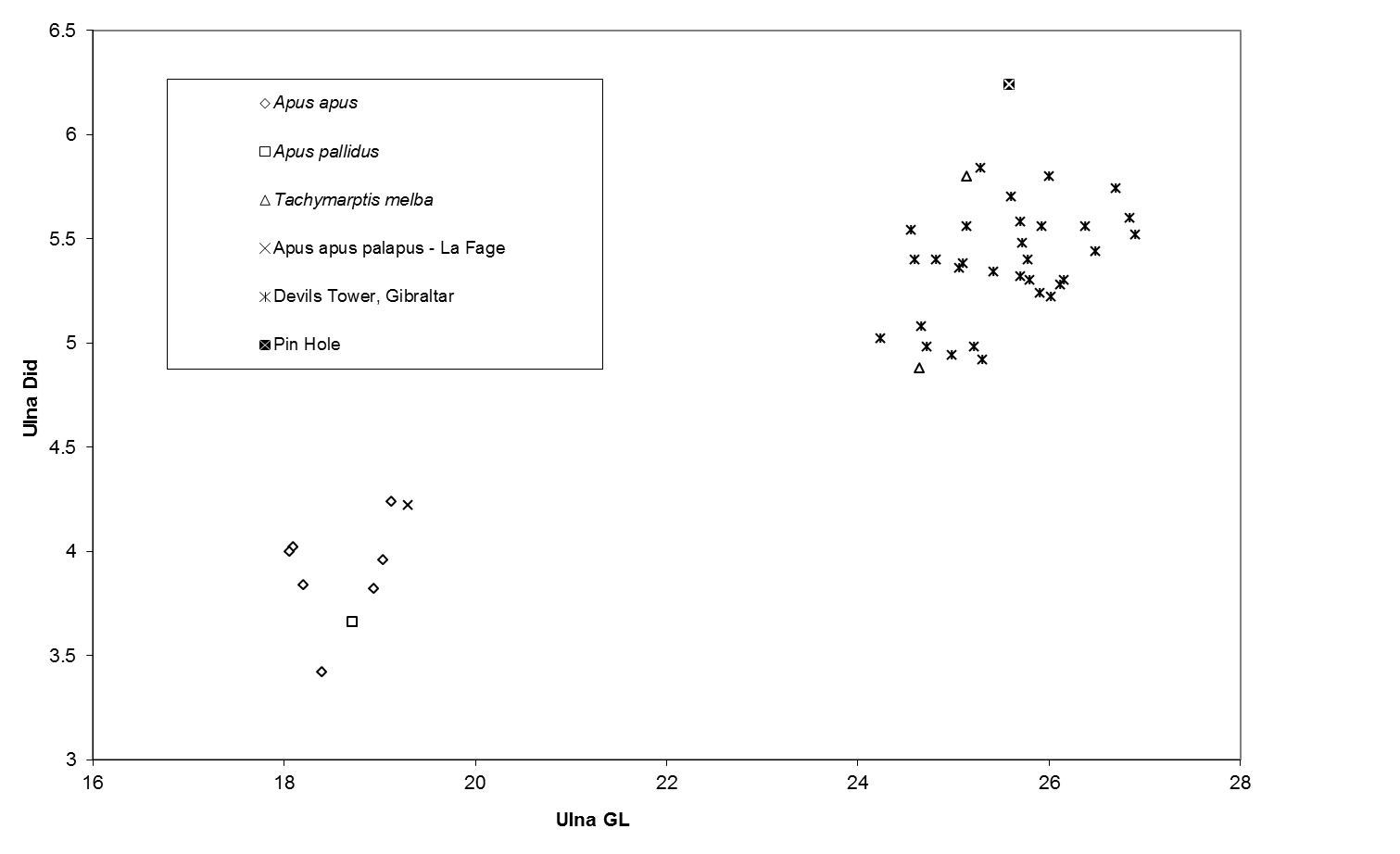
**

**Figure E. Scattergram with ulna greatest length measurement versus distal depth of *Tachymarptis melba* from Middle Palaeolithic of Pin Hole together with those of modern comparative and Pleistocene specimens of swifts Apodiformes.**

**
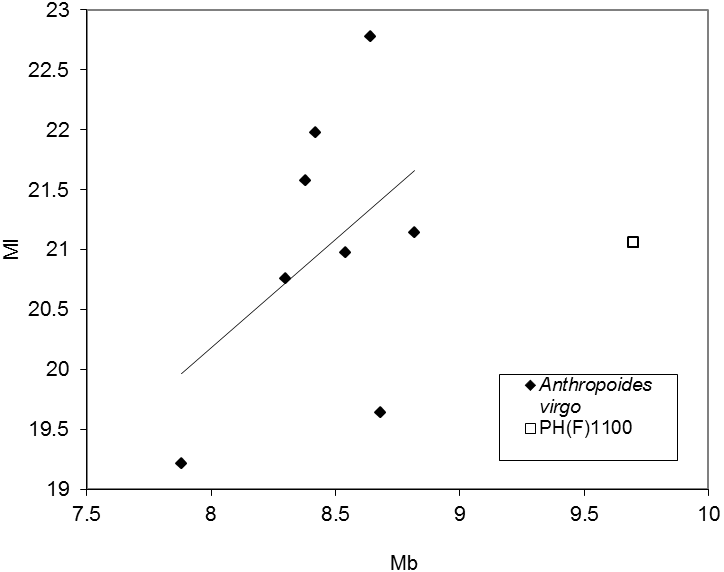
**

y = 1.7987x + 5.7973

R^2^ = 0.1986

**Figure F. Scattergram with measurements (Mandible length Ml and Mandible breadth Mb, see Figure K) taken on the premaxilla of *Anthopoides virgo* from Middle Palaeolithic of Pin Hole together with those of modern comparative specimens of the species.**


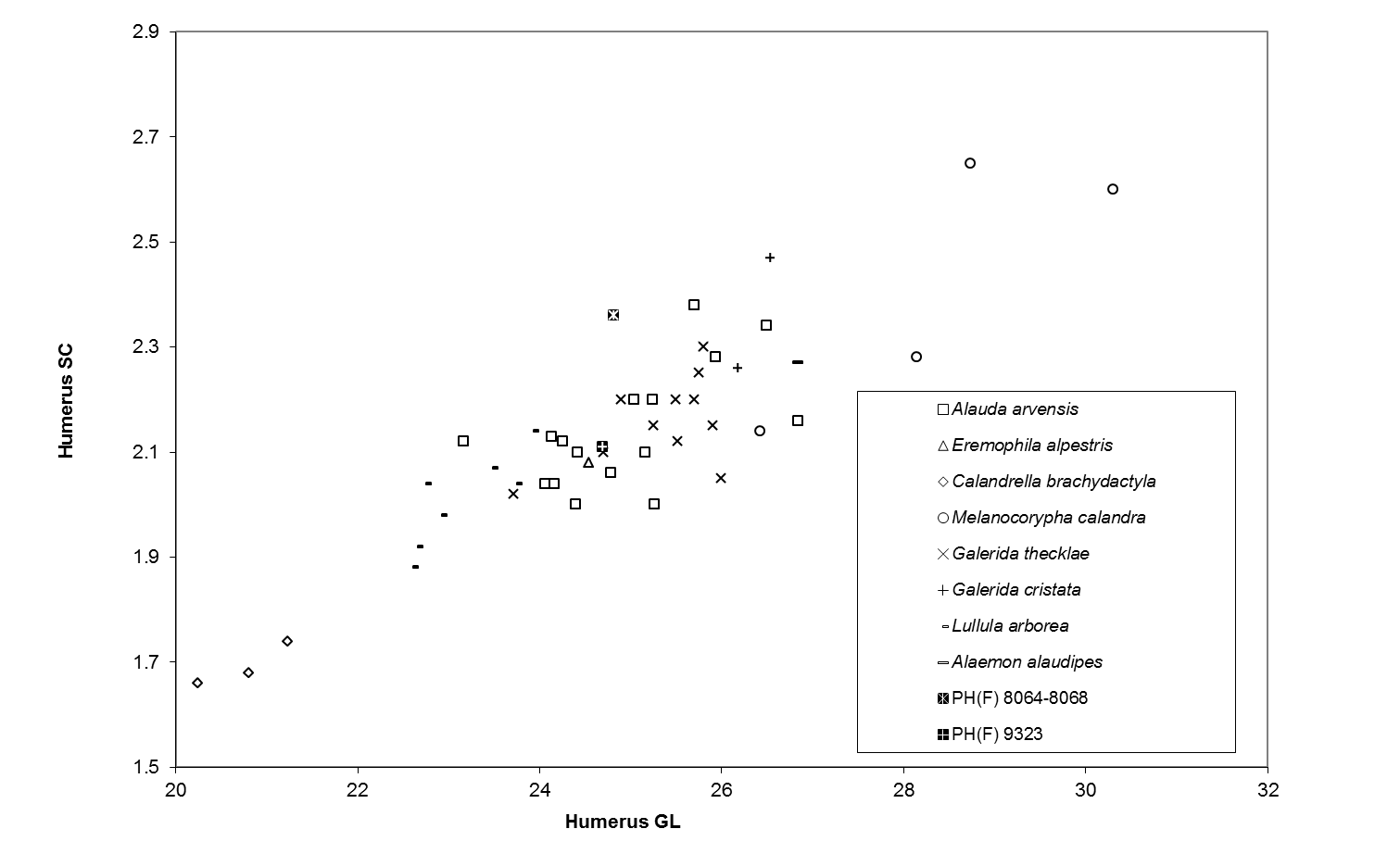


**Figure G. Scattergram with humerus greatest length measurement versus shaft breadth of Alaudidae from Middle Palaeolithic of Pin Hole together with those of modern comparative specimens of European larks.**


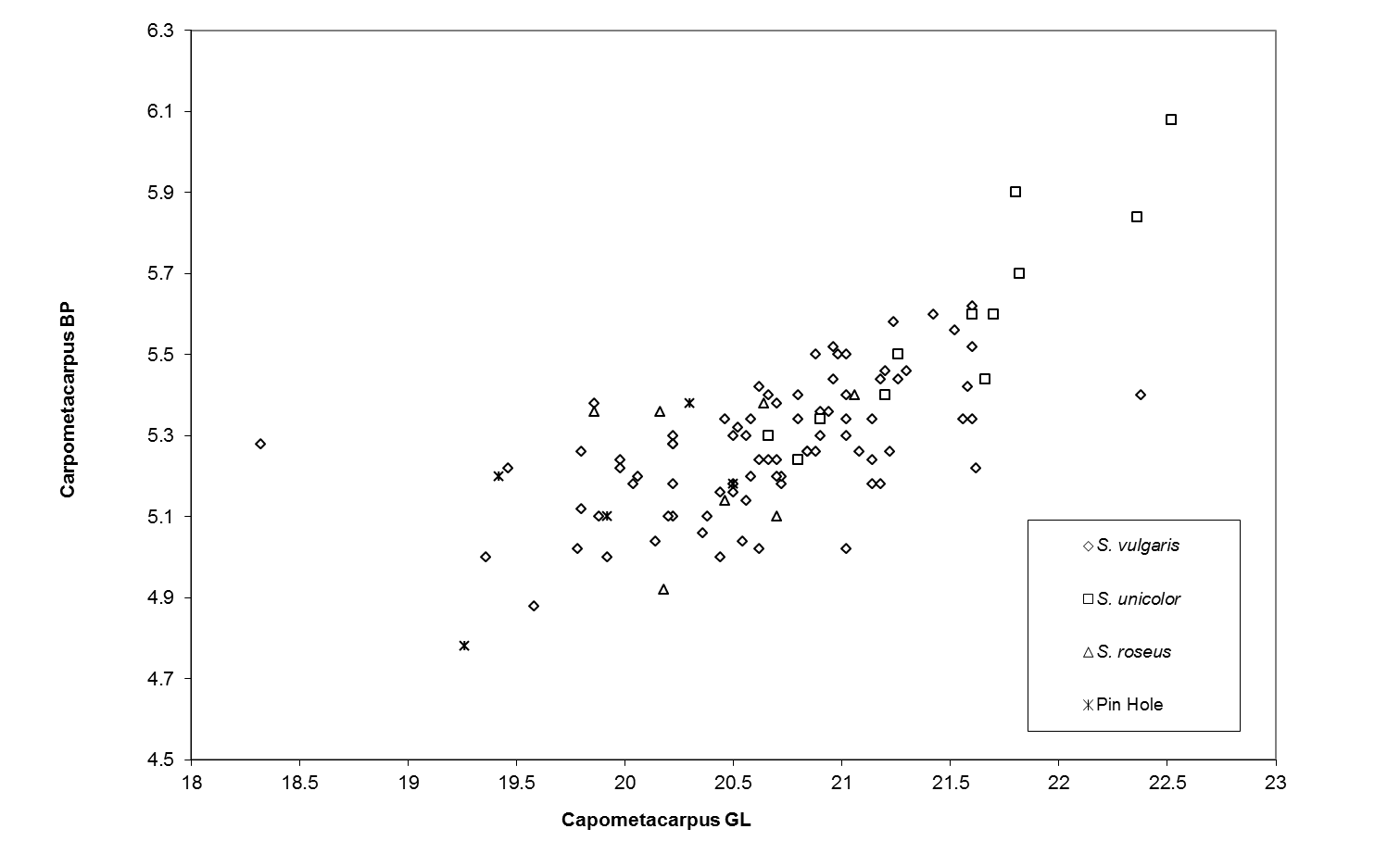


**Figure H. Scattergram with humerus proximal breadth measurement versus shaft breadth of starlings from Middle Palaeolithic of Pin Hole together with those of modern comparative specimens of European starlings.**

**Figure I. Scattergram with carpometacarpus greatest length measurement versus proximal breadth of raven *Corvus corax* from Middle Palaeolithic of Pin Hole together with those of modern comparative specimens of European ravens and the Middle Pleistocene raven *C. corax antecorax* from France.**

**Figure J. Scattergram with tarsometatarsus distal breadth measurement versus shaft breadth of raven *Corvus corax* from Middle Palaeolithic of Pin Hole together with those of modern comparative specimens of European ravens and the Middle Pleistocene raven *C*. *corax antecorax* from France.**

**
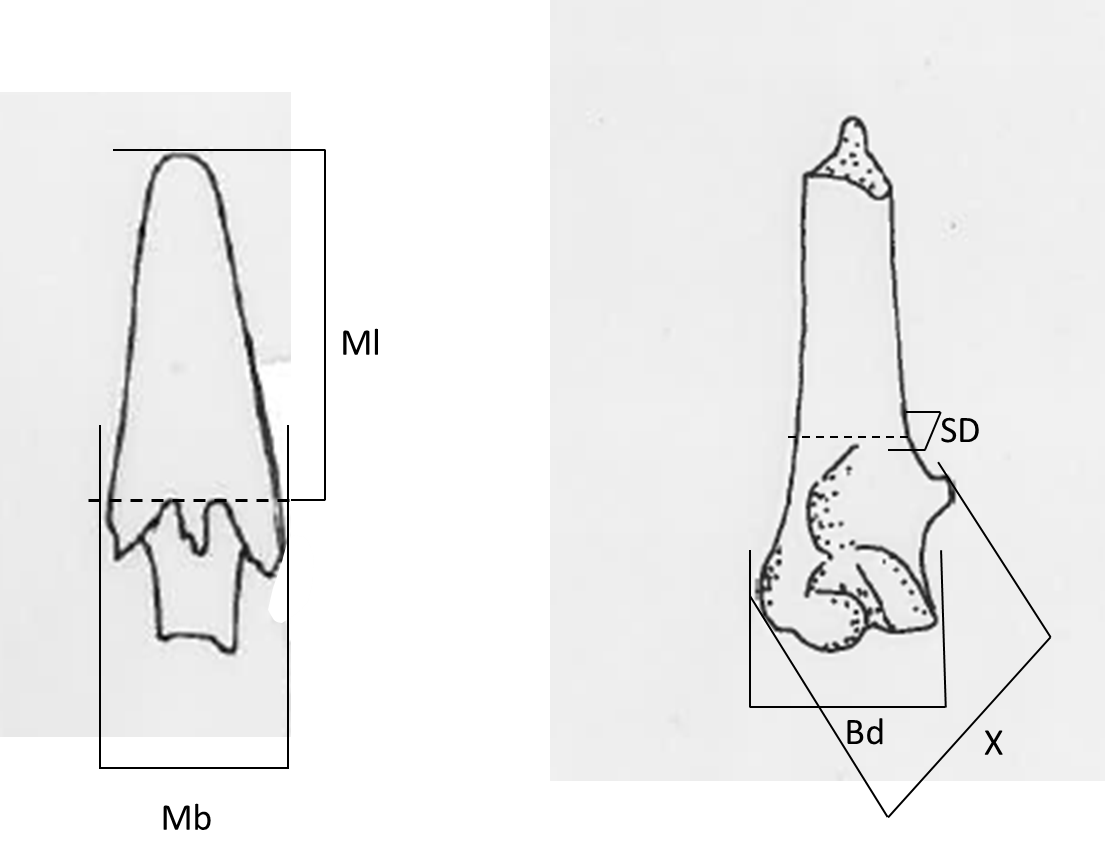
**

**Figure K. Measurements taken on *Anthropoides virgo* premaxilla and Stercorariidae distal humeri. *Anthropoides virgo* premaxilla: Mb – premaxilla length from distal ends of the concavitas palati to distal tip, Ml – premaxilla breadth at distal ends of the concavitas palati; Stercorariidae distal humerus: Bd - breadth of distal humerus as described in figure; X - diagonal measurement from processus supracondylaris dorsalis to epicondylus ventralis; SD – depth of humerus shaft at distal end at the proximal margin of the fossa musculi brachialis.**
